# Five new secondary metabolites from an endophytic fungus *Phomopsis* sp. SZSJ-7B

**DOI:** 10.3389/fpls.2022.1049015

**Published:** 2022-11-14

**Authors:** Yan Chen, Huan Wang, Xin Ke, Zihuan Sang, Min Kuang, Weiwei Peng, Jianbing Tan, Yuting Zheng, Zhenxing Zou, Haibo Tan

**Affiliations:** ^1^ Xiangya School of Pharmaceutical Sciences, Central South University, Changsha, China; ^2^ Hunan Key Laboratory of Diagnostic and Therapeutic Drug Research for Chronic Diseases, Central South University, Changsha, China; ^3^ Key Laboratory of South China Agricultural Plant Molecular Analysis and Genetic Improvement, Guangdong Provincial Key Laboratory of Applied Botany, South China Botanical Garden, Chinese Academy of Sciences, Guangzhou, China

**Keywords:** endophytic fungus, *Phomopsis*, secondary metabolites, antibacterial activity, cytotoxic activity

## Abstract

Two previously undescribed lactones, phomolides A and B (**1** and **2**), and three new sesquiterpenoids, phomenes A–C (**3**–**5**), together with one known compound, colletotricholide A (**6**), were isolated from the endophytic fungus *Phomopsis* sp. SZSJ-7B. Their chemical structures, including the absolute configurations, were comprehensively established by extensive analyses of NMR, high-resolution electrospray ionization mass spectrometry, electronic circular dichroism powered by theoretical calculations, and X-ray diffractions. Moreover, the cytotoxic and antibacterial activities of compounds **1–6** were also evaluated, and the results demonstrated that compound **2** showed significant antibacterial effects towards methicillin-resistant *Staphylococcus aureus* and *S. aureus* strains with minimum inhibitory concentration as low as 6.25 μg/ml, which was comparable to that of the clinical drug vancomycin. Moreover, all compounds showed no cytotoxic activity.

## 1 Introduction

Endophytes play an important role of producing biologically meaningful natural products and can be considered as a strategically promising bio-resource of significantly economic potential for the agrochemical and pharmaceutical industries (Gouda et al., 2016). It is well documented that the genus *Phomopsis* can generate structurally diverse and pharmaceutically active secondary metabolites (Huang et al., 2008; Yu et al., 2008; Hemtasin et al., 2011), including xanthones (Ding et al., 2013), *α*-pyrones (Cai et al., 2017), steroids (Hu et al., 2017), sesquiterpenes (Xie et al., 2018), diterpenes (Wei et al., 2014), triterpenes (Li et al., 2008), oblongolides (Bunyapaiboonsri et al., 2010), pyrenocines (Hussain et al., 2012), alkaloids (Chen et al., 2019), cytochalasins (Yan et al., 2016), *etc*. Moreover, these intriguing natural compounds shared various biological activities such as antitumor (Pavao et al., 2016), immunosuppressive (Wei et al., 2014), antifungal (Krohn et al., 2011), antioxidant (Chen et al., 2018), antibacterial (Jouda et al., 2016), anti-inflammatory (Xu et al., 2019b), and *α*-glucosidase inhibitory effects (Huang et al., 2018).

Our group pursued continuous research commitments towards discovering structurally fascinating and biologically significant natural products from endophytic fungi in recent years, and a series of metabolites with excellent antibacterial and antitumor activities from endophytic fungi of the genus *Phomopsis* have been reported (Xu et al., 2019a; Chen et al., 2020; Liu et al., 2021). In continuation of our ongoing endeavors, a strain of *Phomopsis* sp. SZSJ-7B isolated from the fresh leaves of *Alpinia shengzhen*, which is a beautiful horticultural plant (Zingiberaceae), was chosen as the appealing target for the chemical constituent investigation. Preliminary thin-layer chromatography and high-performance liquid chromatography (HPLC) screenings of the strain SZSJ-7B were conducted, and the experimental data showed that its ethyl acetate (EtOAc) extracts exhibited a remarkable diversity of secondary metabolites. A further systematical chemical study of the strain led to the isolation of five previously undescribed metabolites including two lactones, phomolides A and B, and three sesquiterpenoids, phomenes A–C. Herein the details of the extraction, purification, structure elucidation, and their biological evaluation are described.

## 2 Results and discussion

Compound **1** was isolated as a white solid. Its molecular formula C_11_H_12_O_4_ was deduced from high-resolution electrospray ionization mass spectrometry (HRESIMS) *m*/*z* 209.0815 [M + H]^+^ [calculated (calcd) for C_11_H_13_O_4_, 209.0808], indicating six degrees of hydrogen deficiency. The infrared (IR) spectrum of **1** showed obvious absorption bands at 3,325, 1,699, and 1,022 cm^-1^ and revealed the presence of corresponding hydroxyl and carbonyl functionalities together with the ether bonds. The ^1^H nuclear NMR data (**Table 1**) of **1** showed two singlet methyl groups (*δ*_H_ 2.12 and 2.57) and an upfield doublet methyl group (*δ*_H_ 1.65, d, *J* = 5.2 Hz). Its ^13^C NMR (**Table 1**) and heteronuclear single quantum coherence (HSQC) spectra showed 11 carbon signals including six quaternary carbons (*δ*_C_ 163.2, 161.9, 159.1, 143.0, 120.6, and 104.5), two oxymethine (*δ*_C_ 98.7 and 97.7), and three methyl groups (*δ*_C_ 18.7, 16.1, and 10.0).

**Table 1:** ^1^H (500 MHz) and ^13^C (125 MHz) NMR data of 1 and 2 in CD_3_OD (*δ* in ppm, *J* in Hz).

1			2					
No.	*δ*_H_ (*J* in Hz)	*δ*_C_	No.	*δ*_H_ (*J* in Hz)	*δ*_C_	No.	*δ*_H_ (*J* in Hz)	*δ*_C_
1	5.64, q, (5.2)	97.7, CH	1	2.24, m 1.93, m	31.8, CH_2_	13	2.95, s	46.5, CH_2_
2		163.2, C	2	1.31, m	29.1, CH_2_	14	0.95, s	19.9, CH_3_
3		104.5, C	3	1.55, m 1.47, m	31.3, CH_2_	15	0.84, d, (6.5)	14.7, CH_3_
4		159.1, C	4	1.72, dt, (6.5, 10.6)	38.5, CH	1’		164.1, C
5	6.32, s	98.7, CH	5		39.0, C	2’		104.1, C
6		161.9, C	6*α* 6*β*	2.19, m 1.03, m	34.8, CH_2_	3’		160.9, C
7		120.6, C	7	2.44, m	28.3, CH	4’	6.35, d, (2.0)	99.8, CH
8		143.0, C	8*α* 8*β*	1.83, m 2.12, m	28.5, CH_2_	5’		161.6, C
9	2.57, s	16.1, CH_3_	9	5.34, m	116.8, CH	6’	6.50, d, (2.0)	114.4, CH
10	2.12, s	10.0, CH_3_	10		146.7, C	7’		145.9, C
11	1.65, d, (5.2)	18.7, CH_3_	11		59.5, C	8’	2.57, s	20.8, CH_3_
			12	5.46, s	100.8, CH			

**Figure 1:**
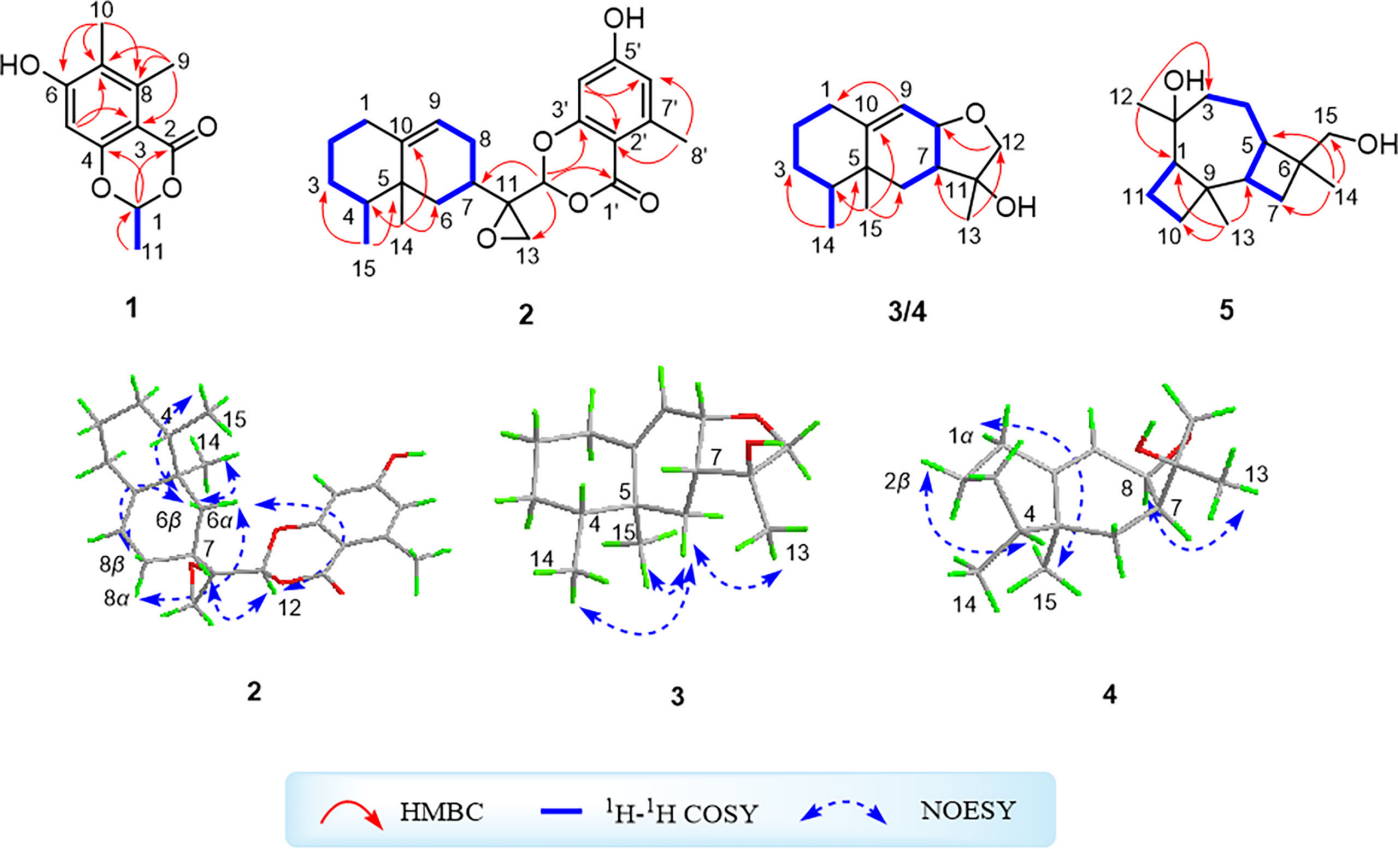
^1^H–^1^H correlation spectroscopy and key heteronuclear multiple bond correlation correlations of compounds **1**–**5** and key nuclear Overhauser effect spectroscopy correlations of compounds **2**–**4**.

**Figure 2:**
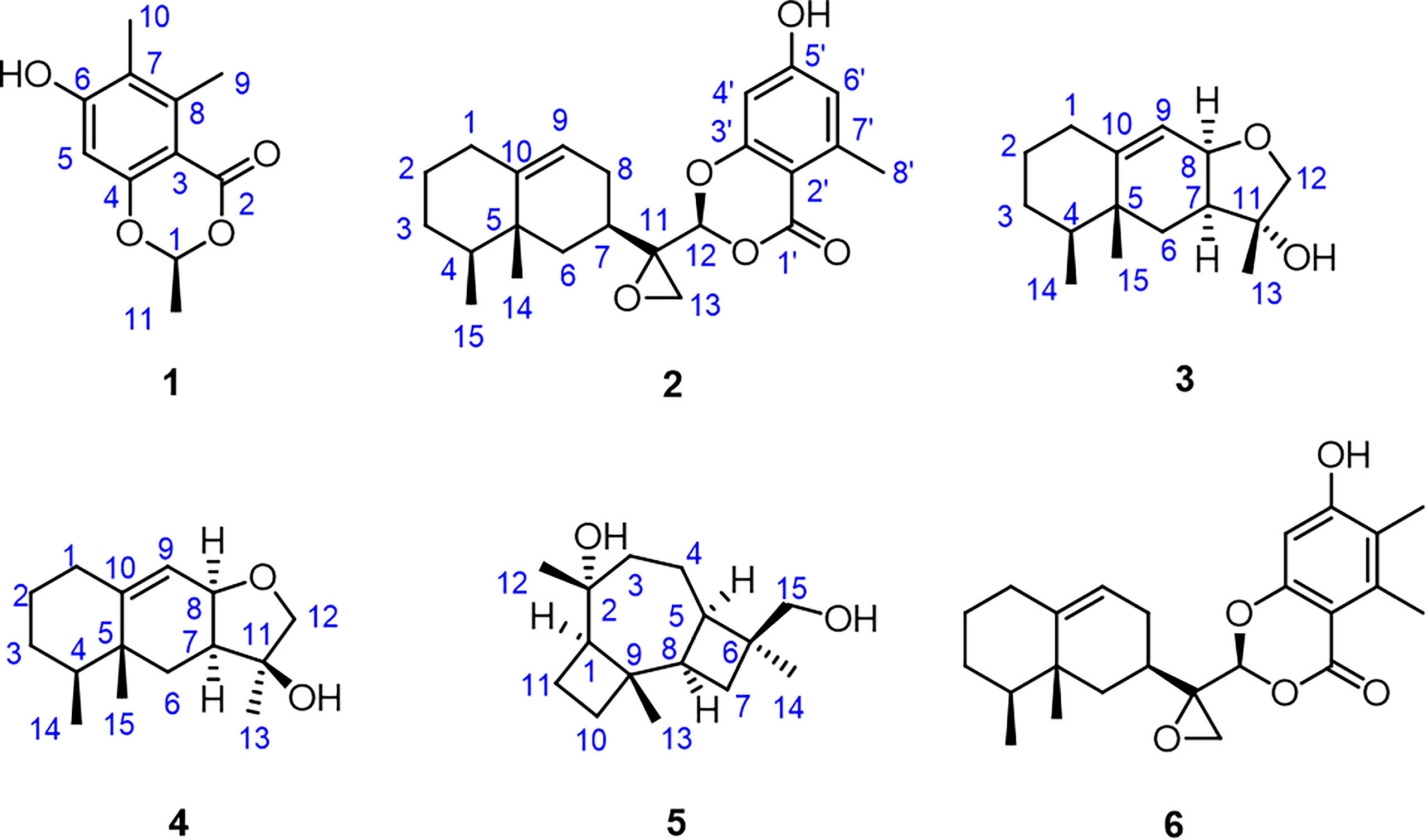
Structures of compounds **1–6**.

**Figure 3:**
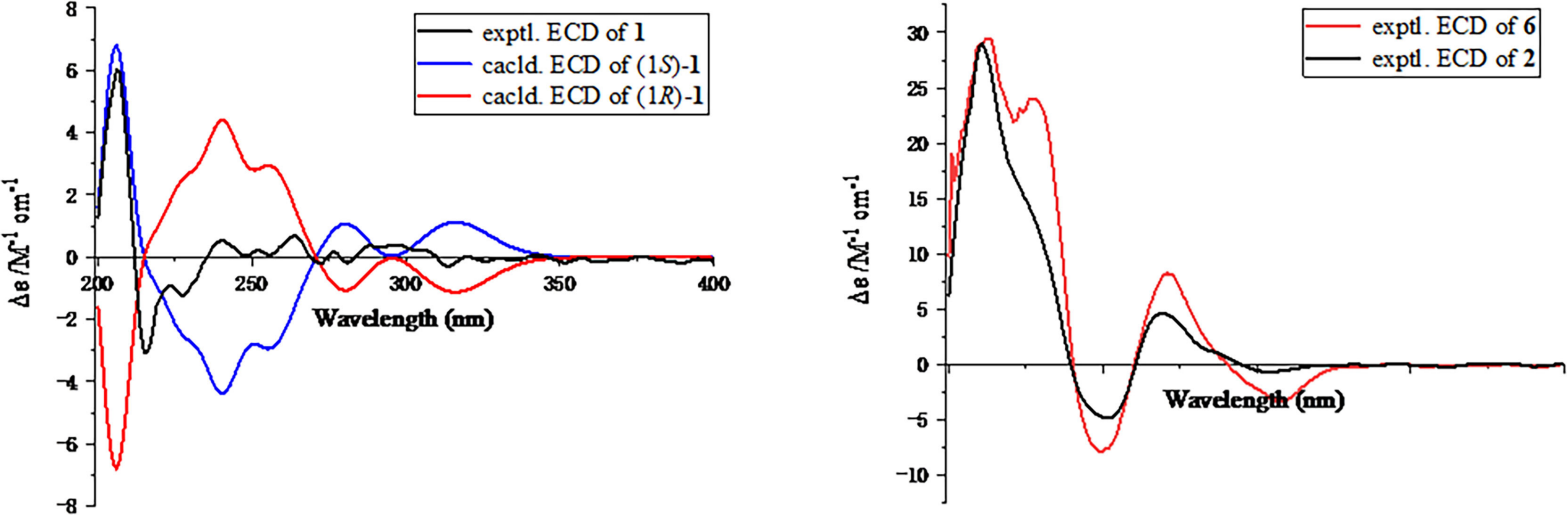
Experimental and calculated electronic circular dichroism spectra of compounds **1, 2,** and **6**.

Moreover, the significant heteronuclear multiple bond correlation (HMBC) correlations (**Figure 1**) from H_3_-9 to C-3 (*δ*_C_ 104.5), C-7 (*δ*_C_ 120.6), and C-8 (*δ*_C_ 143.0), H_3_-10 to C-6 (*δ*_C_ 161.9), C-7, and C-8, and H-5 to C-3 and C-7 strongly suggested the existence of a penta-substituted benzene ring. In addition, on the basis of the ^1^H–^1^H correlation spectroscopy (COSY) fragment of C-1/C-11, the obvious HMBC correlations from H_3_-11 to C-1 (*δ*_C_ 97.7) coupling with H-1 to C-2 (*δ*_C_ 163.2) and C-4 (*δ*_C_ 159.1) conclusively confirmed the planar structure of **1** as shown in **Figure 2**. In order to further clarify the absolute configuration, electronic circular dichroism (ECD) calculation of **1** was performed on mPW1PW91/SVP level of theory. As a result, the experimental ECD spectrum perfectly matched with the calculated ECD spectrum of 1*S* configuration for **1**, showing the same clear Cotton effect at 205 nm. Thus, the absolute configuration of **1** was determined to be 1*S* (**Figure 3**), and it was revealed as a new, natural, rarely occurring acetal derivative, which was given the trivial name phomolide A.

Compound **2** was isolated as a colorless oil. Its molecular formula C_23_H_28_O_5_ was deduced from its HRESIMS spectrum with a molecular ion peak observed at *m*/*z* 385.2007 [M + H]^+^ (calcd for C_23_H_29_O_5_, 385.2010), which chemologically implied 10 degrees of hydrogen deficiency. Moreover, the IR spectrum of compound **2** showed a series of characteristic absorption bands at 3,357, 1,714, and 1,020 cm^-1^, which were attributed to hydroxyl and carbonyl functional moieties as well as ether bonds. The ^1^H NMR data (**Table 1**) of **2** showed typical proton resonances for three methyl groups at *δ*_H_ 0.95 (s, H_3_-14), 0.84 (d, *J* = 6.5 Hz, H_3_-15), and 2.57 (s, H-8’), an oxygenated methine moiety at *δ*_H_ 5.46 (s, H-12), and two aromatic protons at *δ*_H_ 6.35 (d, *J* = 2.0 Hz, H-4′) and *δ*_H_ 6.50 (d, *J* = 2.0 Hz, H-6′) together with an olefinic proton at *δ*_H_ 5.34 (m, H-9). The ^13^C NMR (**Table 1**) and HSQC spectra exhibited 24 carbon signals comprising three methyls, six methylenes, six methines, and nine quaternary carbons. The ^1^H–^1^H COSY spectrum of **2** revealed the existence of two independent spin systems of H_2_-1/H_2_-2/H_2_-3/H-4/H_3_-15 and H_2_-6/H-7/H_2_-8/H-9.

Compound **2** was further conclusively revealed as a novel meroterpenoid with eremophilan and acetophenone units conjugating as an acetal skeleton after the careful comparison of 1D NMR data of **2** with those of the known compound colletotricholide A **(6)** (Zhao et al., 2020), which was also co-isolated from this strain. The main difference in NMR spectra between **2** and colletotricholide A **(6)** was attributed to the lack of a methyl group in **2** at C-6’ position. This speculation could be further verified by the ^1^H and ^13^C NMR signals for H-6’ (*δ*_H_ 6.50) and C-6’ (*δ*_C_ 114.4) in **2** and the key HMBC correlations from H-6’ to C-1’ (*δ*_C_ 164.1) and C-4’ (*δ*_C_ 99.8). Therefore, the planar structure of **2** was identified as shown in **Figure 2**.

The relative configuration of **2** was determined by the nuclear Overhauser effect spectroscopy (NOESY) experiment (**Figure 1**). As shown in **Figure 1**, the NOESY correlations of H-6*β* with H-8*β*, H_3_-14, and H_3_-15 revealed that these protons were on the same face and assumed as *β*-oriented, while the correlations of H-6*α* with H-8*α* together with H-12 with H-7 and H-8*α* indicated that H-7 and H-12 were *α*-oriented. The CD spectrum of **2** showed positive Cotton effects at 211 and 268 nm and negative Cotton effect at 252 nm, which were very similar with those in the CD spectrum of the known compound **6**. By comparing the CD curves of compounds **2** and **6** (**Figure 3**), it could be determined that compounds **2** and **6** ought to share the same absolute configuration. Therefore, the absolute configuration of compound **2** was designed as 4*S*,5*R*,7*R*,11*R*,12*S* and given the trivial name phomolide B.

Compound **3** was isolated as a yellow oil. The molecular formula of **3** was determined to be C_15_H_24_O_2_ by the HRESIMS analysis, indicating four degrees of hydrogen deficiency. Compound **3** exhibited obvious absorption bands at 3,363 and 1,024 cm^-1^ in the IR spectrum, which indicated the presence of hydroxyl group and ether bond. The ^1^H NMR data of **3**, as shown in **Table 3**, illustrated two singlet methyl functional groups (*δ*_H_ 0.95 and 1.33) and a doublet methyl group (*δ*_H_ 0.82, d, *J* = 6.6 Hz). The ^13^C NMR (**Table 3**), supported with the HSQC of **3**, indicated the presence of 15 carbon atoms attributed to three methyl groups (*δ*_C_ 15.7, 20.3, and 20.8), five methylene groups (*δ*_C_ 29.8, 30.9, 32.1, 32.2, and 78.6), four methine groups (*δ*_C_ 38.0, 45.1, 75.1, and 115.6), and three quaternary carbons (*δ*_C_ 39.3, 81.8, and 153.7). All the aforementioned conclusive information collectively indicated that compound **3** is a sesquiterpene derivative.

The ^1^H–^1^H COSY spectrum of **3** which displayed two consecutive correlations of H_2_-1/H_2_-2/H_2_-3/H-4/H_3_-14 and H_2_-6/H-7/H-8/H-9 successfully suggested the presence of two independent substructures **a** (C-1/C-2/C-3/C-4/C-14) and **b** (C-6/C-7/C-8/C-9). The further comparison of the 1D NMR spectroscopic data (**Table 2**) with those of the known compound cyclodebneyol (Burden et al., 1986) tentatively revealed that compound **3** shared the same planar structure as that of the previously reported natural product cyclodebneyol. Moreover, the key HMBC correlations from H_3_-14 to C-3 (*δ*_C_ 30.9) and C-5 (*δ*_C_ 39.3); H_3_-15 to C-6 (*δ*_C_ 32.2), C-4 (*δ*_C_ 38.0), and C-10 (*δ*_C_ 153.7); and H_3_-13 to C-7 (*δ*_C_ 45.1) and C-12 (*δ*_C_ 78.6), combined with the COSY fragments **a** and **b**, further confirmed the aforementioned conclusion (**Figure 1**).

**Table 2:** Calculated ^13^C chemical shifts (CDCl_3_) fitting with the experimental data of compounds 3a, 3b, 4a, and 4b following the STS protocol.

Exptl.	3				Exptl.	4			
	3a	Dev	3b	Dev		4a	Dev	4b	Dev
32.1	32.07	0.03	32.87	0.67	32.4	32.61	0.21	32.43	0.03
31.43	28.74	1.06	27.08	4.22	29.9	26.00	3.90	28.66	1.24
25.58	29.32	1.58	29.77	2.61	30.9	29.53	1.37	29.54	1.36
28.29	37.68	0.32	43.67	4.34	38.5	42.78	4.28	39.90	1.40
42.34	40.00	0.70	42.28	1.64	39.1	38.58	0.52	39.19	0.09
40.94	32.42	0.22	35.00	1.37	30.4	35.41	5.01	31.99	1.59
33.57	47.18	2.08	49.33	2.96	43.4	44.00	0.60	44.03	0.63
48.06	74.93	0.17	78.33	2.26	75.5	75.38	0.12	75.44	0.06
77.36	118.92	3.32	123.75	7.66	115.6	121.20	5.60	119.66	4.06
123.26	152.23	1.47	146.34	7.61	154.6	150.01	4.59	152.02	2.58
146.09	81.55	0.25	78.38	4.39	79.4	79.36	0.04	79.00	0.40
77.41	75.86	2.74	81.08	1.54	78.6	76.83	1.77	77.44	1.16
80.14	21.44	0.64	24.16	1.82	27.7	26.34	1.36	26.15	1.55
22.62	15.88	0.18	15.93	1.39	15.8	16.64	0.54	16.11	0.31
14.31	20.77	0.47	19.19	2.69	20.3	17.74	2.56	20.56	0.26
17.61	MAE^a^	1.02	MAE^a^	3.14		MAE^a^	2.16	MAE^a^	1.11
	RMS^b^	1.42	RMS^b^	3.77		RMS^b^	2.89	RMS^b^	1.54
	*P*_mean_	32.34%	*P*_mean_	0.58%		*P*_mean_	3.12%	*P*_mean_	31.18%
	*P*_rel_	100.00%	*P*_rel_	0.00%		*P*_rel_	0.00%	*P*_rel_	100.00%

a Absolute error.

b Root mean square.

The obvious differences in the chemical shifts of both ^1^H and ^13^C NMR data between **3** and cyclodebneyol suggested that these two compounds ought to be a pair of closely related stereoisomers. Moreover, the cross-peaks of H_3_-14/H-6*β*, H_3_-13/H-6*β*, and H_3_-15/H-6*β* in the NOESY spectrum were clearly distinguished; thus, it could be readily speculated that the three methyls H_3_-13, H_3_-14, and H_3_-15 in **3** directed on the same side in its 6/6/5 fused ring skeleton and assumed as *β*-oriented. However, the proton chemical shift of H-7 was heavily overlapped with H-2*β*, so the NOESY correlations of H-7/H-4 could not conclusively determine the orientation of protons H-7 and H-4 to further completely confirm the final relative configuration of **3**.

**Figure 4:**
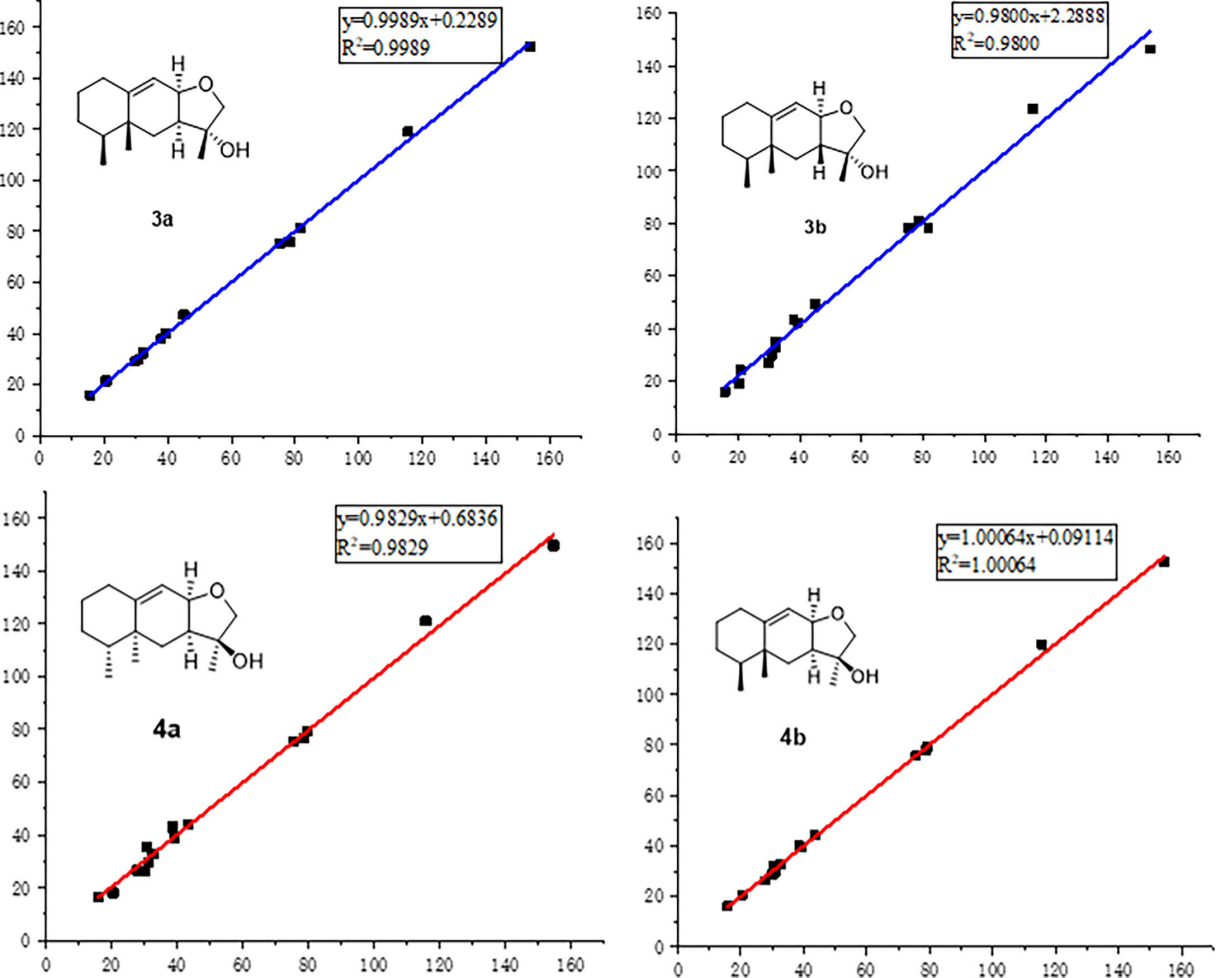
Regression analyses of experimental and calculated ^13^C NMR chemical shifts for **3a**, **3b**, **4a**, and **4b**.

In order to absolutely determine the relative configuration of C-7 for **3**, the gauge-independent atomic orbital (GIAO) density functional theory (DFT) ^13^C NMR calculations (McWeeny, 1961; Ditchfield, 1972) towards the structures **3a** and **3b** were performed at the *ω*B97x-D/6-31G* (Chai and Head-Gordon, 2008) (IEFPCM, CDCl_3_) level, and the calculation data were then compared with their experimental ^13^C NMR data following the reported sorted training set (STS) protocol (Li et al., 2020). According to the linear regression analysis of ^13^C NMR chemical shifts, the values of the correlation coefficient (*R* ^2^) were 0.9989 for **3a** and 0.9800 for **3b** (**Figure 4**). Moreover, the resulting *P*_rel_ value of **3a** is 100%, and the mean absolute error (MAE), root mean square error (RMSE), and *P*_mean_ values of **3a** showed that the calculated ^13^C NMR data match the experimental data very well, indicating that **3a** or its enantiomer is the correct structure for **3** (**Table 3**). With the aforementioned informative results, the relative structure of **3** was thus unambiguously established as shown in **Figure 2**.

**Table 3:** ^1^H (500 MHz) and ^13^C NMR (125 MHz) data of 3–5 in CDCl_3_ (***δ*** in ppm, *J* in Hz).

No.	3		4		5	
	*δ*_H_ (*J* in Hz)	*δ*_C_	*δ*_H_ (*J* in Hz)	*δ*_C_	*δ*_H_ (*J* in Hz)	*δ*_C_
1*α* 1*β*	2.00, d, (12.1) 2.29, td, (4.3, 12.1)	32.1, CH_2_	2.01, d, (11.9) 2.31, td, (4.4, 11.9)	32.4, CH_2_	1.99, m	53.1, CH
2*α* 2*β*	1.25, m 1.85, m	29.8, CH_2_	1.26, m 1.88, m	29.9, CH_2_		74.0, C
3*α* 3*β*	1.48, m	30.9, CH_2_	1.49, m	30.9, CH_2_	1.31, m 1.94, m	46.3, CH_2_
4	1.54, m	38.0, CH	1.53, m	38.5, CH	1.56, m	21.5, CH_2_
5		39.3, C		39.1, C	2.14, ddd, (2.4, 9.4, 15.9)	49.6, CH
6*α* 6*β*	0.94, m 1.76, m	32.2, CH_2_	1.91, m	30.4, CH_2_		36.7, C
7*α* 7*β*	1.89, m	45.1, CH	1.88, m	43.4, CH	1.43, m 1.74, m	29.2, CH_2_
8	4.50, t, (5.3)	75.1, CH	4.18, t, (5.2)	75.5, CH	2.61, dd, (9.4, 15.9)	41.9, CH
9	5.55, d, (5.3)	115.6, CH	5.50, d, (5.2)	115.6, CH		42.4, C
10*α* 10*β*		153.7, C		154.6, C	1.54, m 1. 49, m	37.6, CH_2_
11*α* 11*β*		81.8, C		79.4, C	1.79, m 1.73, m	18.6, CH_2_
12*α* 12*β*	3.73, d, (9.6) 3.87, d, (9.6)	78.6, CH_2_	3.66, d, (8.9) 3.84, d, (8.9)	78.6, CH_2_	1.24, s	23.4, CH_3_
13	1.33, s	20.8, CH_3_	1.46, s	27.7, CH_3_	1.22, s	22.4, CH_3_
14	0.82, d (6.6)	15.7, CH_3_	0.84, d, (6.4)	15.8, CH_3_	1.22, s	25.4, CH_3_
15a 15b	0.95, s	20.3, CH_3_	1.01, s	20.3, CH_3_	3.37, d, (10.8) 3.54, d, (10.8)	68.2, CH_2_

**Figure 5:**
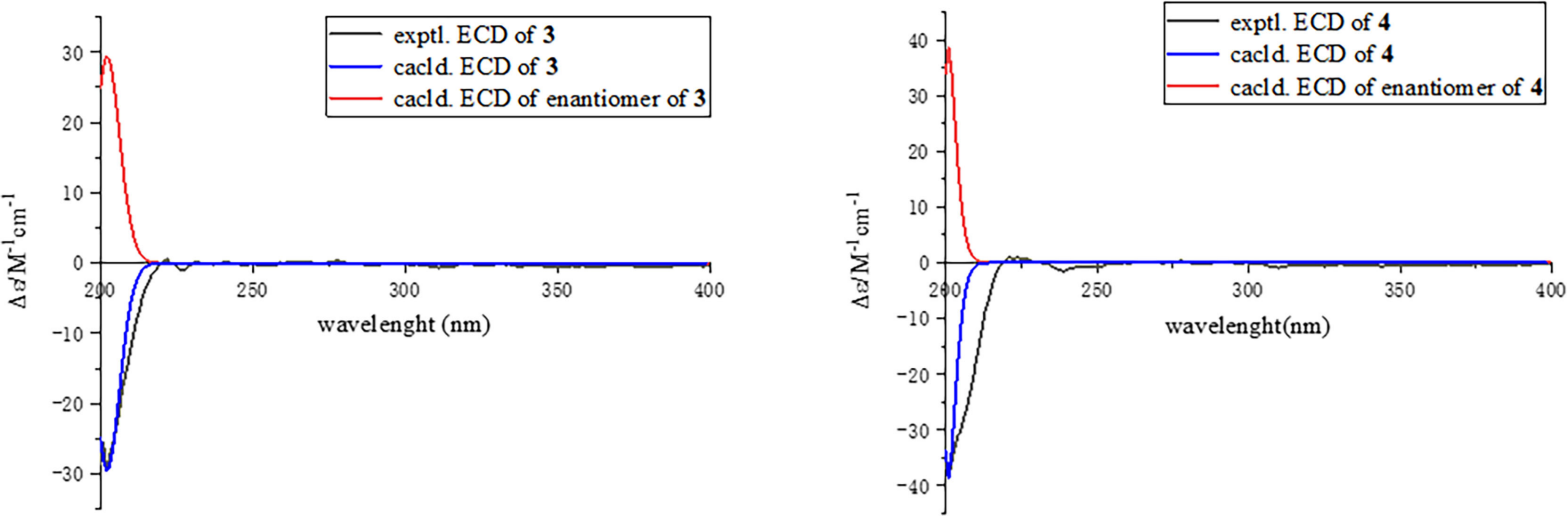
Experimental and calculated electronic circular dichroism spectra of compounds **3** and **4**.

Moreover, the absolute configuration of **3** was also determined by the time-dependent density-functional theory (TDDFT) calculated circular dichroism (CD) spectrum at the mPW1PW91/SVP level. As shown in **Figure 5**, the calculated ECD curve of 4*S*,5*R*,7*S*,8*R*,11*R* **-3** perfectly matched with the experimental ECD curve, which strongly suggested that compound 3 shared an absolute configuration of 4*S*,5*R*,7*S*,8*R*,11*R*. Therefore, the structure of compound **3** was completely established and given the trivial name phomene A.

Compound **4** was obtained as a yellow oil with the same molecular formula as **3,** which was determined by HRESIMS ion peak at *m*/*z* 237.1854. Obviously, the ^13^C NMR spectroscopic data (**Table 2**) and HSQC spectrum of **4** collectively suggested 15 carbon signals, and all of them showed very similar chemical shifts to those of **3**. The little differences between the chemical shifts of **3** and **4** in NMR spectra strongly implied that they should be a pair of diastereoisomers sharing the same planar structure. The further careful analysis of the NOESY spectrum of **4** could efficiently establish its relative configuration. Compared with the NOESY spectrum of **3,** a clear NOESY correlation of H-8/H_3_-13 could be readily found, illustrating that the critical protons H-8 and H_3_-13 were on the same side in the fused ring system and assumed as *α*-oriented. In addition, the cross-peaks of H-2*α*/H-4 and H-1*β*/H_3_-14 in the NOESY spectrum clearly demonstrated that the two methyls H_3_-14 and H_3_-15 were *β*-oriented. Therefore, the relative structure of **4** was tentatively assigned as a C-11 epimer of **3,** as shown in **Figure 2**, although the same stereochemical issue clouded the H-7 chirality as that of **3**.

In order to further confirm the relative configuration of **4,** we also carried out a ^13^C NMR calculation for **3.** As a result, the *P*_mean_ and *P*_rel_ parameters as well as MAE and RMS values further showed that **4b** or its enantiomer should be the correct structure for **4**, as shown in **Table 3**. Moreover, the absolute configuration of **4** was determined to be 4*S*,5*R*,7*S*,8*R*,11*S* based on the experimental ECD spectrum, which was highly similar to the calculated ECD spectrum (**Figure 5**). Thus, the absolute configuration of compound **4** was fully confirmed and given the trivial name phomene B.

Compound **5** was isolated as yellow crystals. Its molecular formula of C_15_H_26_O_2_ was deduced by the HRESIMS spectrum with a protonated ion peak discovered at *m*/*z* 239.2004 [M + H]^+^ (calcd for C_15_H_27_O_2_, 239.2006), indicating three degrees of hydrogen deficiency. The IR spectrum of **5** revealed an obvious absorption band at 3,315 cm^-1^, indicating the presence of a series of free hydroxyl functionalities. The ^1^H NMR data (**Table 3**) of 5 showed three singlet methyl groups (*δ*_H_ 1.24, 1.24, and 1.26), a hydroxymethyl moiety (*δ*_H_ 3.37, 3.54), and various kinds of saturated aliphatic protons ranging from *δ*_H_ 1.22 to 2.61. According to the ^13^C NMR (**Table 3**) and HSQC data of compound **5,** 15 carbon signals were resolved, namely, three methyl moieties (*δ*_C_ 22.2, 23.4, and 25.4), six methylene groups (*δ*_C_ 18.6, 21.3, 29.2, 37.6, 46.3, and 68.2), and three methine functionalities (*δ*_C_ 41.8, 49.3, and 53.1) together with three quaternary carbons (*δ*_C_ 36.6, 42.3, and 74.0). With careful consideration of the molecular formula of **5**, this informative data strongly indicated that compound **5** might be a tricyclic sesquiterpenoid derivative.

**Figure 6:**
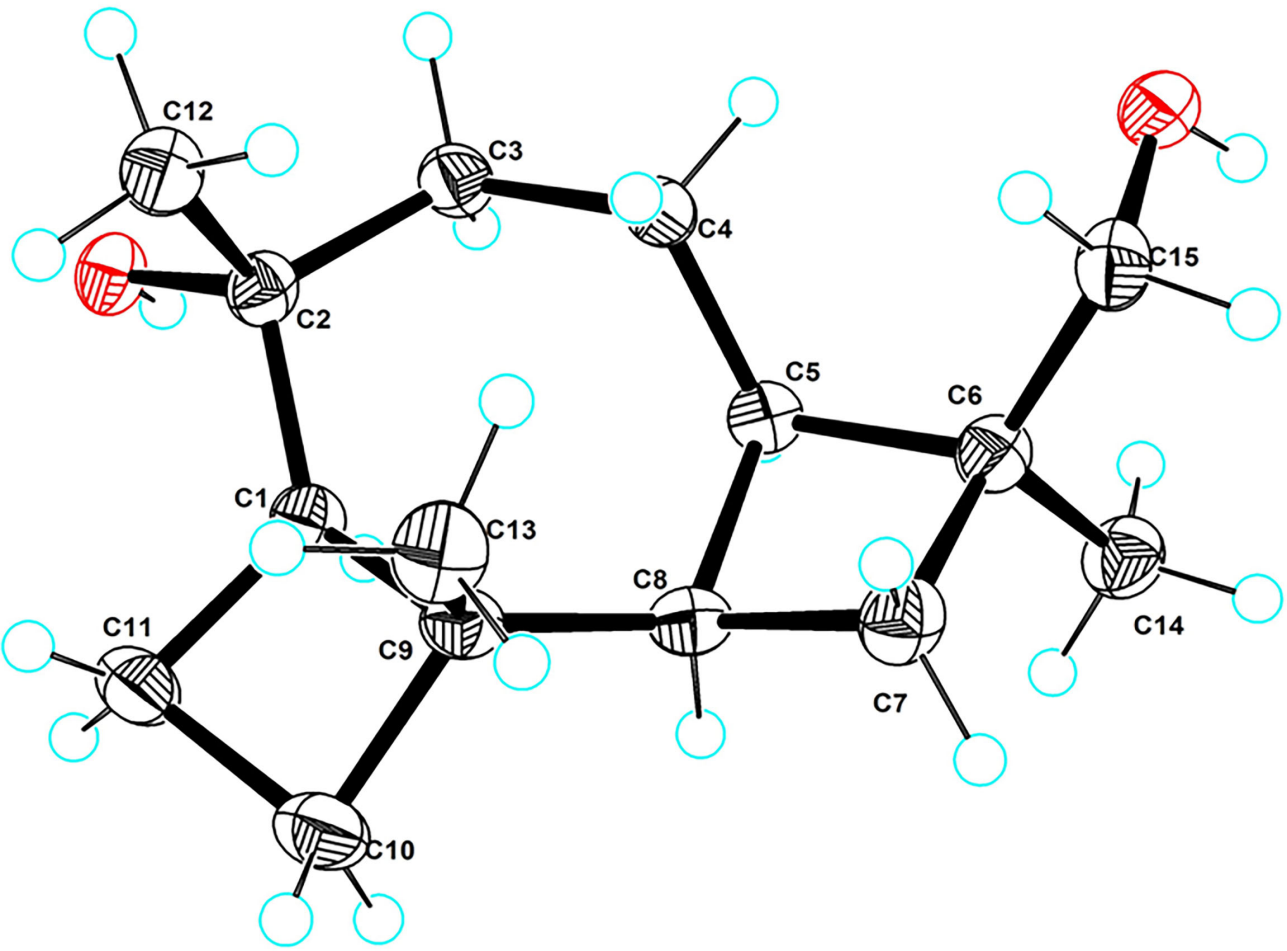
ORTEP drawing of the X-ray structure of **5**.

In the ^1^H–^1^H COSY spectrum, the obvious correlations of H-1/H_2_-11/H_2_-10 and H_2_-3/H_2_-4/H-5/H-8/H_2_-7 suggested the presence of two independent spin fragments **a** (C-1/C-11/C-10) and **b** (C-3/C-4/C-5/C-8/C-7). With reference to fragment **a**, the HMBC correlations from H_3_-13 to C-1 (*δ*_C_ 53.1), C-9 (*δ*_C_ 42.4), and C-10 (*δ*_C_ 37.6) evidently confirmed the presence of a cyclobutane ring (ring A). Meanwhile, the HMBC correlations from H_3_-14 to C-5 (*δ*_C_ 49.6), C-6 (*δ*_C_ 36.7), C-7 (*δ*_C_ 29.2), and C-15 (*δ*_C_ 68.2), H_2_-15 to C-5, C-6, and C-7 coupling with the COSY fragment C-5/C-6/C-7 further established the other cyclobutane ring (ring C) with a hydroxymethyl functionality attached at the C-6 position. The seven-membered ring B could be conveniently constructed by the HMBC correlations from H-1 to C-3 (*δ*_C_ 21.5) and C-8 (*δ*_C_ 41.9), H-3 to C-5, H-5 to C-9, H_3_-12 to C-1, C-2 (*δ*_C_ 74.0), and C-3 on the basis of the COSY fragment C-3/C-4/C-5/C-8. Lastly, the key HMBC correlations of H_2_-11 to C-2, H-7 to C-9, and H_2_-4 to C-6 could further establish the connection of the 4/7/4 fused ring system (rings A–C). Therefore, the planar structure of **5** was then determined. The relative and absolute configurations of compound **5** were unambiguously confirmed by the X-ray single crystal diffraction on the CuK*α* with a Flack parameter of -0.05 (12). Finally, the absolute configuration was thus designated as 1*R*,2*R*,5*R*,6*S*,8*R*,9*R*, as shown in **Figure 6**, and given the trivial name phomene C.

At this stage, compounds **1–6** were evaluated for antimicrobial activities against the bacteria *Escherichia coli*, *S. aureus*, and methicillin-resistant *S. aureus* (MRSA) (**Table 4**). The biological screening results showed that the new compound **2** showed very potent antimicrobial activities to Gram-positive stain *S. aureus* and MRSA with a minimum inhibitory concentration (MIC) value at 6.25 μg/ml for both. In addition, the known compound **6** also exhibited significant antimicrobial activities, with a MIC value of 1.56 μg/ml towards *S. aureus* and 0.78 μg/ml towards MRSA, respectively, which were very close to those of the positive control vancomycin (0.78 μg/ml for *S. aureus* and 0.78 μg/ml for MRSA). However, we have only tested the bacteriostatic potential of the compounds, and their bactericidal activity is still unknown and deserves further extensive exploration.

**Table 4:** Antibacterial activity of compounds 1–6 (minimum inhibitory concentration, μg/ml).

Compounds	*Escherichia coli*	*Staphylococcus aureus*	Methicillin-resistant *S. aureus*
Vancomycin/kanamycin	3.12	0.78	0.78
**1**	>100	100	100
**2**	>100	6.25	6.25
**3**	>100	100	100
**4**	>100	100	100
**5**	>100	100	100
**6**	>100	1.56	0.78

Furthermore, compounds **1–6** were also tested for their cytotoxicity against a panel of human cancer cell lines including SF-268, MCF-7, HepG-2, and A549 and normal cell line LX-2 (**Table 5**). However, all the tested compounds were found to be devoid of antitumor activity even at the concentration of 100 µM. The aforementioned biological screening results collectively pointed that the acetal meroterpenoids **2** and **6** might be severed as promising lead compounds towards antibacterial innovative drug development.

**Table 5:** Cytotoxicity activity of compounds 1–6 (IC_50_, μM).

Compounds	SF-268	MCF-7	HepG-2	A549	LX-2
Adriamycin	1.24 ± 0.01	1.08 ± 0.04	1.06 ± 0.05	1.10 ± 0.07	1.22 ± 0.03
**1**	115.93 ± 2.55	116.09 ± 1.98	120.72 ± 1.59	>128	91.45± 3.19
**2**	31.82 ± 1.72	57.22 ± 1.03	65.55 ± 3.11	42.34 ± 2.07	27.12 ± 2.53
**3**	118.08 ± 1.49	121.05 ± 2.16	>128	>128	93.27 ± 1.31
**4**	>128	>128	>128	>128	>128
**5**	>128	>128	>128	>128	> 128
**6**	46.48 ± 3.03	61.52 ± 1.79	39.53 ± 4.00	50.44 ± 6.20	21.08 ± 0.74

Conclusively, numerous excellent efforts towards the secondary metabolites of the plant endophytic fungi have successfully clarified that the endophytic fungi shared the outstanding ability to produce pharmaceutically meaningful natural products (Debbab et al., 2013; Mousa and Raizada, 2013; Brader et al., 2014) or similar bioactive metabolites as their hosts (Stierle et al., 1993; Kusari et al., 2011; Kusari et al., 2012). In recent years, the extraction and the isolation of bioactive leading natural products, with aim of discovering innovative drugs from the plant endophytic fungi, are emerging as a hot research topic for both natural product and medicinal chemists (Praptiwi et al., 2018; Tanapichatsakul et al., 2018; Adeleke and Babalola, 2020). However, the endophytic fungi of the *Alpinia shengzhen* plant have not been reported. This study reported the isolation and identification of the endophytic fungi of *Alpinia shengzhen* for the first time (Hu et al., 2011). As a result, six strains of endophytic fungi, namely, *Cladorrhinum* sp. SZSJ-2 (Madrid et al., 2011), *Phyllosticta capitalensis* SZSJ-3 (Arafat, 2018), *Nigrospora oryzae* SZSJ-5 (Hudson, 1963), and two strains of *Phomopsis* sp. SZSJ-7B and 7C (Zhang et al., 2000) together with *Annulohypoxylon stygium* SZSJ-7A (Hsieh et al., 2005), were isolated from the plant tissues of *Alpinia shengzhen* by section culture because of their abundant secondary metabolites and notable biological activities. These results informatively suggested that *Alpinia shengzhen* could be applied as a promising bioresource for the discovery of medicinal fungi.

In this study, a chemical investigation of the endophytic fungus *Phomopsis* sp. SZSJ-7B of *Alpinia shengzhen* was performed for the first time to discover novel lead drug molecules with chemically diverse structures and biologically significant activities. Although previous studies revealed a huge number of intriguing novel natural products isolated from the genus *Phomopsis* (Silva et al., 2006; Yang et al., 2013; Fan et al., 2020; Gong et al., 2020; Yang et al., 2020), five novel secondary metabolites and one known natural product were also successfully isolated from this genus in this time. The six natural products shared four different structure types, all of which were isolated from this genus for the first time, thus greatly enriching the structural types of natural compounds from *Phomopsis* sp. (Udayanga et al., 2011; Xu et al., 2021). Moreover, this chemical research effort can also strongly provide chemo-logical reference and experimental guidance for future studies towards the endophytic fungi and the secondary metabolites of other ginger plants.

The acetal skeleton represents a ubiquitous and intriguing family of structurally special architecture, which is prevalent in numerous biologically meaningful natural products (Pettit et al., 2015) and many pharmaceutically significant clinical drugs, such as aquamox for hypertension (Verdel et al., 2006) and cevimeline for parasympathetic nerves (Mavragani and Moutsopoulos, 2007). In this study, phomolides A and B were characterized with a natural, rarely occurring ester-acetal skeleton, which is constructed by a carboxylic acid and a phenol hydroxyl functionality (Chen et al., 2021) together with an aldehyde fragment (Ahmed Laskar and Younus, 2019), giving rise to a distinctive difference with the common acetal moiety formatted with two alcoholic hydroxyl groups and an aldehyde. To our knowledge, there were only four examples of natural products sharing this skeleton reported in previous literatures (Zhang et al., 2020; Choi et al., 2021). The discovery of phomolides A and B further enriches the structural types and the members of these ester-acetal compounds. Moreover, the biological evaluations showed that phomolide B exhibited a significant inhibitory activity against *S. aureus* and MRSA, which suggested that these ester-acetal compounds might continuously serve as potent innovative impetus for the further extensive research and medicinal exploration towards the development of novel antibacterial drugs (Rossiter et al., 2017; Wu et al., 2019; Dandawate et al., 2019; Dai et al., 2020).

Phomenes A and B possess a typical eremophilane-type sesquiterpene skeleton with a fascinating 6/6/5 fused ring system. The first example of this eremophilane sesquiterpene, named cyclodebney, was reported in 1986, which was successfully isolated from tobacco necrosis virus (TNV) *Niwtiutua dcbneyi* and then illustrated to show a potent antifungal activity. Furthermore, Le isolated three other new eremophilane-type sesquiterpenes from *Sarcographa tricosa* (Le et al., 2013) as characteristic secondary metabolites. Until now, there were about seven eremophilane sesquiterpenes with this 6/6/5 fused ring system isolated from medicinal plants (Wang et al., 2016; Shao et al., 2016) and fungi (Chang et al., 2017). However, phomenes A and B are the first two examples of eremophilane-type sesquiterpenes isolated from *Phomopsis* sp., and they further increase the structural diversity of secondary metabolites in this genus.

Phomene C is a tricyclic sesquiterpenoid derivative with a very intriguing 4/7/4 fused ring scaffold, which is a fascinating type of sesquiterpenoid skeleton rather rarely occurring in nature. Up to now, only two examples of this sesquiterpenoid, named koraiol and frabenol, have been previously isolated from the oleoresin of *Pinus koraiensis* and *Fimetariella rabenhorstii*, respectively (Khan et al., 1979; Tao et al., 2011). Moreover, the sesquiterpene alcohol 5,8-cyclocaryophyllan-4-ol, which was detected in Cangerana oil (Weyerstahl et al., 1996), also shared a closely similar carbon skeleton as that of koraiol and frabenol with a 4/7/4 fused ring system. In this study, the discovery of the new sesquiterpenoid phomene C further broadened the structural diversity and enriched the family members of this type of sesquiterpenoid.

## 3 Conclusion

In conclusion, this study firstly conducted a systematic chemical investigation on the secondary metabolites of the endophytic fungi *Phomopsis* sp. SZSJ-7B from *Alpinia shengzhen* and successfully resulted in the isolation of two novel acetal lactones (phomolides A and B), three undescribed sesquiterpenes (phomenes A–C), and a known lactone (colletotricholide A). All of these types of compounds were also isolated from *Phomopsis* sp. for the first time, which greatly enriched the structural diversity of the secondary metabolites of the genus. The structures of the new compounds were fully characterized by a combination of spectroscopic methods, X-ray diffraction, and quantum chemistry calculations. The biological activity screening clarified that both compounds **2** and **6** exhibited significant antibacterial activities towards MRSA and *S. aureus* strains with MIC values as low as 6.25 μg/ml, which were comparable to those of the positive control vancomycin without any notable cytotoxicity, thus illustrating its significant potential in the development of innovative antibacterial drugs. Moreover, further investigations on structure–activity relationship and antibacterial mechanism directed toward this goal are currently underway and will be reported in due course.

## 4 Experimental

### 4.1 General experimental procedures

IR data were measured on a Shimadzu IR Affinity-1 spectrometer (Shimadzu, Kyoto, Japan). UV and optical rotation data were obtained by a Shimadzu UV-2600 spectrophotometer (Shimadzu, Kyoto, Japan) and an Anton Paar MCP-500 spectropolarimeter (Anton Paar, Graz, Austria). The ECD spectra were measured with Applied Photophysis Chirascan. The NMR spectra (1D and 2D) data were collected on a Bruker Avance-500 spectrometer with tetramethylsilane as an internal standard (Bruker, Fällanden, Switzerland). The HRESIMS spectra were acquired with a Thermo MAT95XP high-resolution mass spectrometer (Thermo Fisher Scientific, Bremen, Germany). The single crystal data were collected on an Agilent Xcalibur Novasingle-crystal diffractometer equipped with CuK*α* radiation. A Hitachi Primaide [Hitachi Instruments (Dalian) Co., Ltd.] equipped with a diode array detector using a preparative YMC ODS C_18_ column (20 × 250 mm, 5 μm) was used for preparative HPLC separation. Sephadex LH-20 (GE Healthcare, Uppsala, Sweden), silica gel (200–300 and 60–100 mesh, Puke., Qingdao, China), and C_18_ reversed-phase silica gel (40-75 μm, Fuji, Kasugai, Japan) were used for column chromatography. All solvents were of analytical grade (Guangzhou Chemical Regents Company, Ltd., Guangzhou, China).

### 4.2 Fungal material

The fungal strain *Phomopsis* sp. SZSJ-7B was isolated from the fresh leaves of *Alpinia shengzhen* collected in the South China Botanical Garden in Guangzhou City, Guangdong Province of China in September 2020. Using BLAST to search the GenBank database, SZSJ-7B (GenBank accession number: OP623444.1) has 100% similarity with *Phomopsis* sp. MJ53 (GenBank accession number: KM203620.1). The strain is preserved at the Key Laboratory of South China Agricultural Plant Molecular Analysis and Genetic Improvement, South China Botanical Garden in Guangzhou City.

### 4.3 Fermentation, extraction, and isolation

The prepared fresh mycelium of the strain was inoculated into each of five 500-ml Erlenmeyer flasks containing 200 ml PDB medium (200 g potato, 20 g dextrose, 3 g KH_2_PO_4_, 1.5 g MgSO_4_, and 10 mg vitamin B in 1 L H_2_O) and then incubated at 28°C on a rotary shaker at 180 rpm for 5 days to obtain the seed culture. Fermentation was performed in 30 3-L Fernbach flasks, each containing 1.5 L PDB medium. After having been disinfected at 121°C for 30 min in an autoclave and cooled to room temperature, each flask was inoculated with 30 ml of the seed cultures and incubated at 28°C for 30 days. After cultivation, the mycelia were extracted with EtOAc for three times, and the crude extract (10 g) was obtained. The crude extract was subjected to silica gel using gradient elution with petroleum ether– EtOAc–methanol (MeOH) (v/v/v, 50:1:0→0:10:1) to afford six main fractions (Fr.1–Fr.6).

Fr.1 (880 mg) was isolated on silica gel and eluted with ether–EtOAc gradient (v/v, 100:0→2:1) to obtain six sub-fractions (Fr.1-1 to Fr.1-6). Fr.1-5 (154 mg) was eluted isocratically with ether–EtOAc (10:1) to afford compound **6** (5 mg).

Fr.2 (697 mg) was isolated on silica gel and eluted with ether–EtOAc gradient (v/v, 100:0→1:1) to obtain seven sub-fractions (Fr.2-1 to Fr.2-7). Fr.2-6 (217 mg) was isolated on silica gel and eluted with ether–EtOAc gradient (v/v, 20:1→2:1) to obtain five sub-fractions (Fr.2-6-1 to Fr.2-6-5). Fr.2-6-3 (111 mg) was isolated on silica gel and eluted with ether–CH_3_Cl gradient (v/v, 5:1→2:1) to obtain four sub-fractions (Fr.2-6-3-1 to Fr.2-6-3-4). Fr.2-6-3-4 (20 mg) was isolated on silica gel and eluted with ether–EtOAc gradient (v/v, 15:1→5:1) to obtain two sub-fractions (Fr.2-6-3-4-1 to Fr.2-6-3-4-2). Fr.2-6-3-4-2 (10 mg) was further purified by the preparative HPLC system with CH_3_CN–H_2_O (80:20) as eluent to afford compound **1** (3.8 mg, *t*_R_ = 7.0 min) and compound **2** (2.8 mg, *t*_R_ = 8.0 min).

Fr.3 (1.8 g) was separated by Sephadex LH-20 CC eluted with CHCl_3_–MeOH (v/v, 1:3) to afford three sub-fractions (Fr.3-1 to Fr.3-3). Fr.3-2 (205 mg) was isolated on silica gel and eluted with ether–EtOAc gradient (v/v, 100:0→1:1) to obtain seven sub-fractions (Fr.3-2-1 to Fr.3-2-7). Fr.3-2-6 (28.4 mg) was isolated on silica gel and eluted with ether–CHCl_3_ gradient (v/v, 10:1→1:1) to obtain compound **4** (3.6 mg).

Fr.4 (857 mg) was separated into nine subfractions (Fr.4-1 to Fr.4-9) on octadecyl-silylated silica gel column chromatography (ODS CC) with MeOH–H_2_O (v/v, 50:50→100:0). Fr.4-4 (109 mg) was isolated on silica gel and eluted with ether–EtOAc gradient (v/v, 100:0→1:1) to obtain compound **3** (3.4 mg).

Fr.5 (322 mg) was separated into five subfractions (Fr.5-1 to Fr.5-5) on ODS CC with MeOH–H_2_O (v/v, 30:70→100:0). Fr.5-3 (13.8 mg) was isolated on silica gel and eluted with ether–EtOAc gradient (v/v, 5:1→0:1) to obtain three sub-fractions (Fr.5-3-1 to Fr.5-3-3). Fr.5-3-2 (10.0 mg) was eluted isocratically with CHCl_3_–MeOH (50:1) to afford compound **5** (4.8 mg).

Phomolide A: white amorphous powder; ${\left[\alpha \right]}_{\text{D}}^{25}$ + 0.07 (*c* 0.1, MeOH); UV (MeOH) *λ*_max_ (log *ϵ*): 214 (3.38), 242 (2.56), 243 (2.73), and 282 (2.97) nm; IR (KBr): 3,325, 2,943, 2,833, 1,662, 1,448, 1,022, 970, and 667 cm^-1^; HRESIMS: *m*/*z* 209.0815 [M + H]^+^ (calcd for C_11_H_13_O_4_, 209.0808); ^1^H (500 MHz) and ^13^C (125 MHz) NMR data (see **Table 1**).

Phomolide B: white amorphous powder; ${\left[\alpha \right]}_{\text{D}}^{25}$ + 4.16 (*c* 0.1, MeOH); UV (MeOH) *λ*_max_ (log *ϵ*): 240 (2.78) and 266 (3.30) nm; IR (KBr): 3,360, 2,933, 2,833, 1,714, 1,680, 1,585, 1,456, 1,166, 1,020, and 669 cm^-1^; HRESIMS: *m*/*z* 385.2007 [M + H]^+^ (calcd for C_23_H_29_O_5_, 385.2010); ^1^H (500 MHz) and ^13^C (125 MHz) NMR data (see **Table 1**).

Phomene A: yellow oil; ${\left[\alpha \right]}_{\text{D}}^{25}$ – 2.17 (*c* 0.1, MeOH); UV (MeOH) *λ*_max_ (log *ϵ*): 200 (3.30) nm; IR (KBr): 3,363, 2,927, 2,858, 1,739, 1,653, 1,516, 1,454, 1,377, 1,251, 1,024, 923, 675, and 597 cm^-1^; HRESIMS: *m*/*z* 237.1851 [M + H]^+^ (calcd for C_15_H_25_O_2_, 237.1849); ^1^H (500 MHz) and ^13^C (125 MHz) NMR data (see **Table 3**).

Phomene B: yellow oil; ${\left[\alpha \right]}_{\text{D}}^{25}$ – 4.21 (*c* 0.1, MeOH); UV (MeOH): *λ*_max_ (log *ϵ*): 200 (3.25) nm; IR (KBr): 3,361, 2,929, 2,858, 1,739, 1,653, 1,541, 1,516, 1,454, 1,379, 1,024, 952, 667, and 597 cm^-1^; HRESIMS: *m*/*z* 237.1854 [M + H]^+^ (calcd for C_15_H_25_O_2_, 237.1849); ^1^H (500 MHz) and ^13^C (125 MHz) NMR data (see **Table 3**).

Phomene C: yellow crystal; ${\left[\alpha \right]}_{\text{D}}^{25}$ + 0.063 (*c* 0.1, MeOH); UV (MeOH): *λ*_max_ (log *ϵ*): 223 (1.60) and 237 (1.67) nm; IR (KBr): 3,315, 2,947, 2,858, 1,651, 1,375, 1,112, 1,029, 912, 665, and 603 cm^-1^; HRESIMS: *m*/*z* 239.2004 [M + H]^+^ (calcd for C_15_H_27_O_2_, 239.2006); ^1^H (500 MHz) and ^13^C (125 MHz) NMR data (see **Table 3**).

### 4.4 Quantum chemistry calculations

Conformational search of structures was performed by Crest (Pracht et al., 2020), with 4 kcal/mol energy window. Optimization and frequency calculation of the obtained conformer were performed on B3LYP/TZVP (Grimme et al., 2011; Tsuzuki and Uchimaru, 2020) (IEFPCM, CDCl_3_ and MeOH) level of theory. DFT GIAO ^13^C NMR calculation was calculated on the ωB97xD/6-31G* (IEFPCM, CDCl_3_) level, and the data processing followed the reported STS protocol. The calculated shielding tensors of conformers were Boltzmann-averaged based on Gibbs free energy. Theoretical ECD (TDDFT) calculation was calculated on mPW1PW91/TZVP (IEFPCM, MeOH) level. SpecDis v1.71 was used to simulate the ECD curve with sigma/gamma value of 0.35 eV (Bruhn et al., 2013). The calculated ECD curve of each conformer was Boltzmann-averaged based on their Gibbs free energy. The average calculated ECD curve of **1** was adjusted by blue shifting for 20 nm. All DFT calculations were performed by Gaussian 16 software package (Frisch et al., 2016).

### 4.5 X-ray crystallographic data

Crystal data for **6** C_15_H_26_O_2_ (*M* = 238.36 g/mol): trigonal, space group P3_2_ (no. 145), *a* = 13.6894 (2) Å, *c* = 6.60290 (10) Å, *V* = 1,071.60 (4) Å^3^, *Z* = 3, *T* = 100.00 (10) K, *μ*(CuK*α*) = 0.552 mm^-1^, Dcalc = 1.108 g/cm^3^; 7,072 reflections measured (7.456 ≤ 2Θ ≤ 148.49) and 2,779 unique (*R*_int_ = 0.0298, *R*_sigma_ = 0.0367), which were used in all calculations. The final *R*_1_ was 0.0422 [I > 2*σ*(I)] and *wR*_2_ was 0.1085 (all data). Flack parameter = -0.05 (12). The crystallographic data for **5** reported in this paper has been deposited in the Cambridge Crystallographic Data Centre (deposition number: CCDC 2192981). Copies of these data can be obtained free of charge *via* https://www.ccdc.cam.ac.uk.

### 4.6 Cytotoxicity and antimicrobial assays

#### 4.6.1 Cytotoxicity assays

Cytotoxicity was evaluated by the sulforhodamine B assay (Skehan et al., 1990) against five human cancer cell lines (SF-268, MCF-7, HepG2, A549, and LX-2). As a result, none of the compounds showed good cytotoxic activity.

#### 4.6.2 Antimicrobial assays

The antibacterial activities for compounds **1**–**6** were evaluated against three bacteria embodying *S. aureus* (CMCC 26003), methicillin-resistant *S. aureus* (JCSC 3063), and *E. coli* (ATCC 8739). All of the bacteria were purchased from Guangdong Institute of Microbiology (Guangzhou, China). The MICs were determined by the broth microdilution method in 96-well plates as described in previous literature (NCCLS, 1999; CLSI, 2012; Li et al., 2014). Positive control was vancomycin or kanamycin. All test samples were dissolved in dimethyl sulfoxide and diluted with culture medium.

## Data availability statement

The data presented in the study are deposited in the Cambridge Crystallographic Data Centre, accession number: CCDC 2192981.

## Author contributions

YC, HW, and XK conducted the experiment and collected the experimental data. YC performed the experiments of compound isolation. ZS and ZZ carried out the ECD calculations. YC, ZZ, and HT finished the structure identification of the isolated compounds. MK, WP, JT, and YZ evaluated the activities of all the isolates. YC and HT interpreted the data and wrote the paper. YC, ZZ, and HT revised the manuscript. ZZ and HT conceived and designed the experiments. All authors contributed to the article and approved the submitted version.

## Funding

Financial support for this research was provided by the National Natural Science Foundation of China (82173711), Youth Innovation Promotion Association of CAS (2020342), Natural Science Foundation of Guangdong Province (2019A1515011694), Natural Science Foundation of Hunan Province (no. 2021JJ30917), High-tech Industry Science and Technology Innovation Project of Hunan Province (2020GK4083), Postgraduate Research and Innovation Project of Hunan Province (CX20210341), Postgraduates Innovation Program of Central South University (Nos. 2021zzts0978, 2021zzts0994, and 2022zzts0899), and the Open Sharing Fund for the Large-Scale Instruments and Equipment of Central South University.

## Acknowledgments

We sincerely thank Ms. Xuan Ma of South China Sea Institute of Oceanology for the X-ray measurements.

## Conflict of interest

The authors declare that the research was conducted in the absence of any commercial or financial relationships that could be construed as a potential conflict of interest.

## Publisher’s note

All claims expressed in this article are solely those of the authors and do not necessarily represent those of their affiliated organizations, or those of the publisher, the editors and the reviewers. Any product that may be evaluated in this article, or claim that may be made by its manufacturer, is not guaranteed or endorsed by the publisher.

## References

[B1] Adeleke B. S. Babalola O. O. (2020). Oilseed crop sunflower (*Helianthus annuus*) as a source of food: nutritional and health benefits. Food. Sci. Nutr. 8, 4666–4684. doi: 10.1002/fsn3.1783 32994929 PMC7500752

[B2] Ahmed Laskar A. Younus H. (2019). Aldehyde toxicity and metabolism: the role of aldehyde dehydrogenases in detoxification, drug resistance and carcinogenesis. Drug Metab. Rev. 51, 42–64. doi: 10.1080/03602532.2018.1555587 30514131

[B3] Arafat K. (2018). A novel isolate of *Phyllosticta capitalensis* causes black spot disease on guava fruit in Egypt. Asian. J. Plant Pathol. 12, 27–37. doi: 10.3923/ajppaj.2018.27.37

[B4] Brader G. Compant S. Mitter B. Trognitz F. Sessitsch A. (2014). Metabolic potential of endophytic bacteria. Curr. Opin. Biotechnol. 27, 30–37. doi: 10.1016/j.copbio.2013.09.012 24863894 PMC4045207

[B5] Bruhn T. Schaumlöffel A. Hemberger Y. Bringmann. G. (2013). SpecDis: quantifying the comparison of calculated and experimental electronic circular dichroism spectra. Chirality 25, 243–249. doi: 10.1002/chir.22138 23532998

[B6] Bunyapaiboonsri T. Yoiprommarat S. Srikitikulchai P. Srichomthong K. Lumyong S. (2010). Oblongolides from the endophytic fungus phomopsis sp. BCC 9789. J. Nat. Prod. 73, 55–59. doi: 10.1021/np900650c 20038128

[B7] Burden R. S. Loeffler R. S. T. Rowell P. M. Bailey J. A. Kemp M. S. (1986). Cyclodebneyol, a fungi toxic sesquiterpene from TNV infected *Nicotiana debneyi* . Phytochem 25, 1607–1608. doi: 10.1016/s0031-9422(00)81217-0

[B8] Cai R. L. Chen S. H. Liu Z. M. Tan C. B. (2017). A new alpha-pyrone from the mangrove endophytic fungus phomopsis sp. HNY29-2B. Nat. Prod. Res. 31, 124–130. doi: 10.1080/14786419.2016.1214833 27687677

[B9] Chai J. D. Head-Gordon M. (2008). Long-range corrected hybrid density functionals with damped atom-atom dispersion corrections. Phys. Chem. Chem. Phys. 10, 6615–6620. doi: 10.1039/B810189B 18989472

[B10] Chang J. C. Hsiao G. Lin R. K. Kuo Y. H. Ju Y. M. Lee T. H. (2017). Bioactive constituents from the termite nest-derived medicinal fungus *Xylaria nigripes* . J. Nat. Prod. 80, 38–44. doi: 10.1021/acs.jnatprod.6b00249 28055210

[B11] Chen H. P. Huang M. X. Li X. W. Chen B. Wang J. Lin Y. C. . (2018). Phochrodines a–d, first naturally occurring new chromenopyridines from mangrove entophytic fungus phomopsis sp. 33. *Fitoterapia* 124, 103–107. doi: 10.1016/j.fitote.2017.10.013 29074224

[B12] Chen S. C. Liu Z. M. Tan H. B. Chen Y. C. Liu H. X. Zhang W. M. . (2019). Tersone a-G, new pyridone alkaloids from the deep-sea fungus *Phomopsis tersa* . Mar. Drugs 17, 394–407. doi: 10.3390/md17070394 31277263 PMC6669727

[B13] Chen S. C. Liu Z. M. Tan H. B. Zhu S. Liu H. X. Zhang W. M. . (2020). Photeroids a and b, unique phenol-sesquiterpene meroterpenoids from the deep-sea-derived fungus phomopsis tersa. Org. Biomol. Chem. 18, 642–645. doi: 10.1039/c9ob02625h 31916553

[B14] Chen X. Li D. Zhang H. Duan Y. Huang Y. (2021). Sinomenine-phenolic acid coamorphous drug systems: solubilization, sustained release, and improved physical stability. Int. J. Pharm. 598, 120389. doi: 10.1016/j.ijpharm.2021.120389 33609724

[B15] Choi B. K. Cho D. Y. Choi D. K. Shin H. J. (2021). Miharadienes a-d with unique cyclic skeletons from a marine-derived streptomyces miharaensis. Org. Chem. Front. 8, 4845–4852. doi: 10.1039/D1QO00773D

[B16] CLSI (2012). Methods for dilution antimicrobial susceptibility tests for bacteria that grow aerobically. 7th edition (Wayne, PA: Clinical and Laboratory Standards Institute).

[B17] Dai J. Han R. Xu Y. Li N. Wang J. Dan W. (2020). Recent progress of antibacterial natural products: future antibiotics candidates. Bioorg. Chem. 101, 103922. doi: 10.1016/j.bioorg.2020.103922 32559577

[B18] Dandawate P. Padhye S. Schobert R. Biersack B. (2019). Discovery of natural products with metal-binding properties as promising antibacterial agents. Expert. Opin. Drug Discovery 14, 563–576. doi: 10.1080/17460441.2019.1593367 30905202

[B19] Debbab A. Aly A. H. Proksch P. (2013). Mangrove derived fungal endophytes – a chemical and biological perception. Fungal. Divers. 61, 1–27. doi: 10.1007/s13225-013-0243-8

[B20] Ding B. Yuan J. Huang X. S. He L. Tan H. M. She Z. G. . (2013). New dimeric members of the phomoxanthone family: phomolactonexanthones a, b and deacetylphomoxanthone c isolated from the fungus phomopsis sp. Mar. Drugs 11, 4961–4972. doi: 10.3390/md11124961 24335522 PMC3877896

[B21] Ditchfield R. (1972). Molecular orbital theory of magnetic shielding and magnetic susceptibility. J. Chem. Phys. 56, 5688. doi: 10.1063/1.1677088

[B22] Fan M. M. Xiang G. Chen J. W. Zhou L. Jiao R. H. Shen Y. . (2020). Libertellenone m, a diterpene derived from an endophytic fungus phomopsis sp. S12, protects against DSS-induced colitis *via* inhibiting both nuclear translocation of NF-κB and NLRP3 inflammasome activation. Int. Immunopharmacol. 80, 106144. doi: 10.1016/j.intimp.2019.106144 31927507

[B23] Frisch M. J. Trucks G. W. Schlegel H. B. Scuseria G. E. Robb M. A. Cheeseman J. R. (2016). Gaussian 16, revision C.01 (Wallingford CT: Gaussian, Inc.).

[B24] Gong J. L. Lu Y. Wu W. H. Xi J. G. Tang S. B. Yi K. X. . (2020). First report of *Phomopsis heveicola* (anamorph of *Diaporthe tulliensis*) causing leaf blight of *Coffee* (*Coffea arabica*) in China. Plant Dis. 104, 570–571. doi: 10.1094/PDIS-09-19-1833-PDN

[B25] Gouda S. Das G. Sen S. K. Shin H. S. Patra J. K. (2016). Endophytes: a treasure house of bioactive compounds of medicinal importance. Front. Microbiol. 7. doi: 10.3389/fmicb.2016.01538 PMC5041141 27746767

[B26] Grimme S. Ehrlich S. Goerigk L. (2011). Effect of the damping function in dispersion corrected density functional theory. J. Energy Chem. 32, 1456–1465. doi: 10.1002/jcc.21759 21370243

[B27] Hemtasin C. Kanokmedhakul S. Kanokmedhakul K. Soytong K. Prabpai S. Kongsaeree P. . (2011). Cytotoxic pentacyclic and tetracyclic aromatic sesquiterpenes from *Phomopsis archeri* . J. Nat. Prod. 74, 609–613. doi: 10.1021/np100632g 21341709

[B28] Hsieh H. M. Ju Y. M. Rogers J. D. (2005). Molecular phylogeny of hypoxylon and closely related genera. Mycologia 97, 844–865. doi: 10.3852/mycologia.97.4.844 16457354

[B29] Huang Z. J. Cai X. L. Shao C. S. Yang J. X. Zhou S. N. Lin Y. C. . (2008). Chemistry and weak antimicrobial activities of phomopsins produced by mangrove endophytic fungus phomopsis sp. ZSU-H76. Phytochem 69, 1604–1608. doi: 10.1016/j.phytochem.2008.02.002 18343465

[B30] Huang R. Jiang B. G. Li X. N. Zheng K. X. He J. Wu S. H. . (2018). Polyoxygenated cyclohexe-noids with promising alpha-glycosidase inhibitory activity produced by phomopsis sp. YE3250, an endophytic fungus derived from *Paeonia delavayi* . J. Agr. Food. Chem. 66, 1140–1146. doi: 10.1021/acs.jafc.7b04998 29334729

[B31] Hudson H. J. (1963). The perfect state of *Nigrospora oryzae* . British. Mycological. Soc. 46, 355–360. doi: 10.1016/S0007-1536(63)80027-3

[B32] Hussain H. Krohn K. Ahmed I. Schulz B. Di Pietro S. Pescitelli G. . (2012). Phomopsinones a–d: four new pyrenocines from endophytic fungus phomopsis sp. Eur. J. Org. Chem. 2012, 1783–1789. doi: 10.1002/ejoc.201101788

[B33] Hu Z. X. Wu Y. Xie S. S. Luo Z. W. Xue Y. B. Zhang Y. H. . (2017). Phomopsterones a and b, two functionalized ergostane-type steroids from the endophytic fungus phomopsis sp. TJ507A. Org. Lett. 19, 258–261. doi: 10.1021/acs.orglett.6b03557 28004944

[B34] Hu J. Z. Ye Y. S. Zou P. Liao J. P. (2011). Studies on the hybrid breeding and biological characteristics of zingiberaceous plant (*Alpinia hainanensis* ‘Shengzhen’). J. Trop. Subtrop. Bot. 19, 279–282. doi: 10.969/j.issn.1005-3395.2011.03.014

[B35] Jouda J. B. Tamokou J. D. Mbazoa C. D. Douala-Meli C. Sarkar P. Bag P. K. . (2016). Antibacterial and cytotoxic cytochalasins from the endophytic fungus phomopsis sp. harbored in garcinia kola (Heckel) nut. BMC. Complement. Altern. Med. 16, 462–462. doi: 10.1186/s12906-016-1454-9 27842536 PMC5109658

[B36] Khan V. A. Gatilov Y. V. Dubovenko Z. V. Pentegova V. A. (1979). Crystal structure of koraiol-a sesquiterpene alcohol with a new type of carbon skeleton from the oleoresin of *Pinus koraiensis* . Chem. Nat. Compd. 15, 572–576. doi: 10.1007/bf00565927

[B37] Krohn K. Farooq U. Hussain H. Draeger S. Schulz B. van Ree T. . (2011). Phomosines h-J, novel highly substituted biaryl ethers, isolated from the endophytic fungus phomopsis sp. from *Ligustrum vulgare* . Nat. Prod. Commun. 6, 1907–1912. doi: 10.1177/1934578X1100601229 22312736

[B38] Kusari S. Hertweck C. Spiteller M. (2012). Chemical ecology of endophytic fungi: origins of secondary metabolites. Chem. Biol. 19, 792–798. doi: 10.1016/j.chembiol.2012.06.004 22840767

[B39] Kusari S. Zühlke S. Spiteller M. (2011). Effect of artificial reconstitution of the interaction between the plant *Camptotheca acuminata* and the fungal endophyte *Fusarium solani* on camptothecin biosynthesis. J. Nat. Prod. 74, 764–775. doi: 10.1021/np1008398 21348469

[B40] Le D. H. Takenaka Y. Hamada N. Tanahashi T. (2013). Eremophilane-type sesquiterpenes from cultured lichen mycobionts of *Sarcographa tricosa* . Phytoche 91, 242–248. doi: 10.1016/j.phytochem.2012.01.009 22285621

[B41] Li J. Liu J. K. Wang W. X. (2020). GIAO ^13^C NMR calculation with sorted training sets improves accuracy and reliability for structural assignation. J. Org. Chem. 85, 11350–11358. doi: 10.1021/acs.joc.0c01451 32786639

[B42] Li L. Y. Sattler I. Deng Z. W. Peschel G. Grabley S. Lin W. H. . (2008). A-seco-oleane-type triterpenes from phomopsis sp. (strain HKI0458) isolated from the mangrove plant *Hibiscus tiliaceus* . Phytochem 69, 511–517. doi: 10.1016/j.phytochem.2007.08.010 17889046

[B43] Liu H. B. Liu Z. M. Chen Y. C. Li D. L. Liu H. X. Zhang W. M. . (2021). Cytotoxic diaporindene and tenellone derivatives from the fungus *Phomopsis lithocarpus* . Chin. J. Nat. Med. 19, 874–880. doi: 10.1016/S1875-5364(21)60095-X 34844726

[B44] Li C. R. Zhai Q. Q. Wang X. K. Li G. Q. Zhang W. X. You X. F. . (2014). *In vivo* antibacterial activity of MRX-I, a new oxazolidinone. Antimicrob. Agents. Chemother. 58, 2418–2421. doi: 10.1128/aac.01526-13 24395231 PMC4023790

[B45] Madrid H. Cano J. Gené J. Guarro J. (2011). Two new species of *Cladorrhinum* . Mycologia 103, 795–805. doi: 10.3852/10-150 21307165

[B46] Mavragani C. P. Moutsopoulos H. M. (2007). Conventional therapy of sjogren's syndrome. Clin. Rev. Allergy Immunol. 32, 284–291. doi: 10.1007/s12016-007-8008-3 17992595

[B47] McWeeny R. (1961). Perturbation theory for the fock-dirac density matrix. Phys. Rev. 126, 1028–1034. doi: 10.1103/PhysRev.126.1028

[B48] Mousa W. K. Raizada M. N. (2013). The diversity of anti-microbial secondary metabolites produced by fungal endophytes: an interdisciplinary perspective. Front. Microbiol. 4. doi: 10.3389/fmicb.2013.00065 PMC3608919 23543048

[B49] NCCLS (1999). Methods for determining bactericidal activity of antimicrobial agents (Wayne, PA: Approved guideline M26-A National Committee for Clinical Laboratory Standards).

[B50] Pavao G. B. Vinícius V. P. de Oliveira A. L. Mara R. A. Lusânia M. G. A. Hosana M. D. . (2016). Differential genotoxicity and cytotoxicity of phomoxanthone a isolated from the fungus *Phomopsis longicolla* in HL60 cells and peripheral blood lymphocytes. Toxicol. In Vitro. 37, 211–217. doi: 10.1016/j.tiv.2016.08.010 27546515

[B51] Pettit G. R. Xu J. P. Chapuis J. C. Melody N. (2015). The cephalostatins. 24. isolation, structure, and cancer cell growth inhibition of cephalostatin 20. J. Nat. Prod. 78, 1446–1450. doi: 10.1021/acs.jnatprod.5b00129 26042639

[B52] Pracht P. Bohle F. Grimme S. (2020). Automated exploration of the low-energy chemical space with fast quantum chemical methods. Phys. Chem. Chem. Phys. 22, 7169–7192. doi: 10.1039/C9CP06869D 32073075

[B53] Praptiwi M. R. Wulansari D. Fathoni A. Agusta A. (2018). Antibacterial and antioxidant activities of endophytic fungi extracts of medicinal plants from *Central sulawesi* . J. Appl. Pharm. Sci. 8, 069–074. doi: 10.7324/JAPS.2018.8811

[B54] Rossiter S. E. Fletcher M. H. Wuest W. M. (2017). Natural products as platforms to overcome antibiotic resistance. Chem. Rev. 117, 12415–12474. doi: 10.1021/acs.chemrev.7b00283 28953368 PMC5869711

[B55] Shao H. Mei W. L. Kong F. D. Li W. Zhu G. P. Dai H. F. . (2016). Sesquiterpenes of agarwood from *Gyrinops salicifolia* . Fitoterapia 113, 182–187. doi: 10.1016/j.fitote.2016.07.015 27491753

[B56] Silva G. H. Teles H. L. Zanardi L. M. Costa-Neto C. M. Castro Gamboa I. Bolzani V. . (2006). Cadinane sesquiterpenoids of *Phomopsis cassiae*, an endophytic fungus associated with *Cassia spectabilis* (Leguminosae). Phytochem 67, 1964–1969. doi: 10.1016/j.phytochem.2006.06.004 16857221

[B57] Skehan P. Storeng R. Scudiero D. Monks A. Mcmahon J. Vistica D. . (1990). New colorimetric cytotoxicity assay for anticancer-drug screening. J. Natl. Cancer. Inst. 82, 1107–1112. doi: 10.1093/jnci/82.13.1107 2359136

[B58] Stierle A. Strobel G. Stierle D. (1993). Taxol and taxane production by taxomyces andreanae, an endophytic fungus of pacific yew. Science 260, 214–216. doi: 10.1126/science.8097061 8097061

[B59] Tanapichatsakul C. Monggoot S. Gentekaki E. Pripdeevech P. (2018). Antibacterial and antioxidant metabolites of diaporthe spp. isolated from flowers of *Melodorum fruticosum* . Curr. Microbiol. 7, 476–483. doi: 10.1007/s00284-017-1405-9 29159689

[B60] Tao M. H. Yan J. Wei X. Y. Li D. L. Zhang W. M. Tan J. W. (2011). A novel sesquiterpene alcohol from *Fimetariella rabenhorstii*, an endophytic fungus of *Aquilaria sinensis* . Nat. Prod. Commun. 6, 763–766. doi: 10.1002/mnfr.201100206 21815406

[B61] Tsuzuki S. Uchimaru T. (2020). Accuracy of intermolecular interaction energies, particularly those of hetero-atom containing molecules obtained by DFT calculations with grimme’s D2, D3 and D3BJ dispersion corrections. Phys. Chem. Chem. Phys. 22, 22508–22519. doi: 10.1039/D0CP03679J 33000847

[B62] Udayanga D. Liu X. McKenzie E. H. C. Chukeatirote E. Bahkali A. H. A. Hyde K. D. (2011). The genus *Phomopsis*: Biology, applications, species concepts and names of common phytopathogens. Fungal. Divers. 50, 189–225. doi: 10.1007/s13225-011-0126-9

[B63] Verdel B. M. Souverein P. C. Egberts A. C. Leufkens H. G. (2006). Difference in risks of allergic reaction to sulfonamide drugs based on chemical structure. Ann. Pharmacother. 40, 1040–1046. doi: 10.1016/j.cyto.2007.04.004 16735666

[B64] Wang H. N. Dong W. H. Huang S. Z. Wang J. Mei W. L. Dai H. F. . (2016). Three new sesquiterpenoids from agarwood of *Aquilaria crassna* . Fitoterapia 114, 7–11. doi: 10.1016/j.fitote.2016.07.014 27502285

[B65] Wei W. Gao J. Shen Y. Chu Y. L. Xu Q. Tan R. X. (2014). Immunosuppressive diterpenes from phomopsis sp. S12. Eur. J. Org. Chem. 2014, 5728–5734. doi: 10.1002/ejoc.201402491

[B66] Weyerstahl P. Schneider S. Marschall H. (1996). Constituents of the Brazilian cangerana oil. Flavour. Fragr. J. 11, 81–94. doi: 10.1002/(SICI)1099-1026

[B67] Wu S. C. Liu F. Zhu K. Shen J. Z. (2019). Natural products that target virulence factors in antibiotic-resistant *Staphylococcus aureus* . J. Agric. Food. Chem. 67, 13195–13211. doi: 10.1021/acs.jafc.9b05595 31702908

[B68] Xie S. S. Wu Y. Qiao Y. B. Guo Y. Wang J. P. Zhang Y. H. . (2018). Protoilludane, illudalane, and botryane sesquiterpenoids from the endophytic fungus phomopsis sp. TJ507A. J. Nat. Prod. 81, 1311–1320. doi: 10.1021/acs.jnatprod.7b00889 29771527

[B69] Xu J. L. Liu Z. M. Chen Y. C. Tan H. B. Liu H. X. Zhang W. M. . (2019a). Lithocarols a-f, six tenellone derivatives from the deep-sea derived fungus *Phomopsis lithocarpus* FS508. Bioorg. Chem 87, 728–735. doi: 10.1016/j.bioorg.2019.03.078 30954837

[B70] Xu T. C. Lu Y. H. Wang J. F. Liu S. S. Liu C. S. Wu S. H. . (2021). Bioactive secondary metabolites of the genus *Diaporthe* and anamorph *Phomopsis* from terrestrial and marine habitats and endophytes: 2010–2019. Microorganisms 9, 217. doi: 10.3390/microorganisms9020217 33494367 PMC7912663

[B71] Xu K. Zhang X. Chen J. W. Tan R. X. Jiao R. H. Ge H. M. . (2019b). Anti-inflammatory diterpenoids from an endophytic fungus phomopsis sp. S12. Tetrahedron. Lett. 60, 151045–151045. doi: 10.1016/j.tetlet.2019.151045

[B72] Yang H. Y. Gao Y. H. Niu D. Y. Gao X. M. Du G. Hu Q. F. . (2013). Xanthone derivatives from the fermentation products of an endophytic fungus phomopsis sp. Fitoterapia 91, 189–193. doi: 10.1016/j.fitote.2013.09.004 24042071

[B73] Yang Z. J. Zhang Y. F. Wu K. Jiang Z. T. Ge M. Shao L. . (2020). New azaphilones, phomopsones a-c with biological activities from an endophytic fungus phomopsis sp. CGMCC No.5416. Fitoterapia 145, 104573. doi: 10.1016/j.fitote.2020.104573 32222428

[B74] Yan B. C. Wang W. G. Hu D. B. Sun X. Kong L. M. Pu J. X. . (2016). Phomopchalasins a and b, two cytochalasans with polycyclic-fused skeletons from the endophytic fungus phomopsis sp. shj2. Org. Lett. 18, 1108–1111. doi: 10.1021/acs.orglett.6b00214 26881701

[B75] Yu B. Z. Zhang G. H. Du Z. Z. Zheng Y. T. Xu J. C. Luo X. D. (2008). Phomoeuphorbins a-d, azaphilones from the fungus *Phomopsis euphorbiae* . Phytochem 69, 2523–2526. doi: 10.1016/j.phytochem.2008.07.013 18799173

[B76] Zhang Z. Schwartz S. Wagner L. Miller W. (2000). A greedy algorithm for aligning DNA sequences. J. Comput. Biol. 7, 203–214. doi: 10.1089/10665270050081478 10890397

[B77] Zhang W. G. Wang M. M. Zhang S. Xu K. P. Tan H. B. (2020). Eutyscoparols a-G, polyketide derivatives from endophytic fungus *Eutypella scoparia* SCBG-8. Fitoterapia 146, 104681. doi: 10.1016/j.fitote.2020.104681 32628984

[B78] Zhao W. T. Liu Q. P. Chen H. Y. Zhao W. Gao Y. Yang X. L. (2020). Two novel eremophylane acetophenone conjugates from *Colletotrichum gloeosporioides*, an endophytic fungus in *Salvia miltiorrhiza* . Fitoterapia 141, 104474. doi: 10.1016/j.fitote.2020.104474 31927010

