# Supplementary material for: Five new secondary metabolites from an endophytic fungus Phomopsis sp. SZSJ-7B
Source: Front Plant Sci. 2022 Nov 14;13:1049015. doi: 10.3389/fpls.2022.1049015 (PMC9702824; doi:10.3389/fpls.2022.1049015)
Supplement: Supplementary file 1 [file DataSheet_1.pdf]

Five new secondary metabolites from an endophytic fungus *Phomopsis* sp. SZSJ-7B

Yan Chen[a,b,c], Huan Wang[c], Xin Ke[a,b,c], Zihuan Sang[a,b,c], Min Kuang[a,b], Weiwei Peng[a,b,c], Jianbing Tan[a,b], Yuting Zheng[a,b], Zhenxing Zou[a,b*], Haibo Tan[a,b,c*]

[a]Xiangya School of Pharmaceutical Sciences, Central South University, Changsha 410013, China;

[b]Hunan Key Laboratory of Diagnostic and Therapeutic Drug Research for Chronic Diseases, Central South University, Changsha 410013, China;

[c]Key Laboratory of South China Agricultural Plant Molecular Analysis and Genetic Improvement, Guangdong Provincial Key Laboratory of Applied Botany, South China Botanical Garden, Chinese Academy of Sciences, Guangzhou 510650, China

*** Correspondence:**
Corresponding Author
zouzhenxing@csu.edu.cn (Z.-X. Zou),
tanhaibo@scbg.ac.cn (H.-B. Tan)

Key words: Endophytic fungus; *Phomopsis*; Secondary metabolites; Antibacterial activity; Cytotoxic activity

Abstract:

Two undescribed lactones phomolides A-B (**1** and **2**) and three new sesquiterpenoids phomenes A-C (**3-5**), together with one known compound colletotricholide A (**6**) were isolated from the endophytic fungus *Phomopsis* sp. SZSJ-7B. Their chemical structures including the absolute configurations were comprehensively established by extensive analyses of NMR, HRESIMS, ECD powered by theoretical calculations, and X-ray diffractions. Moreover, the cytotoxic and antibacterial activities of compounds **1-6** were also evaluated, and the results demonstrated that compound **2** showed significant antibacterial effects towards methicillin-resistant *Staphylococcus aureus* (MRSA) and *S. aureus* strains with MIC low to 6.25 μg/mL, which was comparable to that of the clinical drug vancomycin. Moreover, all compounds had no cytotoxic activity.

Contents

Figure S1. ^{1}H NMR spectrum of **1** (CD_3OD, 500 MHz) 4
Figure S2. ^{13}C NMR spectrum of **1** (CD_3OD, 125 MHz) 4
Figure S3. ^{1}H-^{1}H COSY spectrum of **1** (CD_3OD) 5
Figure S4. HSQC spectrum of **1** (CD_3OD) 5
Figure S5. HMBC spectrum of **1** (CD_3OD) 6
Figure S6. NOESY spectrum of **1** (CD_3OD) 6
Figure S7. IR spectrum of **1** 7
Figure S8. HRESIMS spectrum of **1** 7
Figure S9. UV spectrum of **1** 7
Figure S10. CD spectrum of **1** 8
Figure S11. ^{1}H NMR spectrum of **2** (CD_3OD, 500 MHz) 9
Figure S12. ^{13}C NMR spectrum of **2** (CD_3OD, 125 MHz) 9
Figure S13. ^{1}H-^{1}H COSY spectrum of **2** (CD_3OD) 10
Figure S14. HSQC spectrum of **2** (CD_3OD) 10
Figure S15. HMBC spectrum of **2** (CD_3OD) 11
Figure S16. NOESY spectrum of **2** (CD_3OD) 11
Figure S17. IR spectrum of **2** 12
Figure S18. HRESIMS spectrum of **2** 12
Figure S19. UV spectrum of **2** 13
Figure S20. CD spectrum of **2** 13
Figure S21. ^{1}H NMR spectrum of **3** ($CDCl_3$, 500 MHz) 14
Figure S22. ^{13}C NMR spectrum of **3** ($CDCl_3$, 125 MHz) 14
Figure S23. ^{1}H-^{1}H COSY spectrum of **3** ($CDCl_3$) 15
Figure S24. HSQC spectrum of **3** ($CDCl_3$) 15
Figure S25. HMBC spectrum of **3** ($CDCl_3$) 16
Figure S26. NOESY spectrum of **3** ($CDCl_3$) 16
Figure S27. IR spectrum of **3** 17
Figure S28. HRESIMS spectrum of **3** 17
Figure S29. UV spectrum of **3** 17
Figure S30. CD spectrum of **3** 18

Figure S31. ^{1}H NMR spectrum of **4** ($CDCl_3$, 500 MHz) 19
Figure S32. ^{13}C NMR spectrum of **4** ($CDCl_3$, 125 MHz) 19
Figure S33. ^{1}H-^{1}H COSY spectrum of **4** ($CDCl_3$) 20
Figure S34. HSQC spectrum of **4** ($CDCl_3$) 20
Figure S35. HMBC spectrum of **4** ($CDCl_3$) 21
Figure S36. NOESY spectrum of **4** ($CDCl_3$) 21
Figure S37. IR spectrum of **4** 22
Figure S38. HRESIMS spectrum of **4** 22
Figure S39. UV spectrum of **4** 23
Figure S40. CD spectrum of **4** 23
Figure S41. ^{1}H NMR spectrum of **5** ($CDCl_3$, 500 MHz) 24
Figure S42. ^{13}C NMR spectrum of **5** ($CDCl_3$, 125 MHz) 24
Figure S43. ^{1}H-^{1}H COSY spectrum of **5** ($CDCl_3$) 25
Figure S44. HSQC spectrum of **5** ($CDCl_3$) 25
Figure S45. HMBC spectrum of **5** ($CDCl_3$) 26
Figure S46. NOESY spectrum of **5** ($CDCl_3$) 26
Figure S47. IR spectrum of **5** 27
Figure S48. HRESIMS spectrum of **5** 27
Figure S49. UV spectrum of **5** 27
Figure S50. CD spectrum of **5** 28
Table S1. X-ray crystallographic data and structure refinement for **5** 29

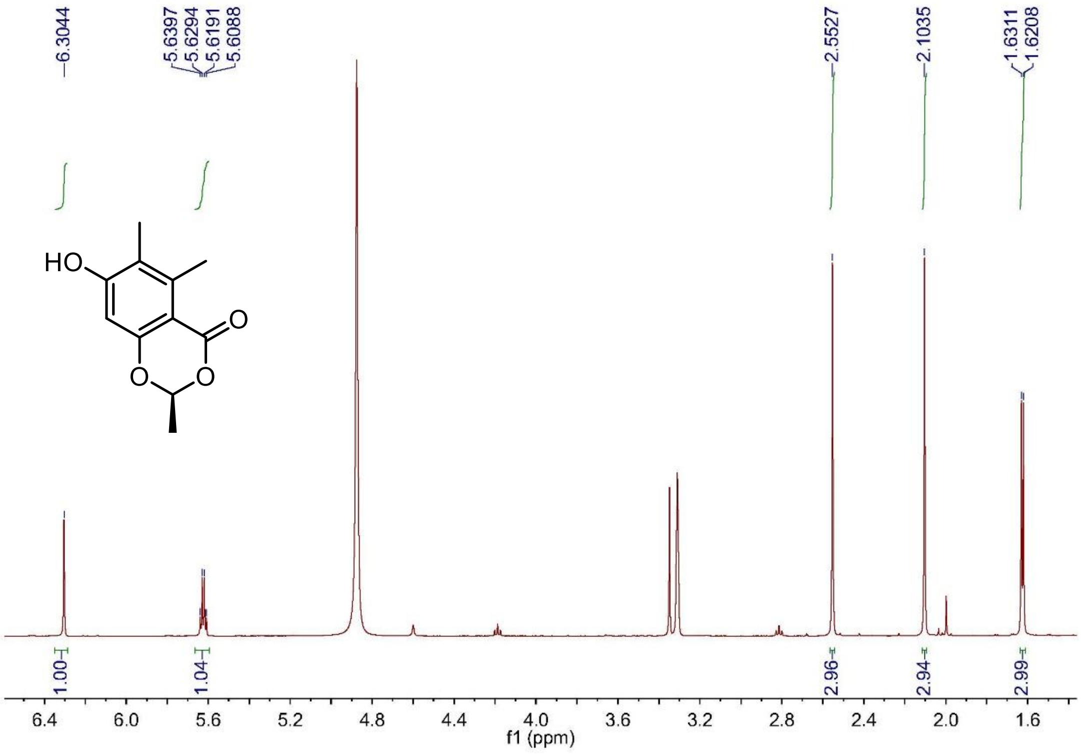

Figure S1. ^{1}H NMR spectrum of **1** (CD_3OD, 500 MHz)

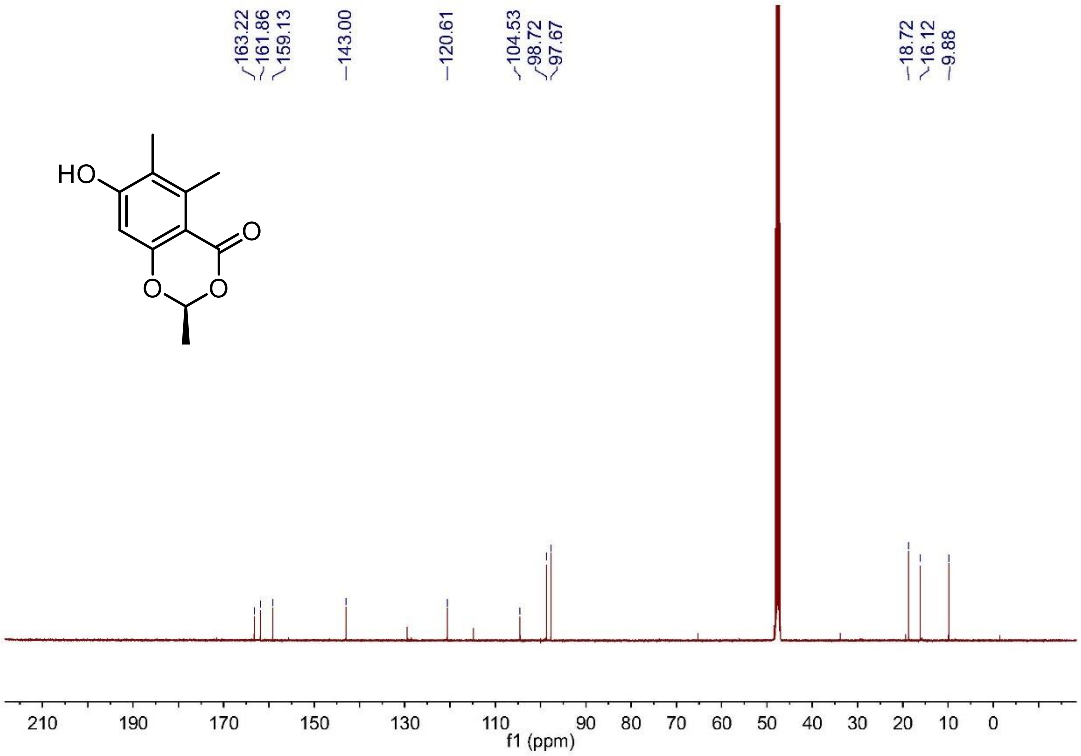

Figure S2. ^{13}C NMR spectrum of 1 (CD_3OD, 125 MHz)

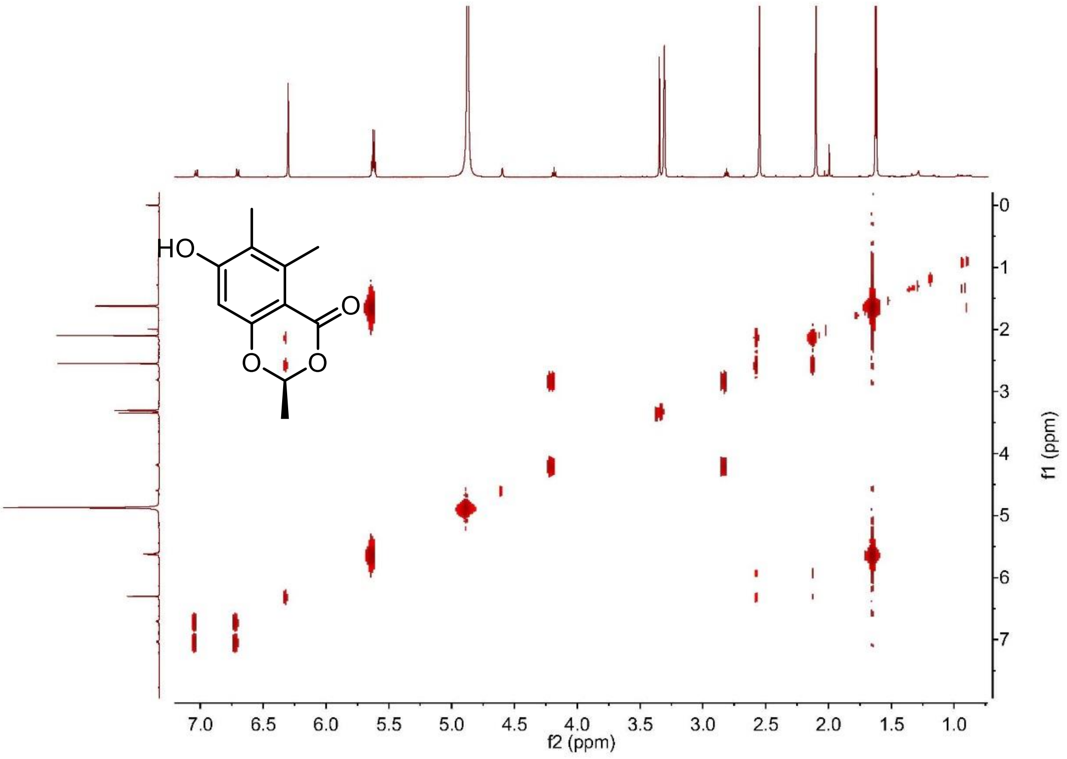

Figure S3. ^{1}H-^{1}H COSY spectrum of **1** (CD_3OD)

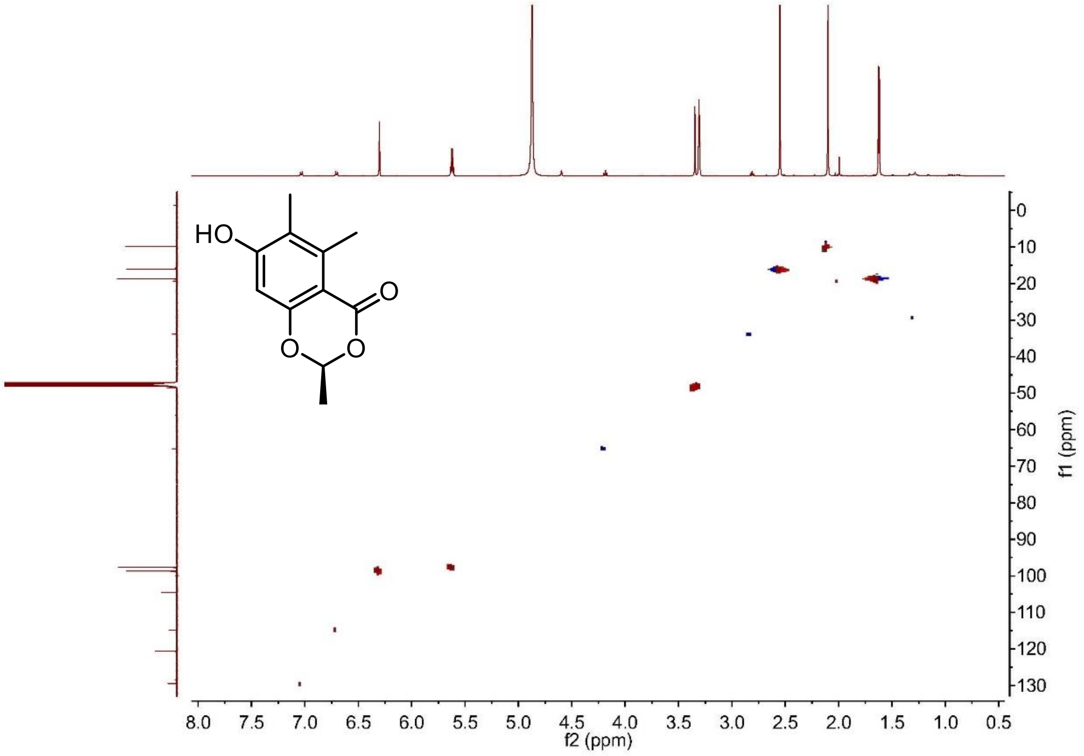

Figure S4. HSQC spectrum of **1** (CD_3OD)

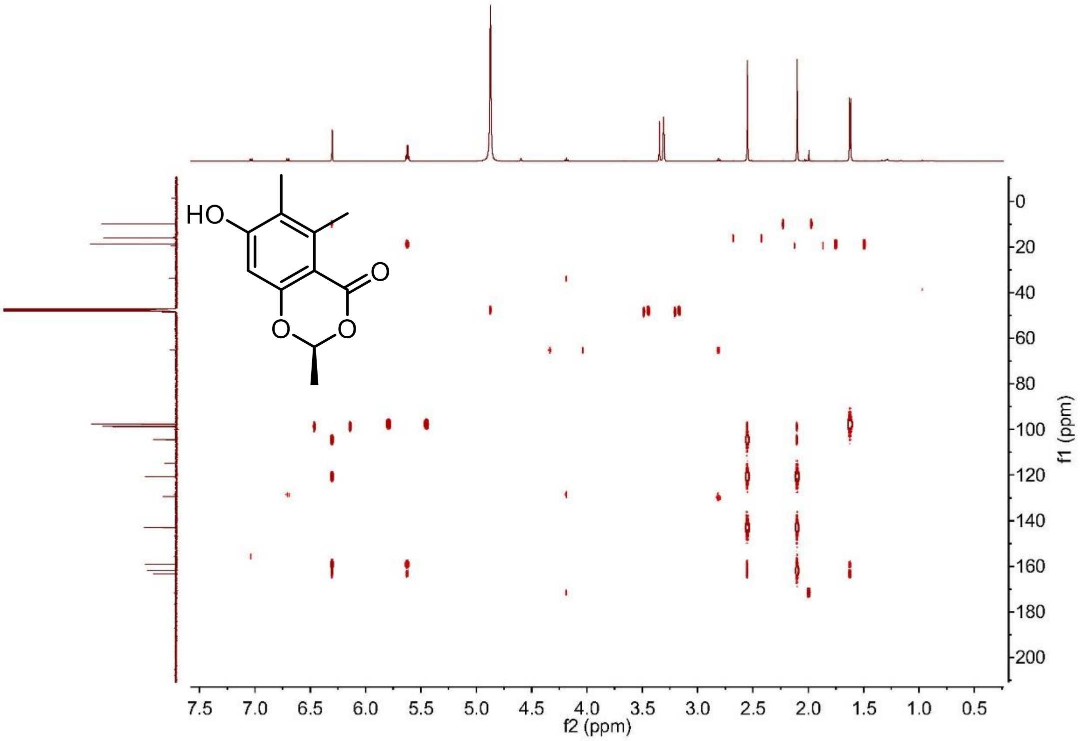

Figure S5. HMBC spectrum of **1** (CD_3OD)

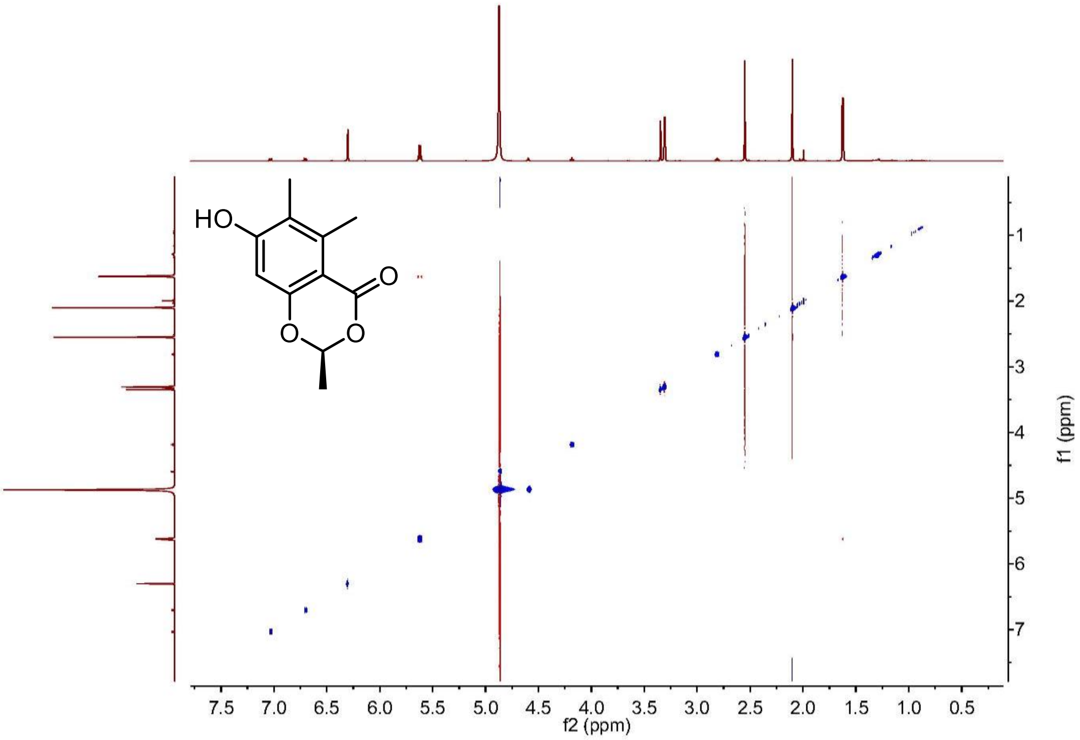

Figure S6. NOESY spectrum of **1** (CD_3OD)

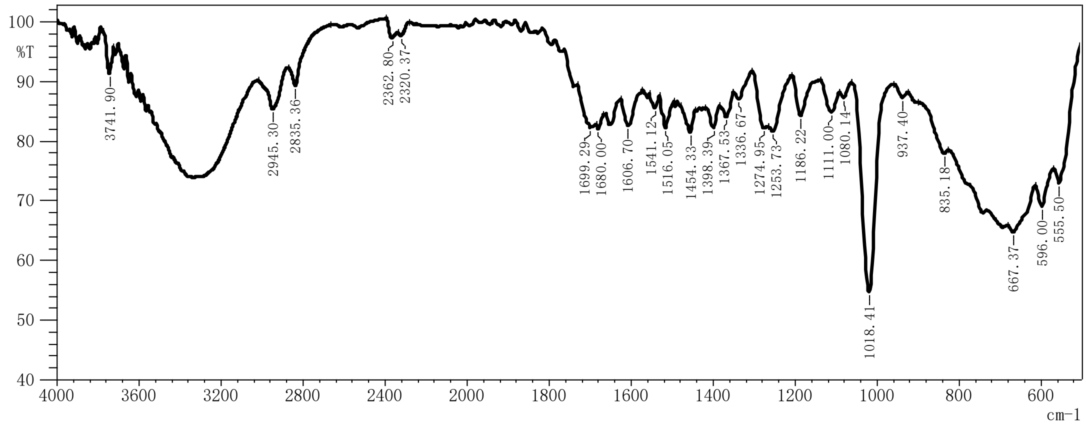

Figure S7. IR spectrum of **1**

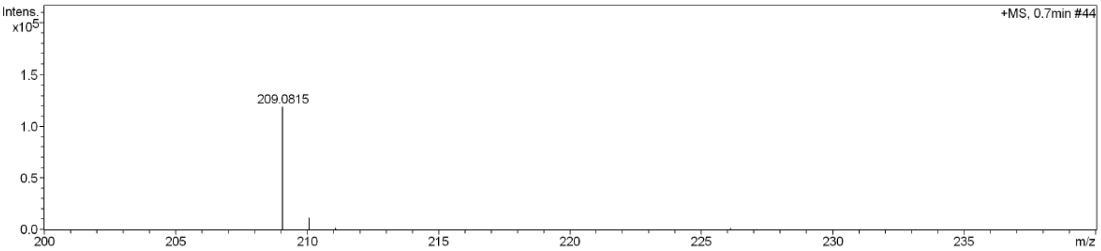

Meas. m/z	#	Ion Formula	Score	m/z	err [ppm]	err [mDa]	mSigma	rdb	e⁻ Conf	N-Rule
209.0815	1	C11H13O4	100.00	209.0808	-3.0	-0.6	15.3	5.5	even	ok
231.0625	1	C11H12NaO4	100.00	231.0628	1.2	0.3	9.0	5.5	even	ok

Figure S8. HRESIMS spectrum of **1**

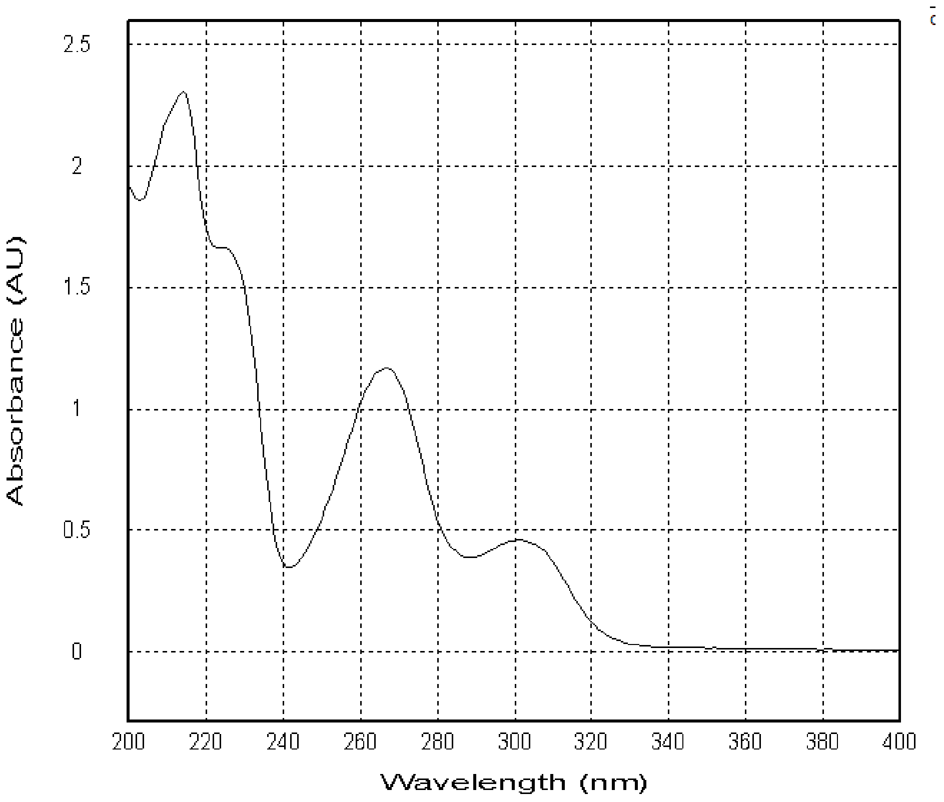

Figure S9. UV spectrum of **1**

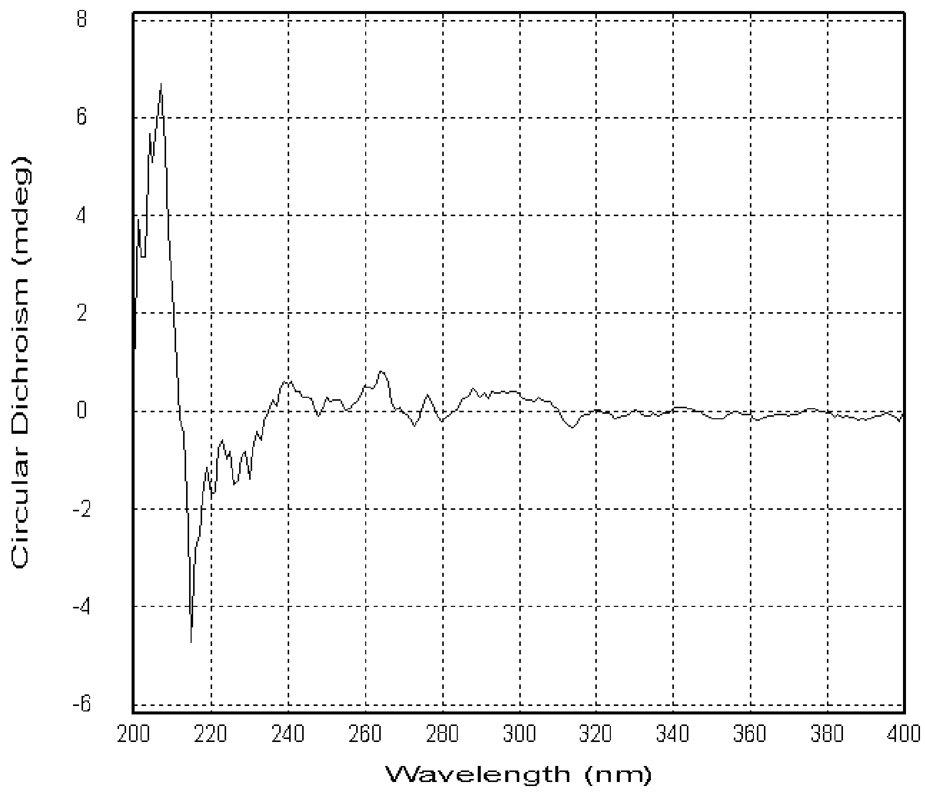

Figure S10. CD spectrum of **1**

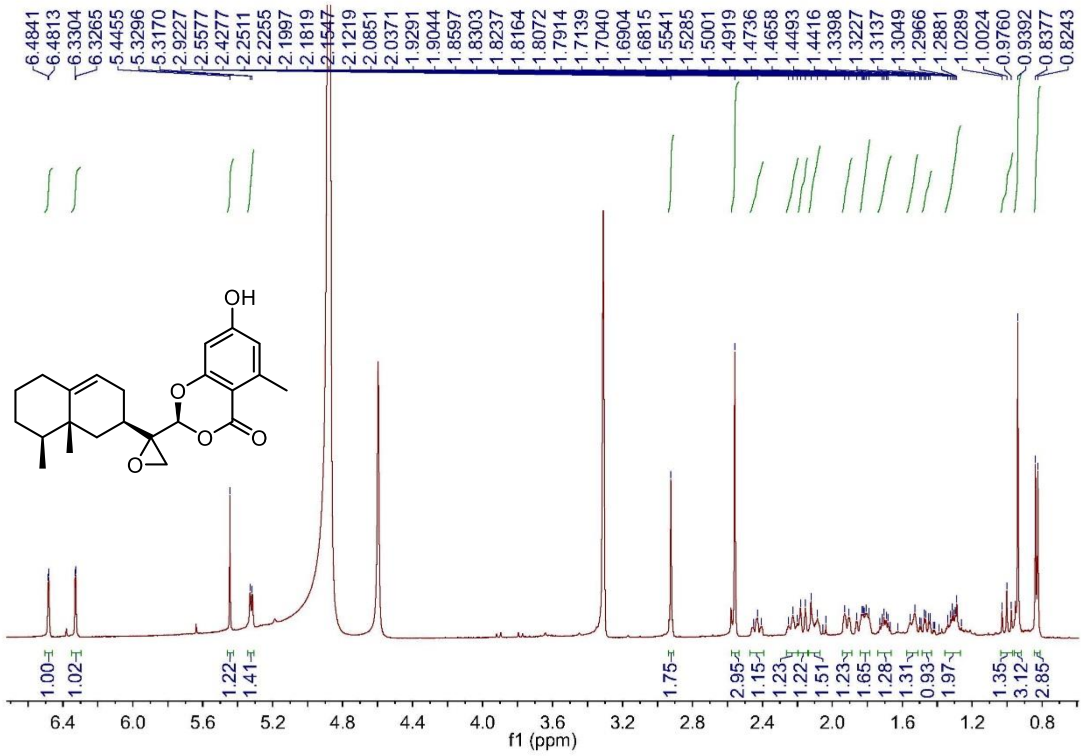

Figure S11. ^{1}H NMR spectrum of **2** (CD_3OD, 500 MHz)

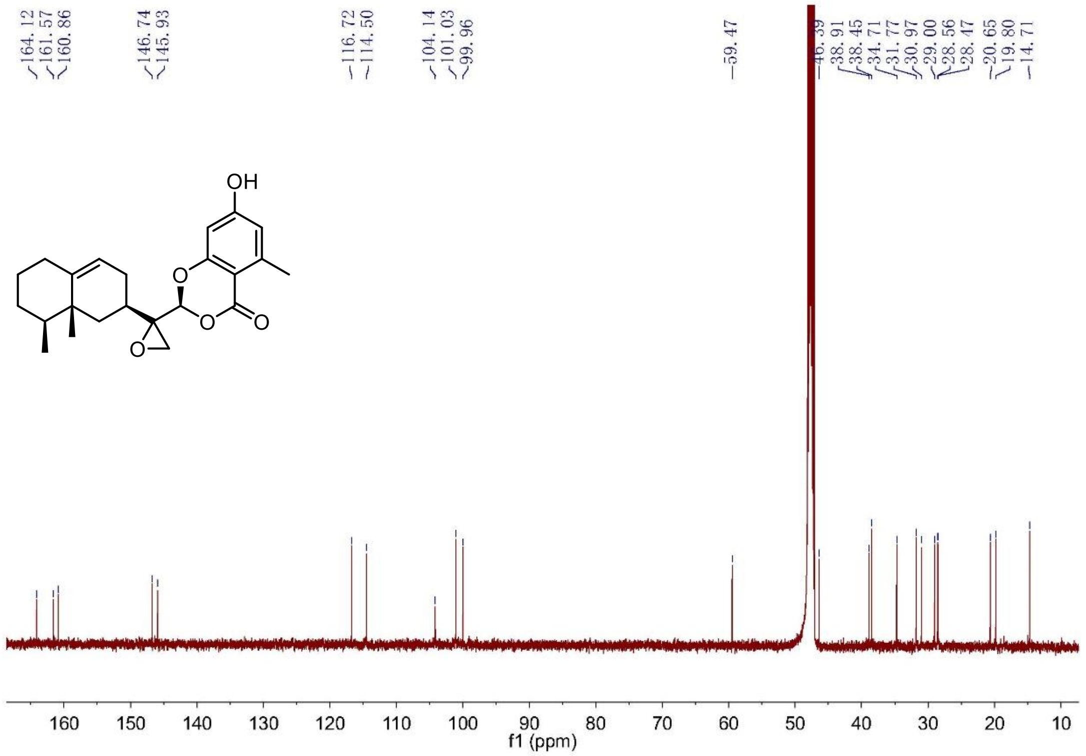

Figure S12. ^{13}C NMR spectrum of **2** (CD_3OD, 125 MHz)

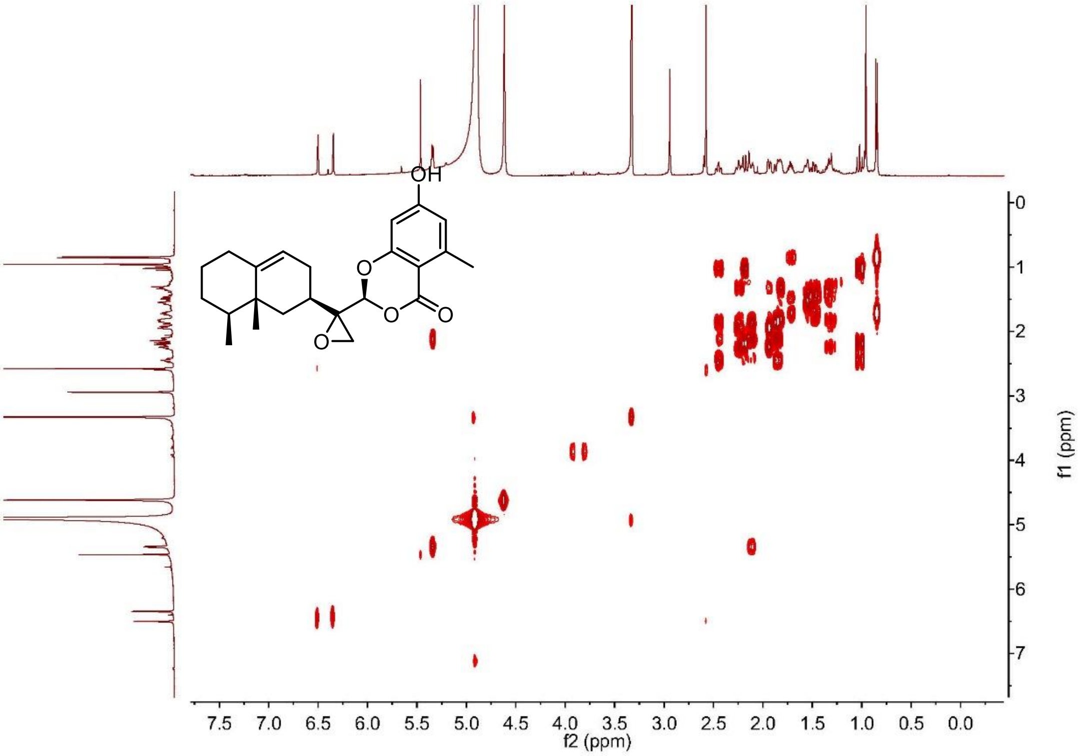

Figure S13. ^{1}H-^{1}H COSY spectrum of **2** (CD_3OD)

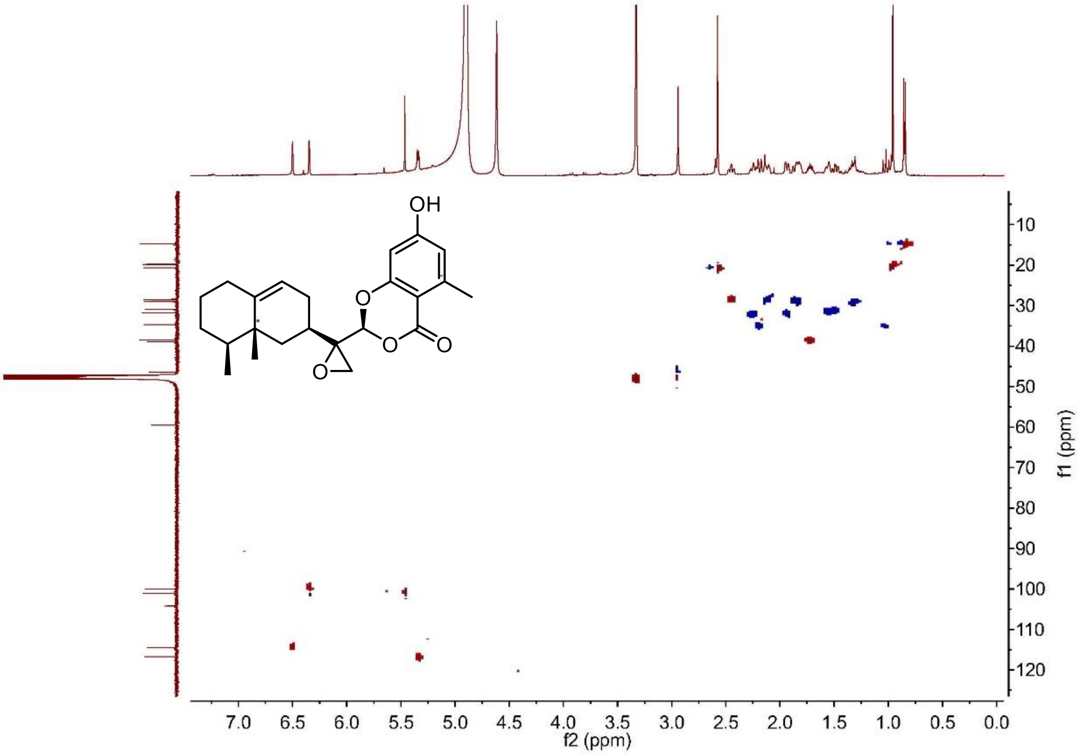

Figure S14. HSQC spectrum of **2** (CD_3OD)

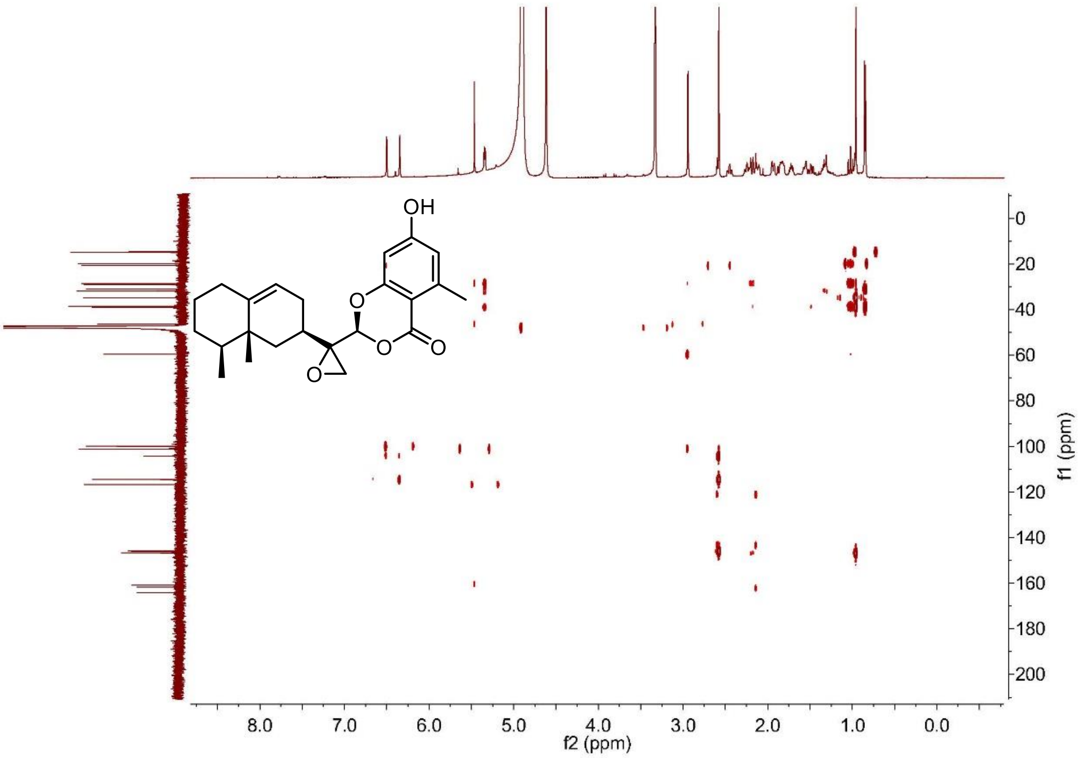

Figure S15. HMBC spectrum of **2** (CD_3OD)

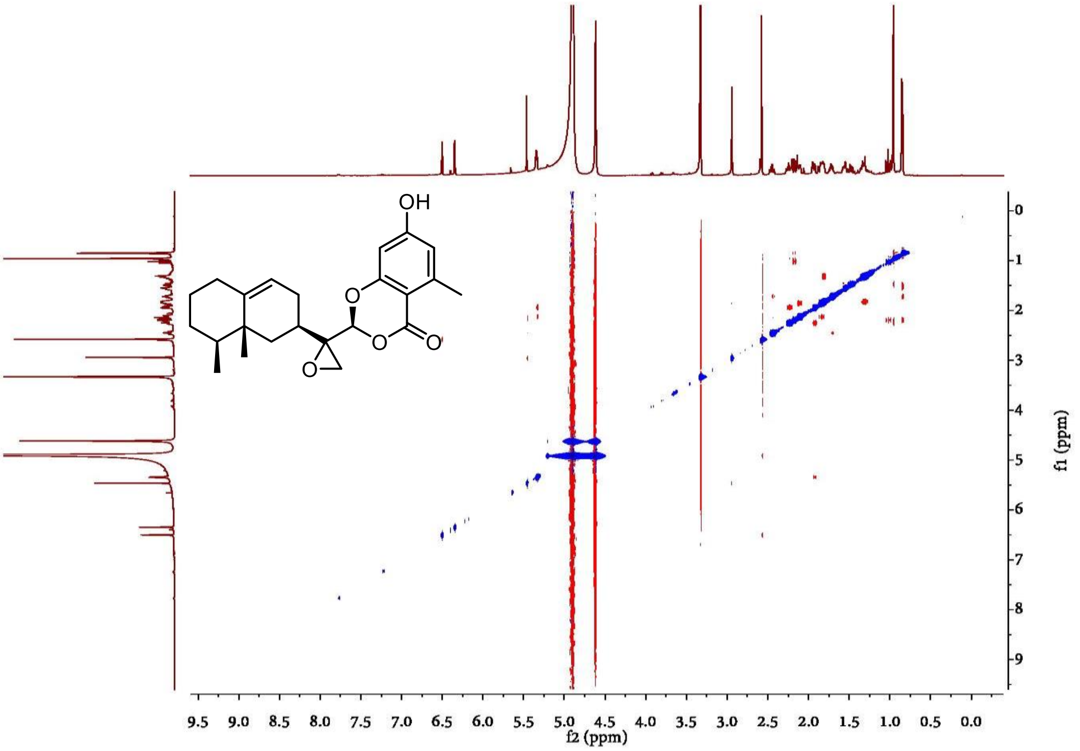

Figure S16. NOESY spectrum of **2** (CD_3OD)

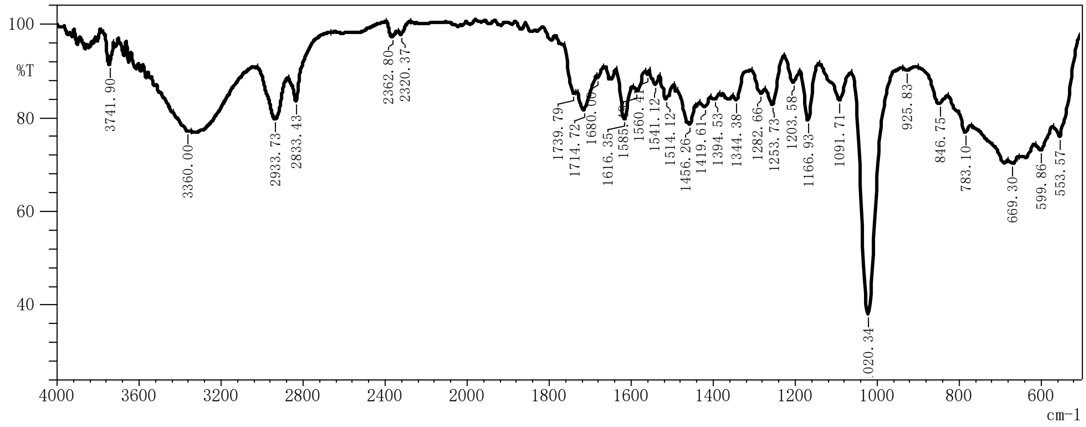

Figure S17. IR spectrum of **2**

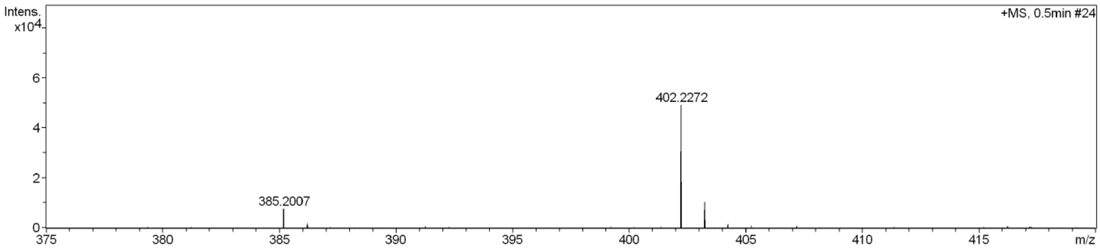

Meas. m/z	#	Ion Formula	Score	m/z	err [ppm]	err [mDa]	mSigma	rdb	e⁻ Conf	N-Rule
385.2007	1	C23H29O5	100.00	385.2010	-0.6	-0.2	32.0	9.5	even	ok
402.2272	1	C23H32NO5	100.00	402.2275	0.7	0.3	24.5	8.5	even	ok
407.1837	1	C23H28NaO5	100.00	407.1829	-1.9	-0.8	65.6	9.5	even	ok

Figure S18. HRESIMS spectrum of **2**

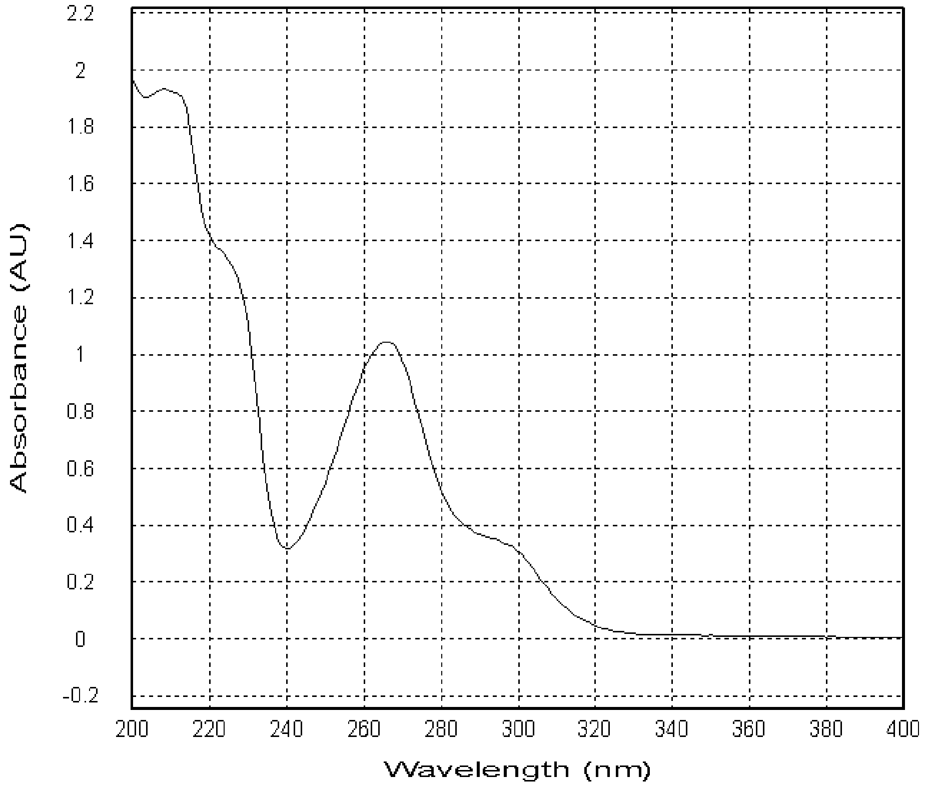

Figure S19. UV spectrum of **2**

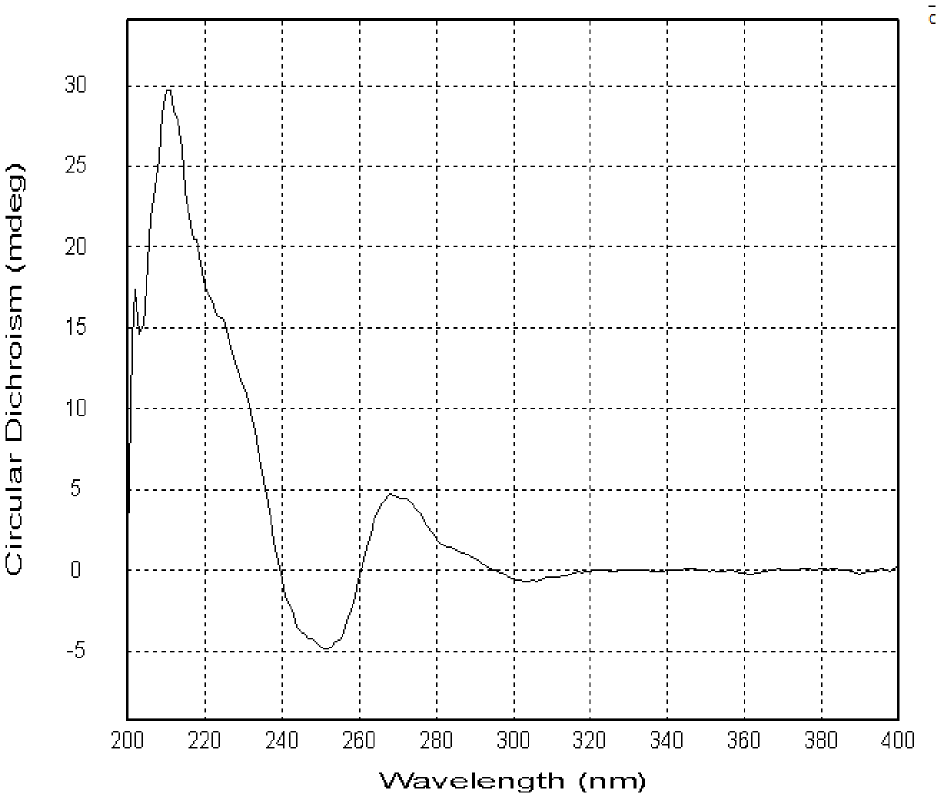

Figure S20. CD spectrum of **2**

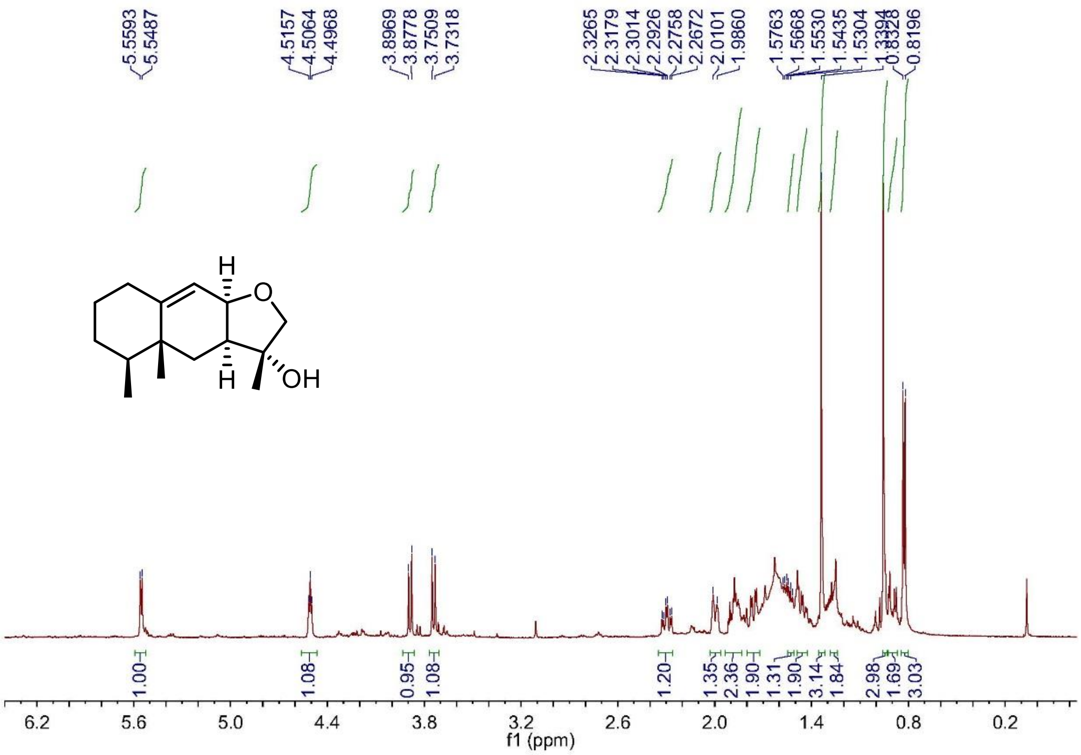

Figure S21. ^{1}H NMR spectrum of **3** ($CDCl_3$, 500 MHz)

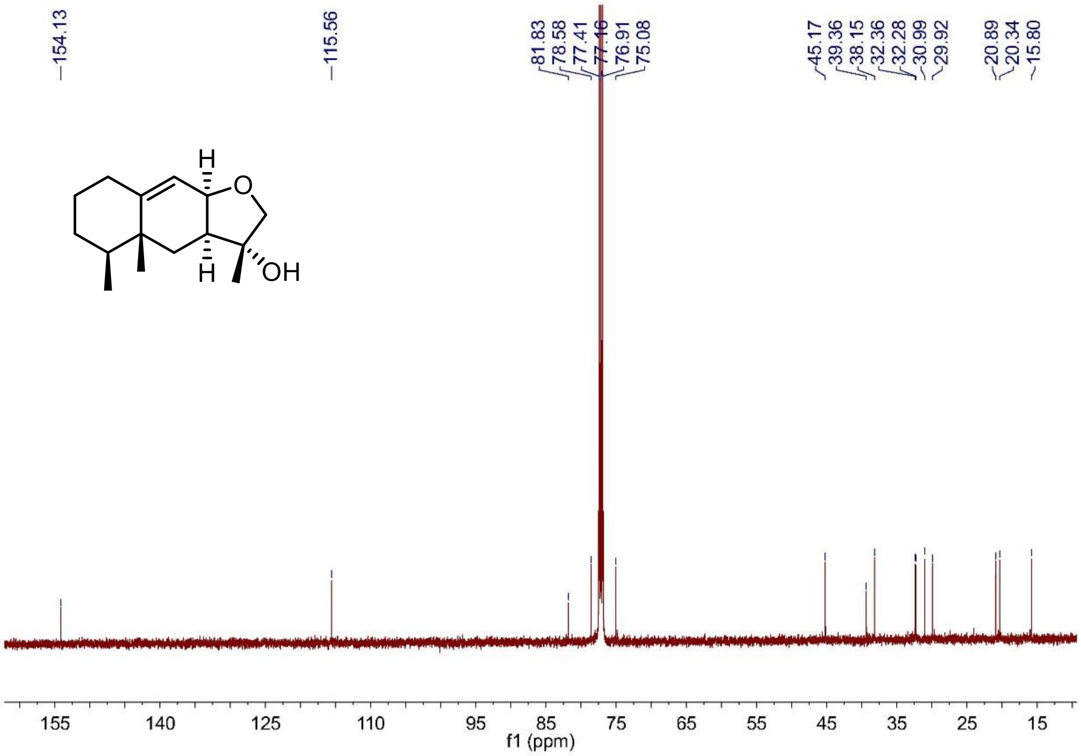

Figure S22. ^{13}C NMR spectrum of **3** ($CDCl_3$, 125 MHz)

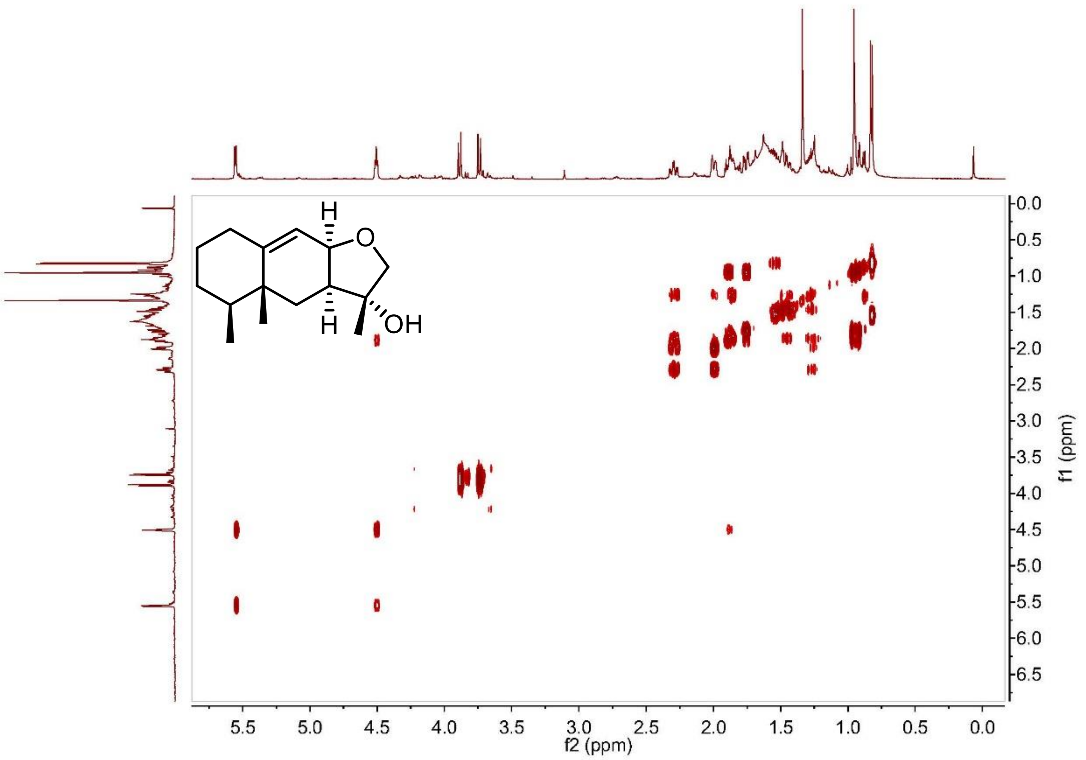

Figure S23. ^{1}H-^{1}H COSY spectrum of **3** ($CDCl_3$)

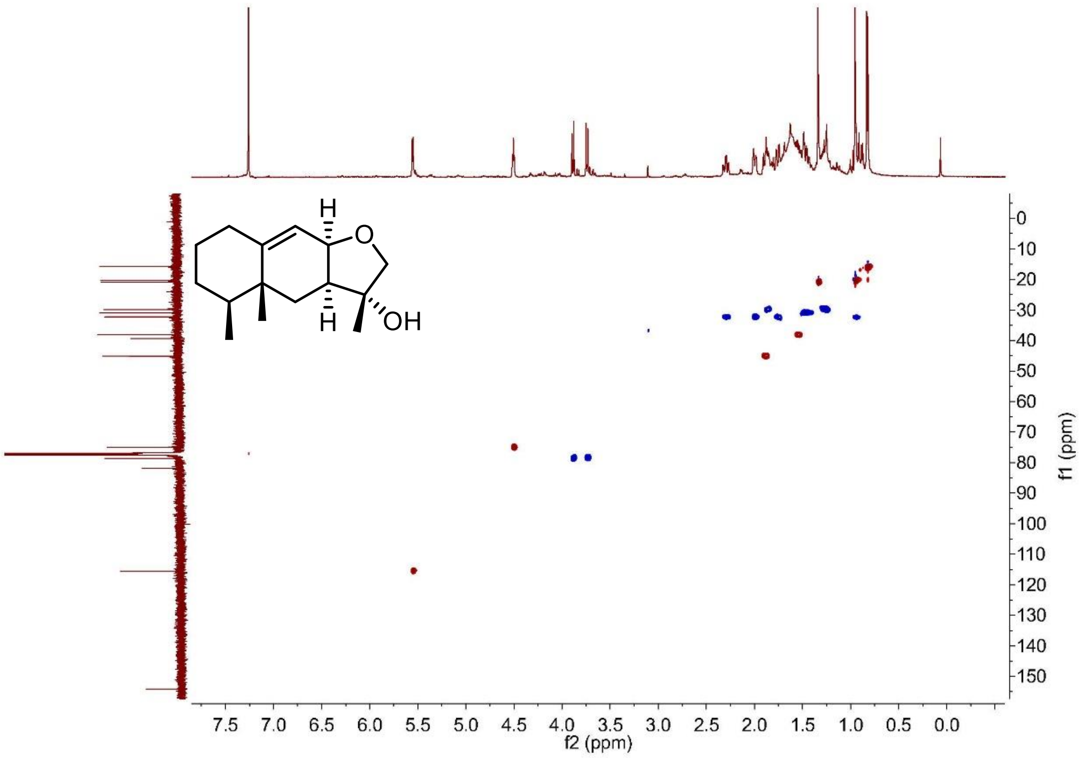

Figure S24. HSQC spectrum of **3** ($CDCl_3$)

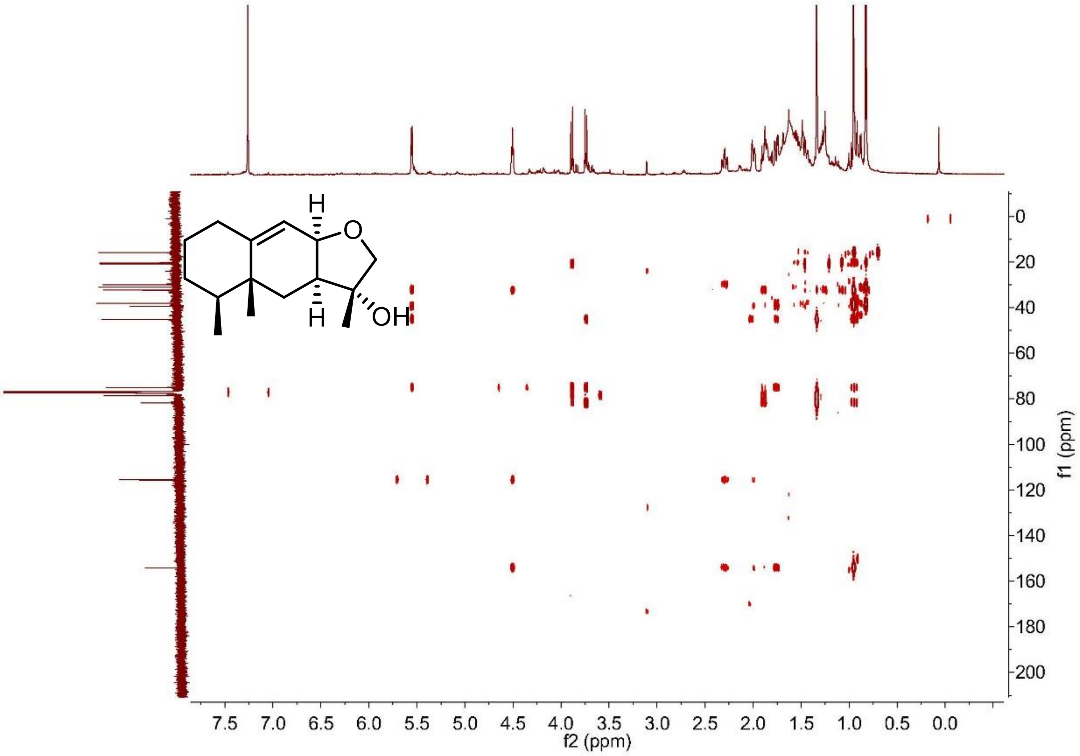

Figure S25. HMBC spectrum of **3** ($CDCl_3$)

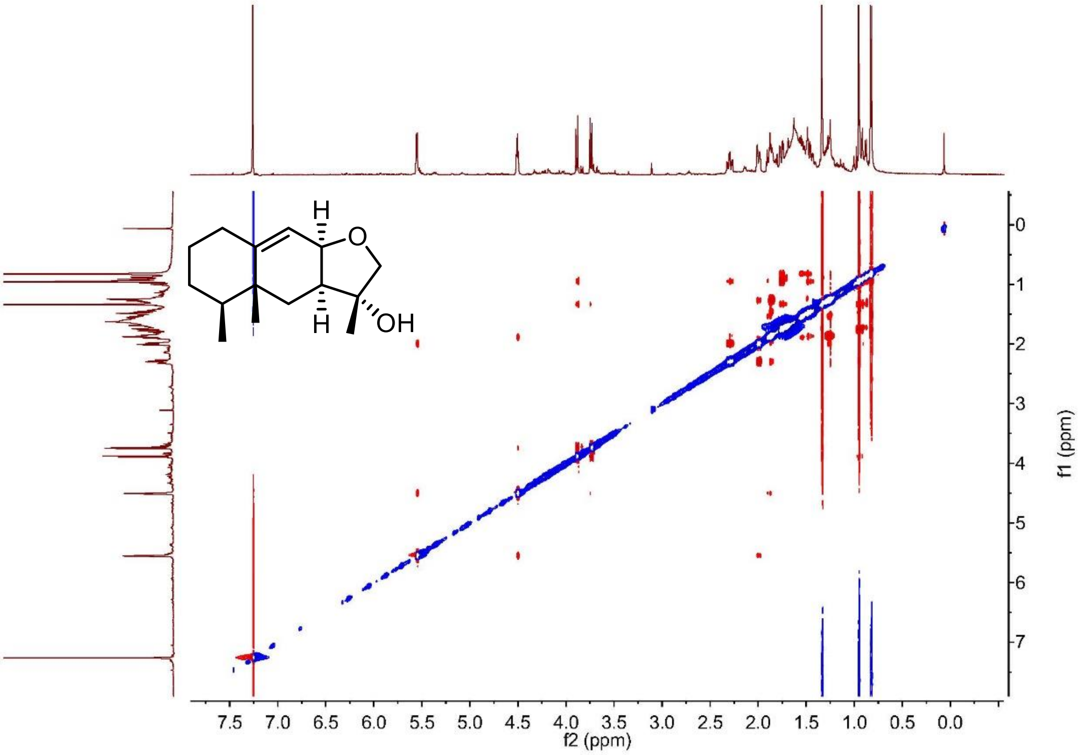

Figure S26. NOESY spectrum of **3** ($CDCl_3$)

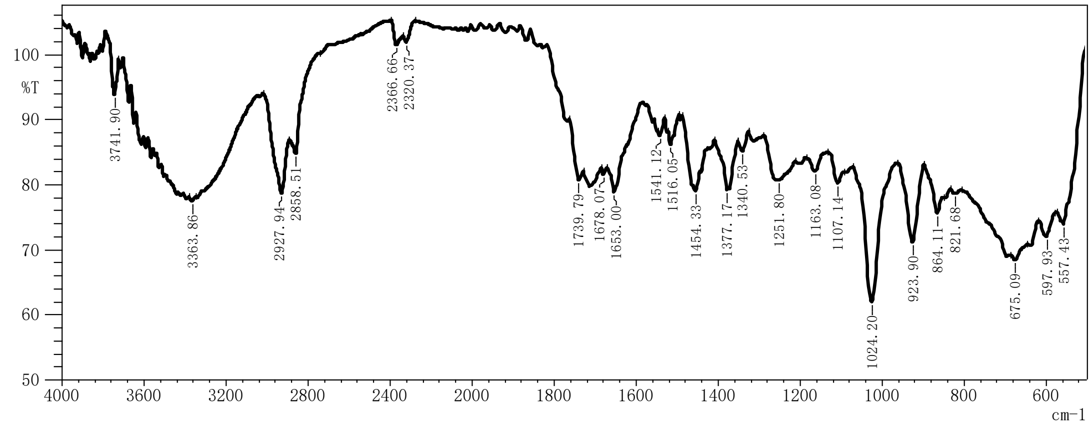

Figure S27. IR spectrum of **3**

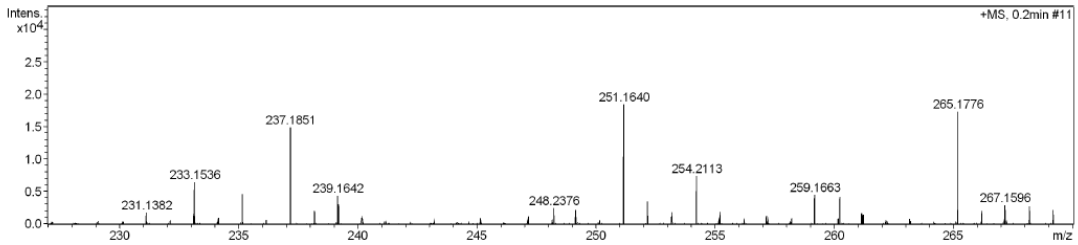

Meas. m/z	#	Ion Formula	Score	m/z	err [ppm]	err [mDa]	mSigma	rdb	e⁻ Conf	N-Rule
237.1851	1	C15H25O2	100.00	237.1849	0.7	0.2	20.7	3.5	even	ok
254.2113	1	C15H28NO2	100.00	254.2115	0.4	0.1	111.4	2.5	even	ok
259.1663	1	C15H24NaO2	100.00	259.1669	-2.0	-0.5	11.5	3.5	even	ok

Figure S28. HRESIMS spectrum of **3**

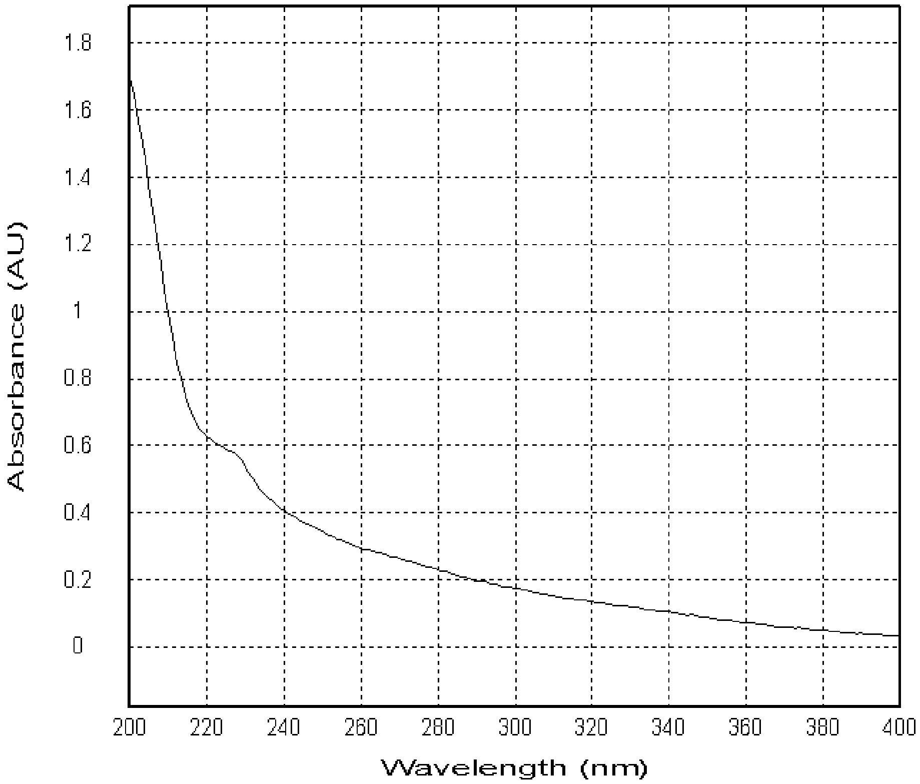

Figure S29. UV spectrum of **3**

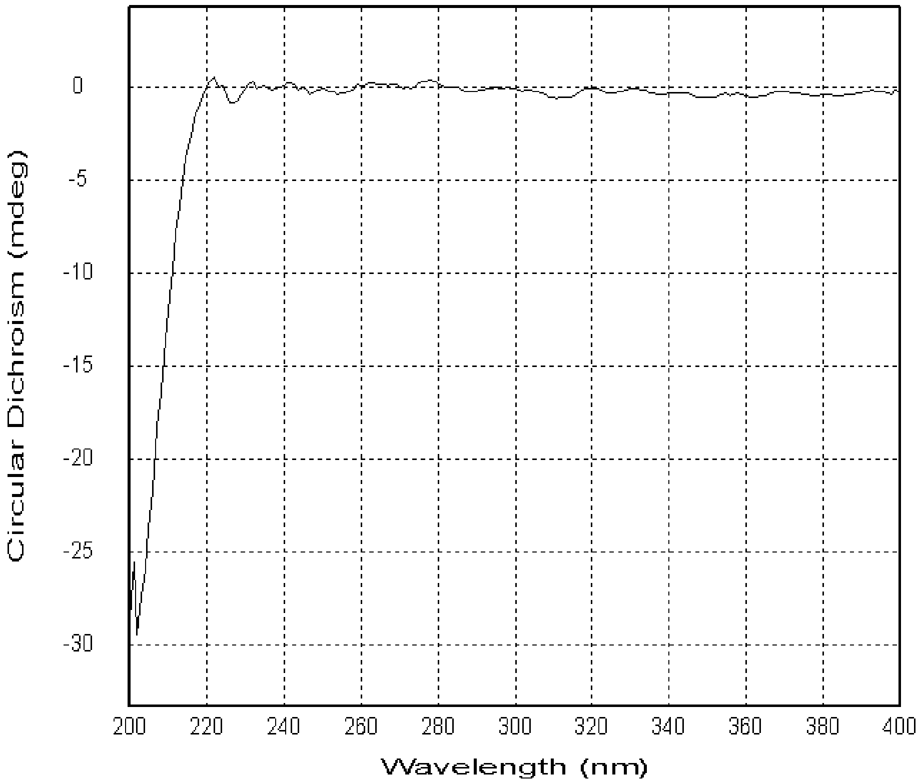

Figure S30. CD spectrum of **3**

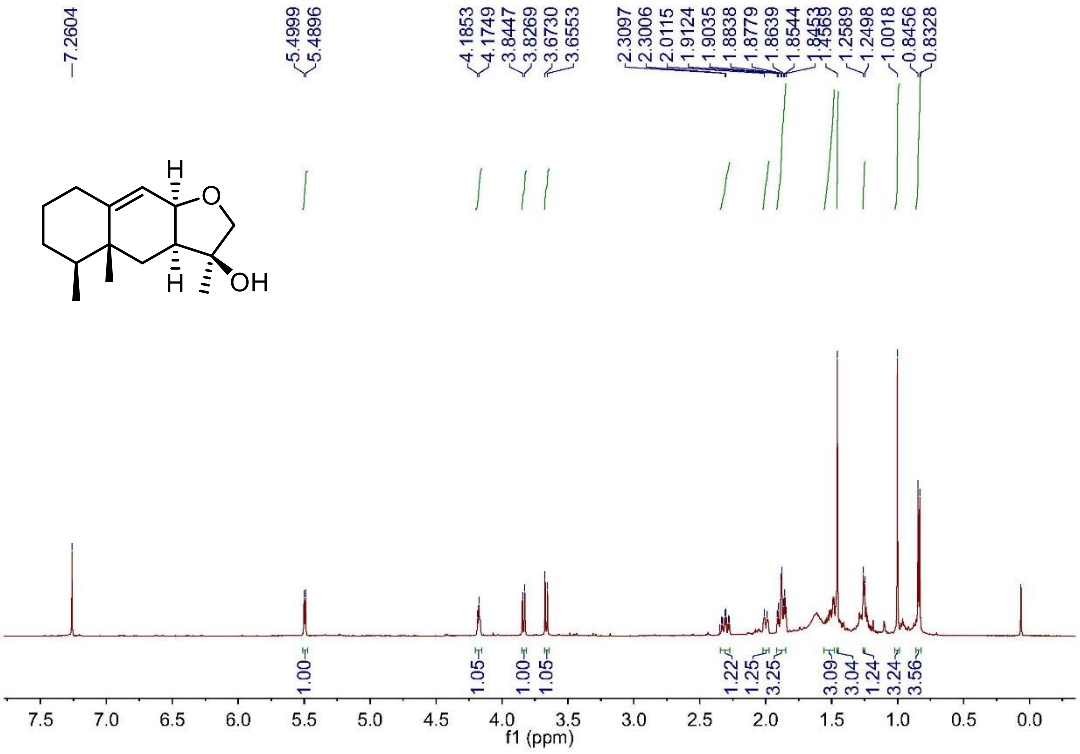

Figure S31. ^{1}H NMR spectrum of **4** ($CDCl_3$, 500 MHz)

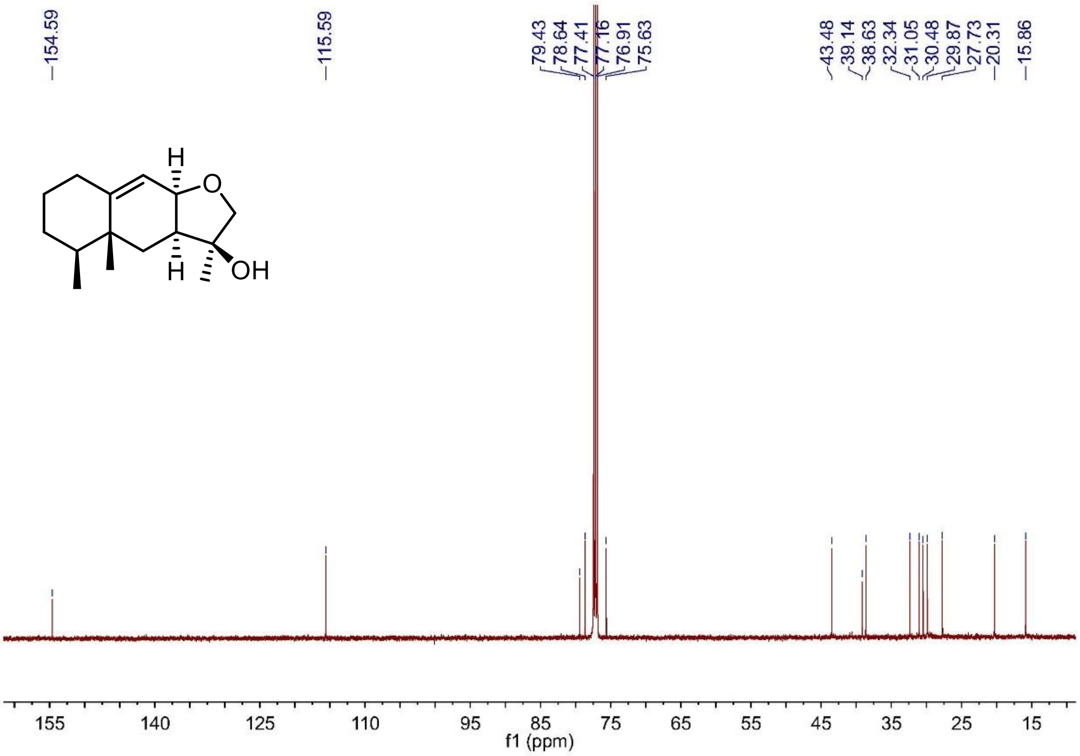

Figure S32. ^{13}C NMR spectrum of **4** ($CDCl_3$, 125 MHz)

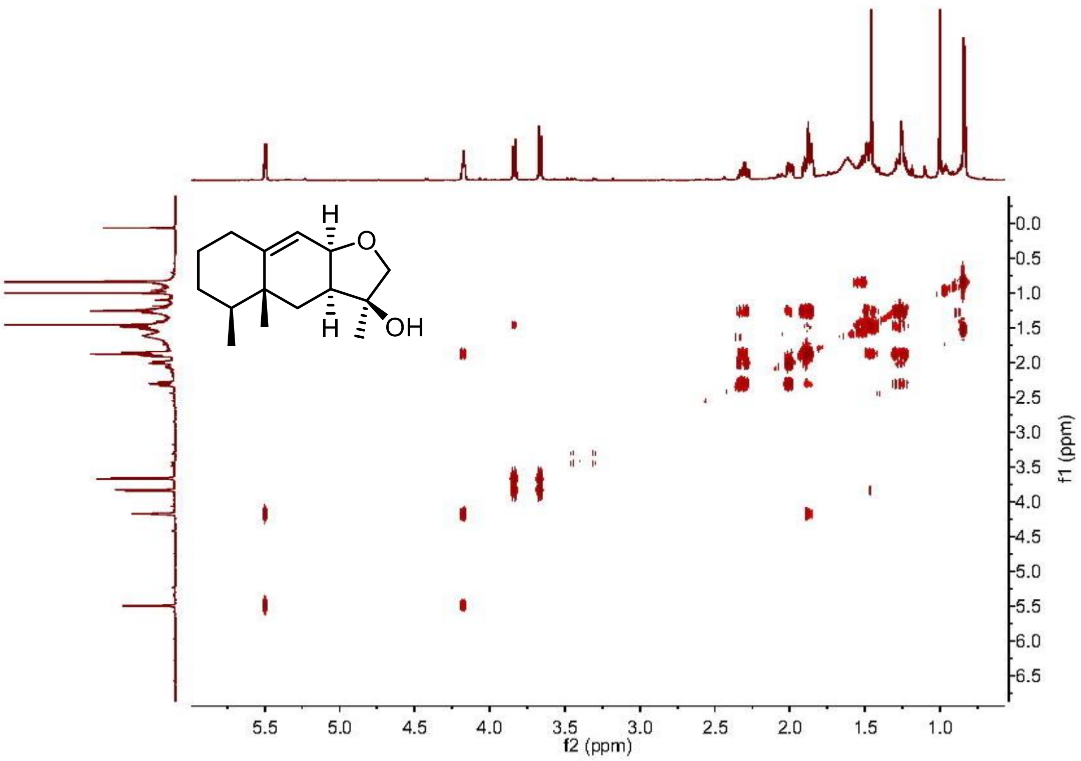

Figure S33. ^{1}H-^{1}H COSY spectrum of **4** ($CDCl_3$)

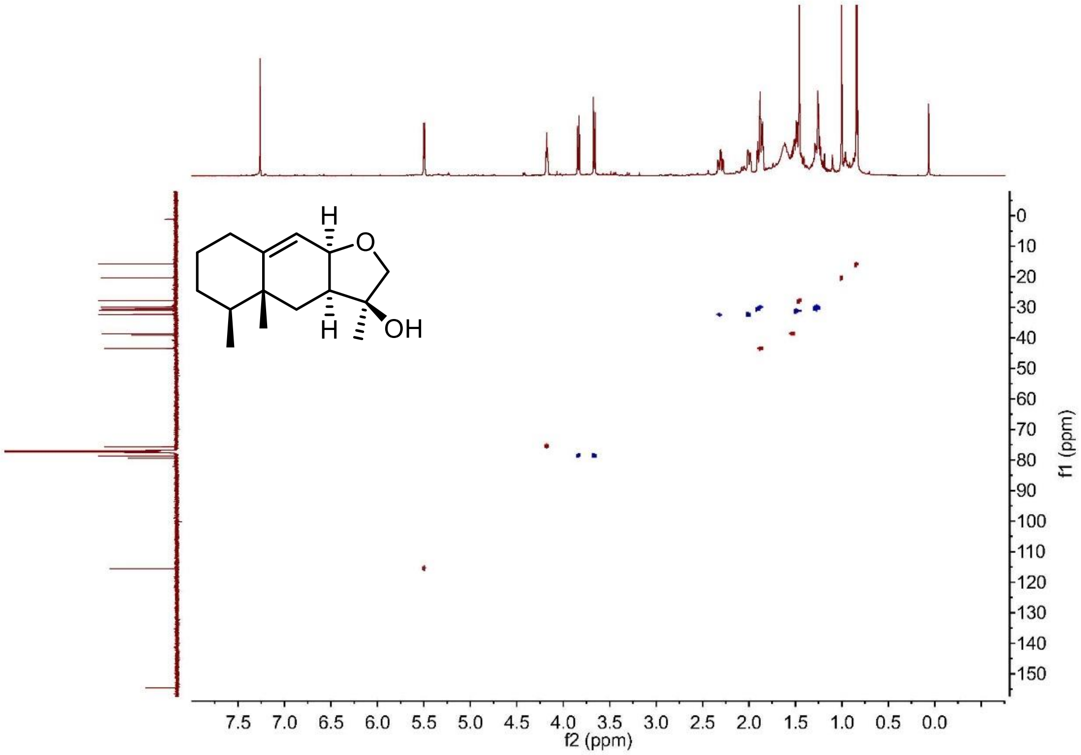

Figure S34. HSQC spectrum of **4** ($CDCl_3$)

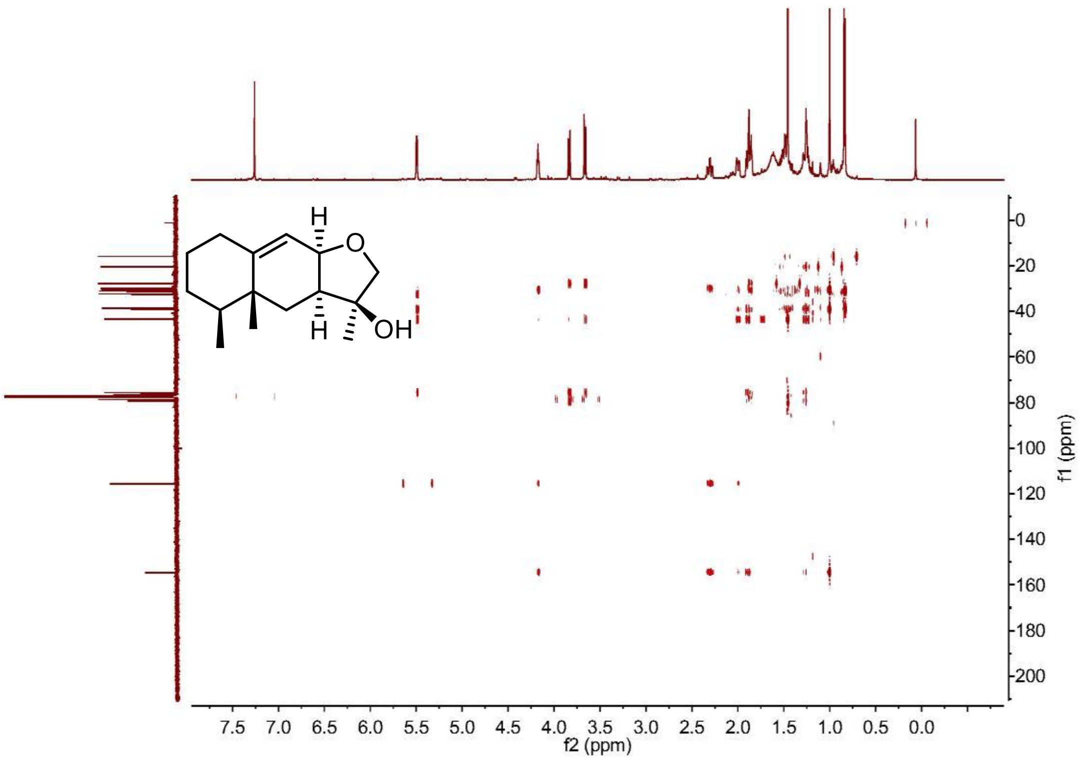

Figure S35. HMBC spectrum of **4** ($CDCl_3$)

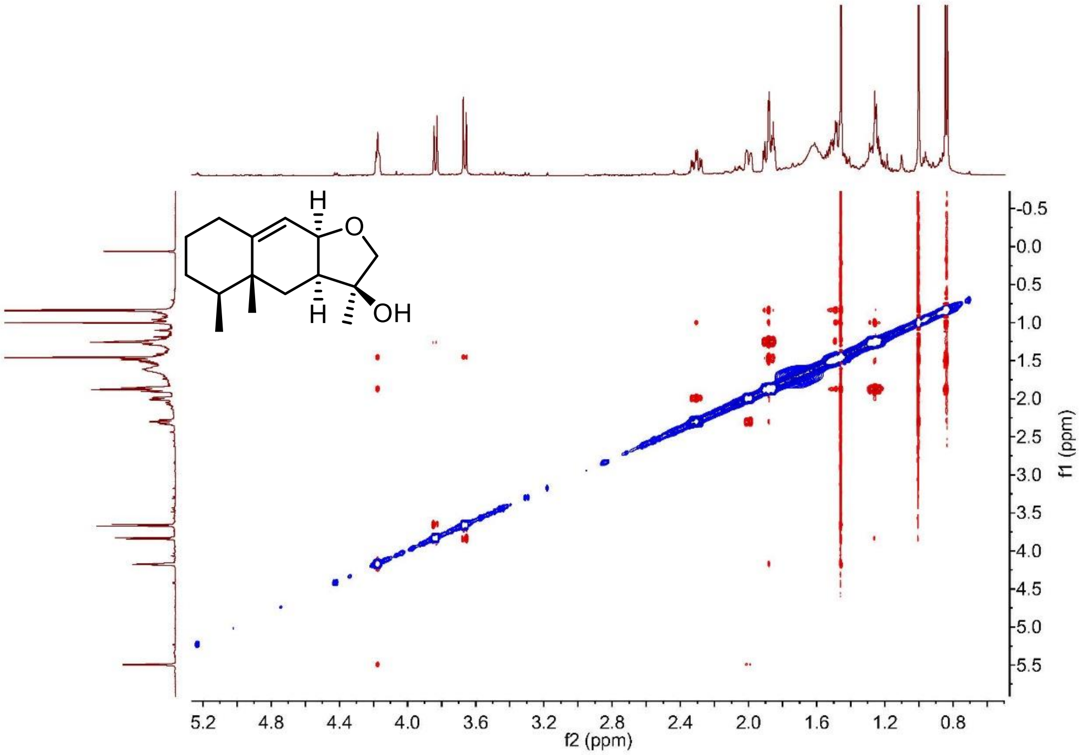

Figure S36. NOESY spectrum of **4** ($CDCl_3$)

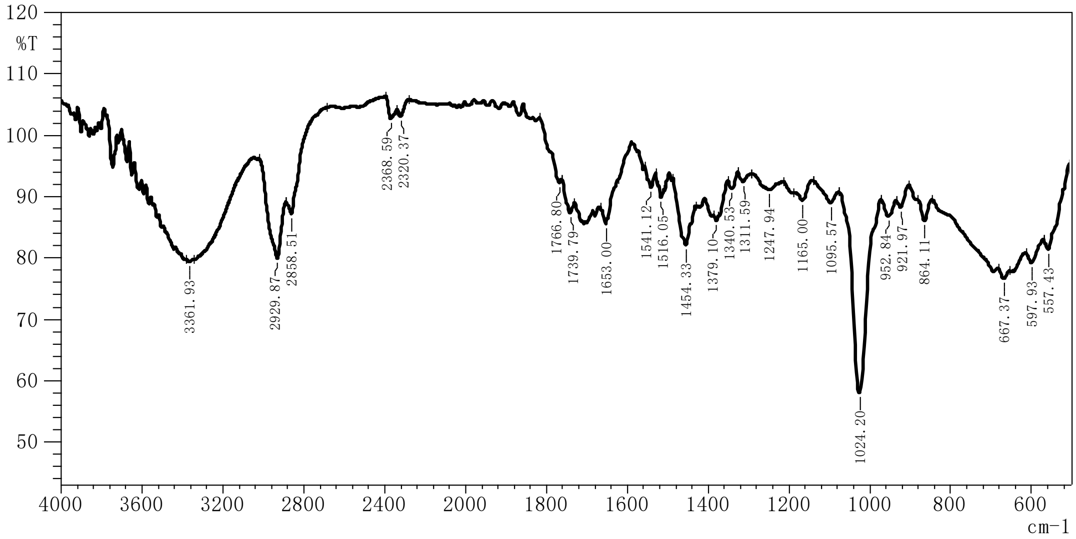

Figure S37. IR spectrum of **4**

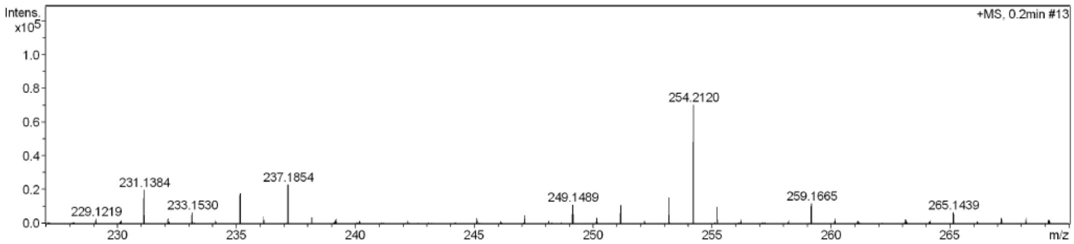

Meas. m/z	#	Ion Formula	Score	m/z	err [ppm]	err [mDa]	mSigma	rdb	e⁻ Conf	N-Rule
237.1854	1	C15H25O2	100.00	237.1849	2.3	0.5	29.2	3.5	even	ok
254.2120	1	C15H28NO2	100.00	254.2115	-2.3	-0.6	17.7	2.5	even	ok
259.1665	1	C15H24NaO2	100.00	259.1669	1.4	0.4	20.7	3.5	even	ok

Figure S38. HRESIMS spectrum of **4**

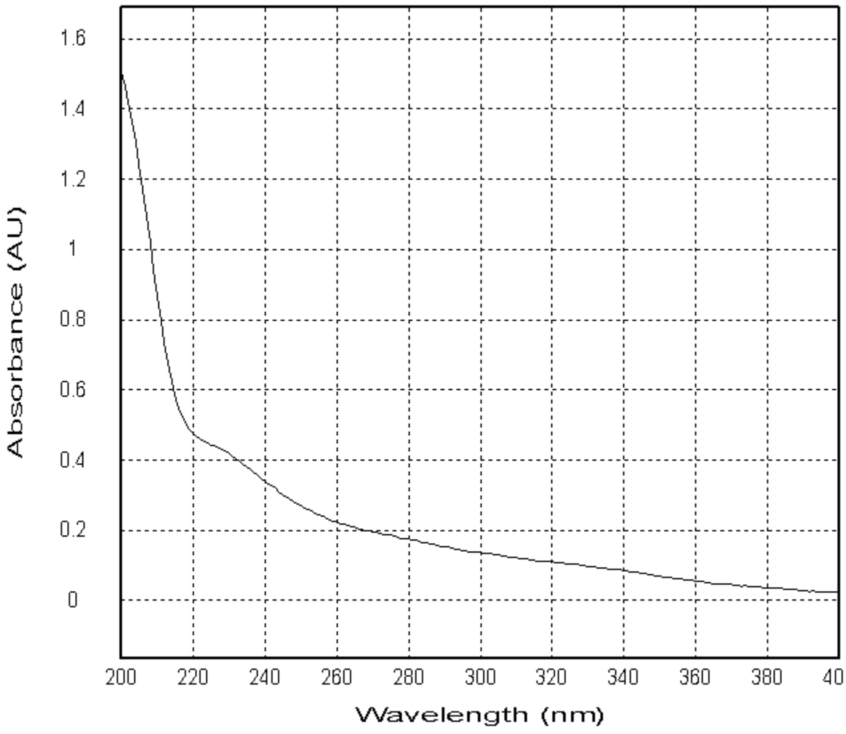

Figure S39. UV spectrum of **4**

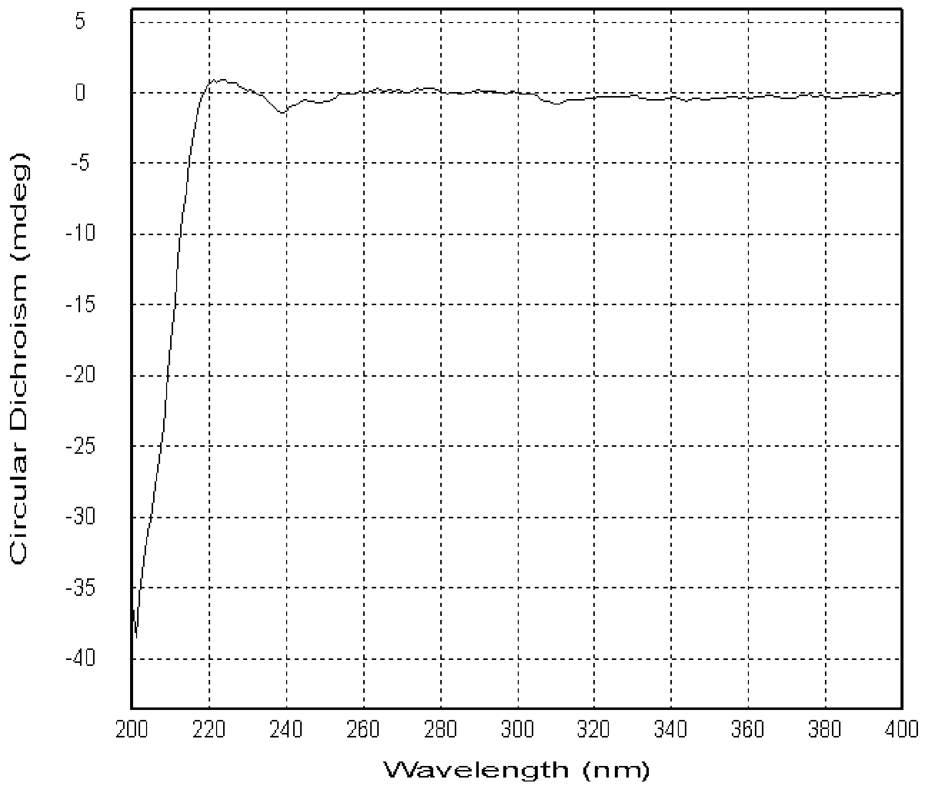

Figure S40. CD spectrum of **4**

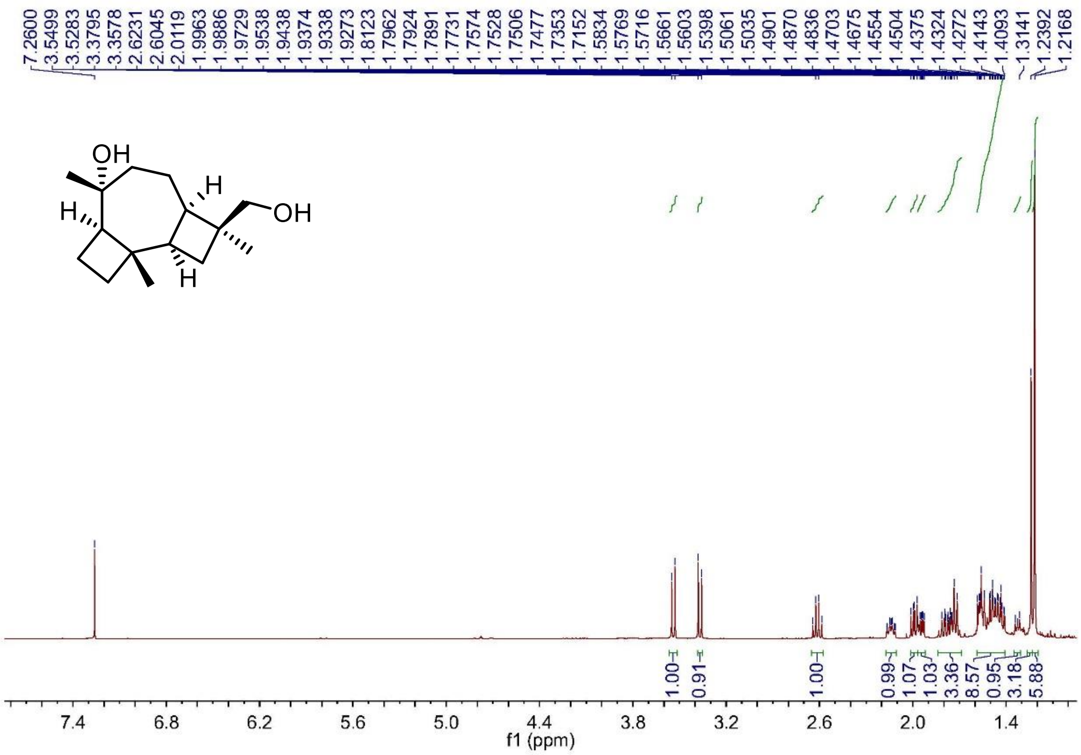

Figure S41. ^{1}H NMR spectrum of **5** ($CDCl_3$, 500 MHz)

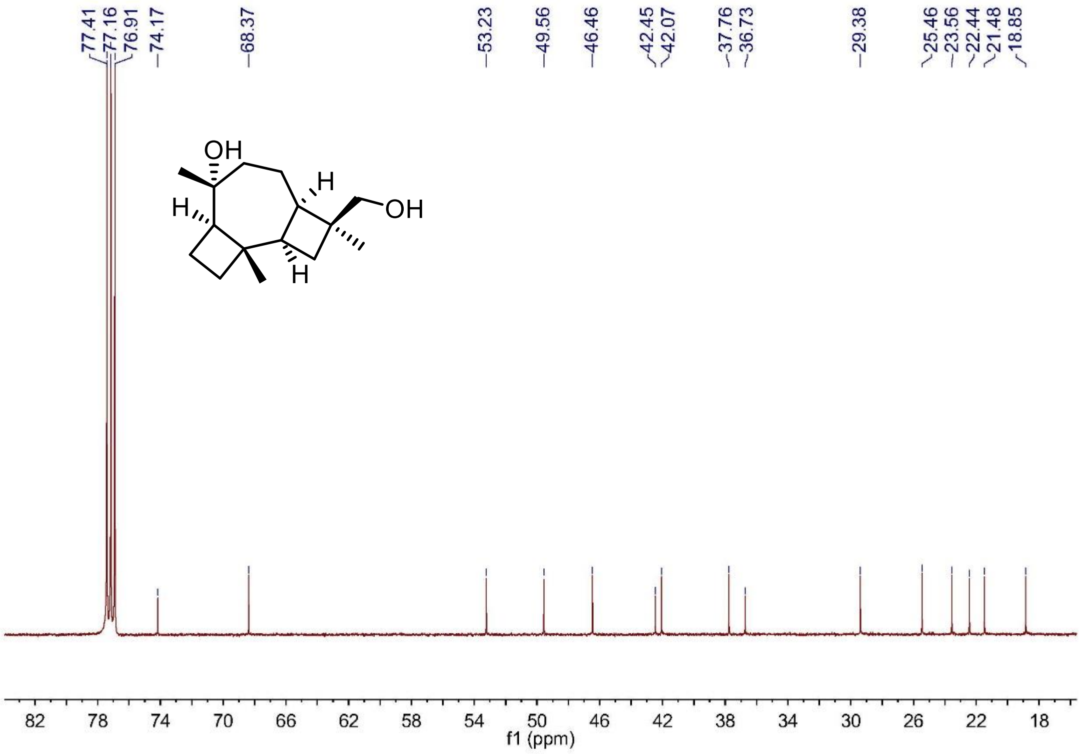

Figure S42. ^{13}C NMR spectrum of **5** ($CDCl_3$, 125 MHz)

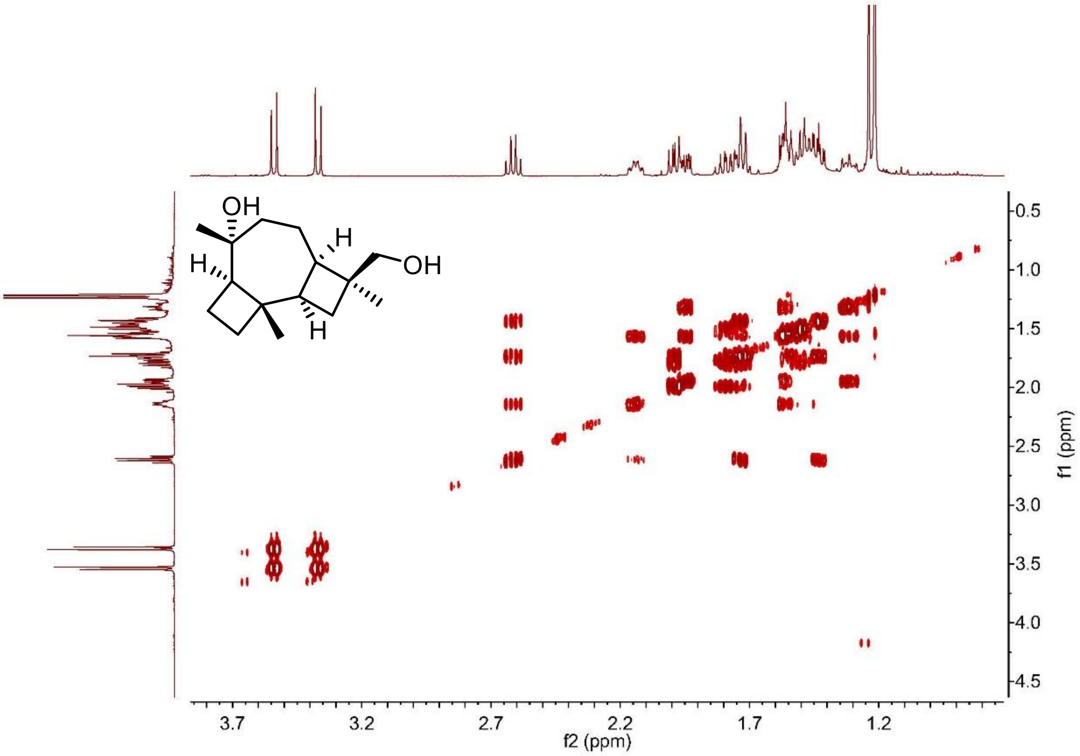

Figure S43. ^{1}H-^{1}H COSY spectrum of **5** ($CDCl_3$)

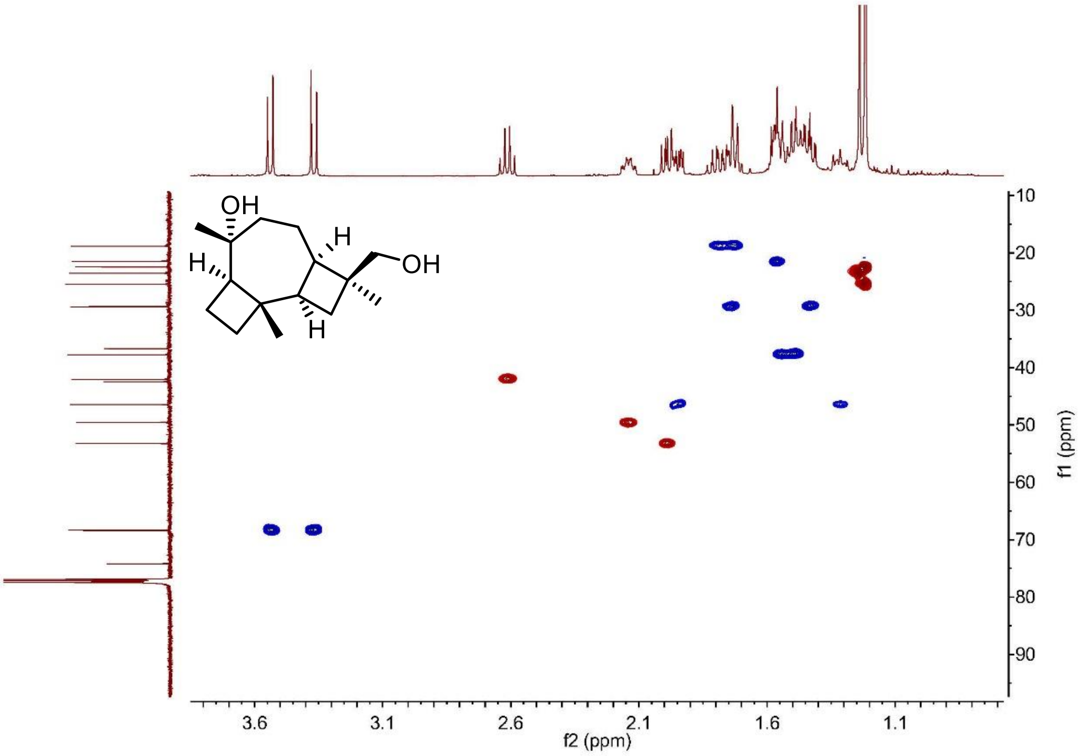

Figure S44. HSQC spectrum of **5** ($CDCl_3$)

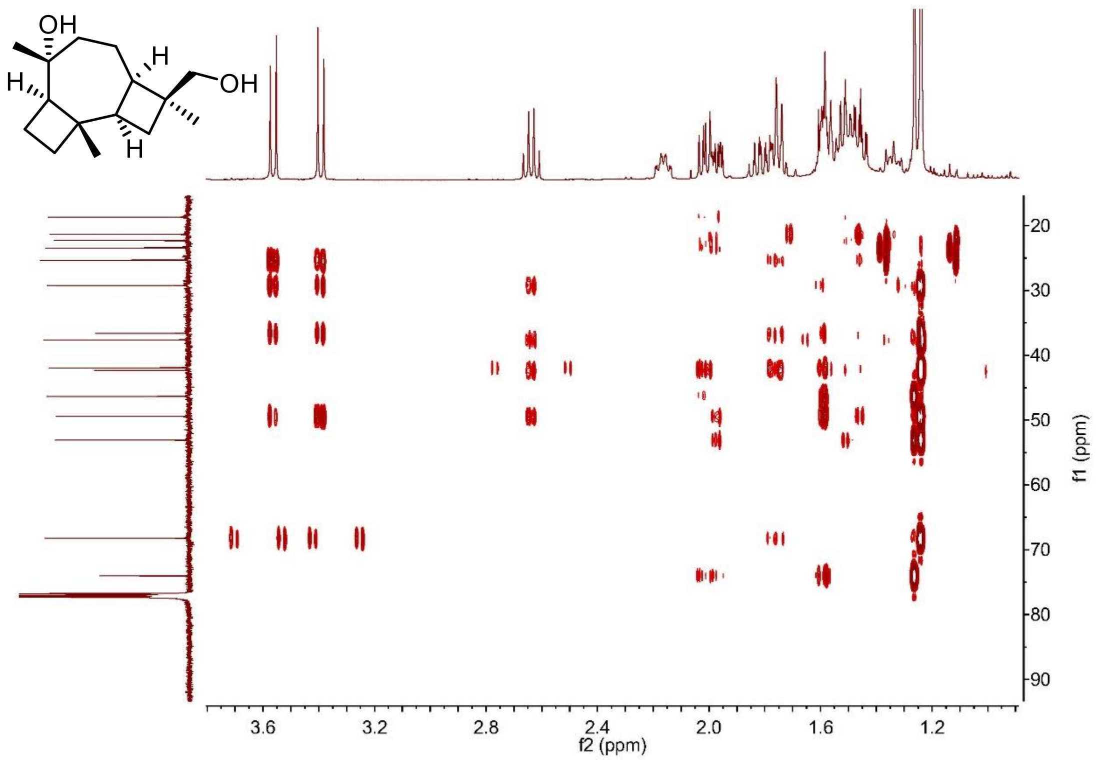

Figure S45. HMBC spectrum of **5** ($CDCl_3$)

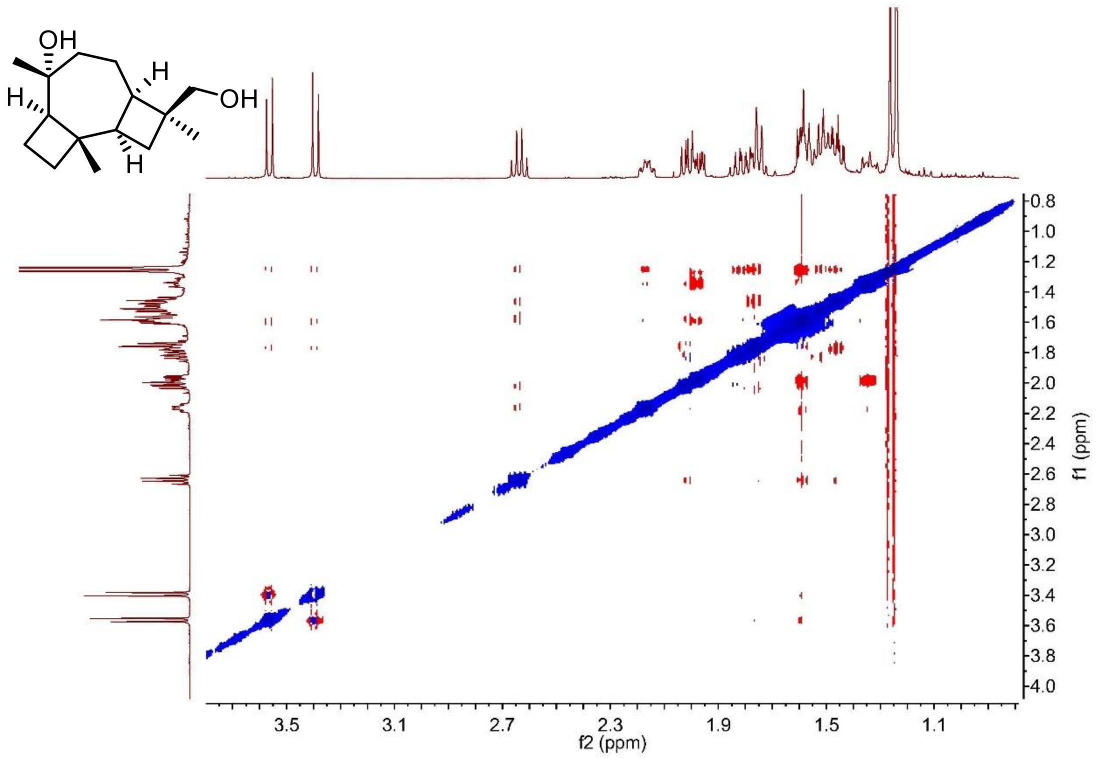

Figure S46. NOESY spectrum of **5** ($CDCl_3$)

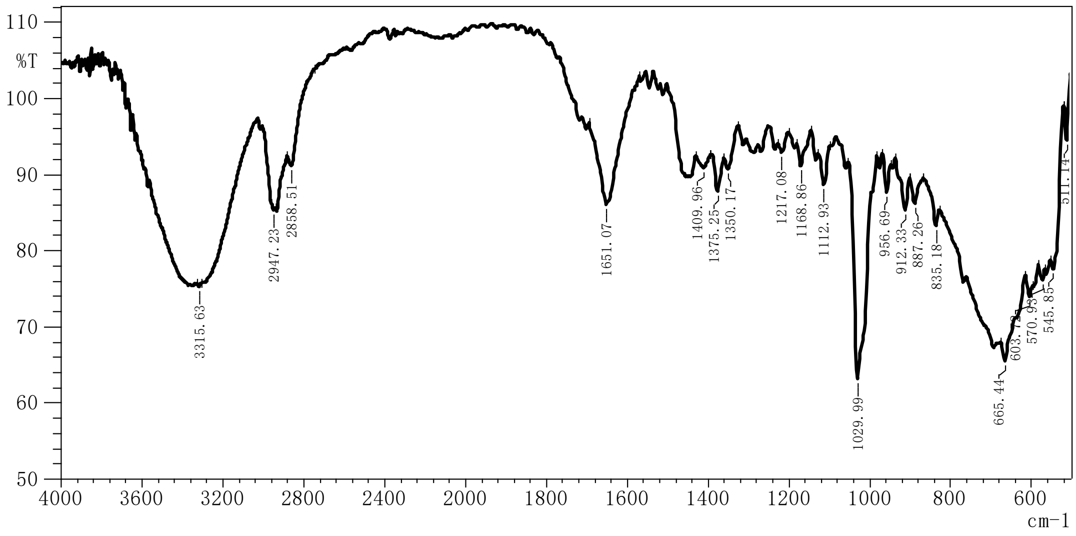

Figure S47. IR spectrum of **5**

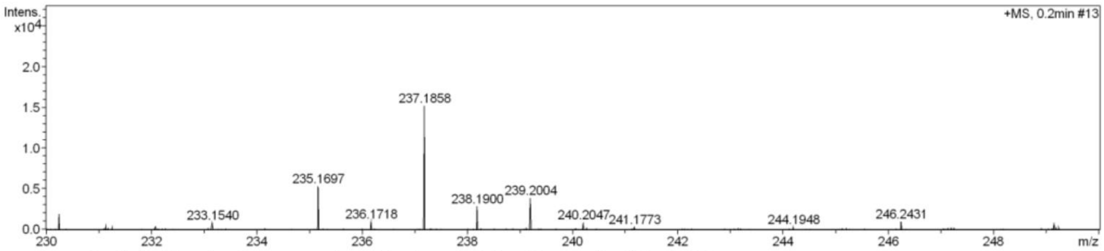

Meas. m/z	#	Ion Formula	Score	m/z	err [ppm]	err [mDa]	mSigma	rdb	e⁻ Conf	N-Rule
203.1802	1	C15H23	100.00	203.1794	4.0	0.8	14.4	4.5	even	ok
221.1906	1	C15H25O	100.00	221.1900	2.9	0.6	15.5	3.5	even	ok
239.2004	1	C15H27O2	100.00	239.2006	-0.8	-0.2	31.2	2.5	even	ok

Figure S48. HRESIMS spectrum of **5**

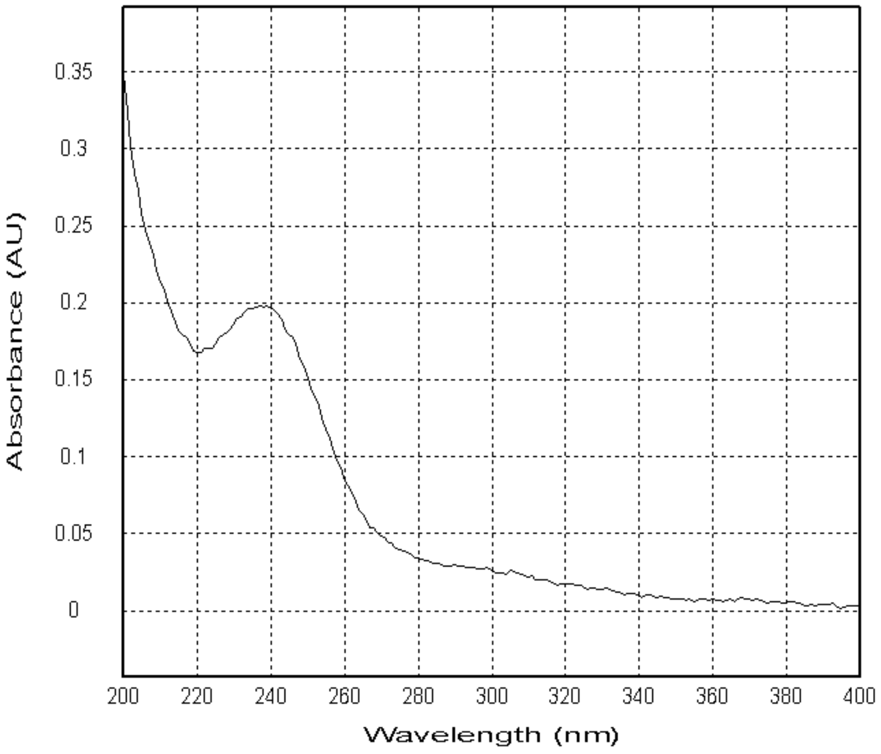

Figure S49. UV spectrum of **5**

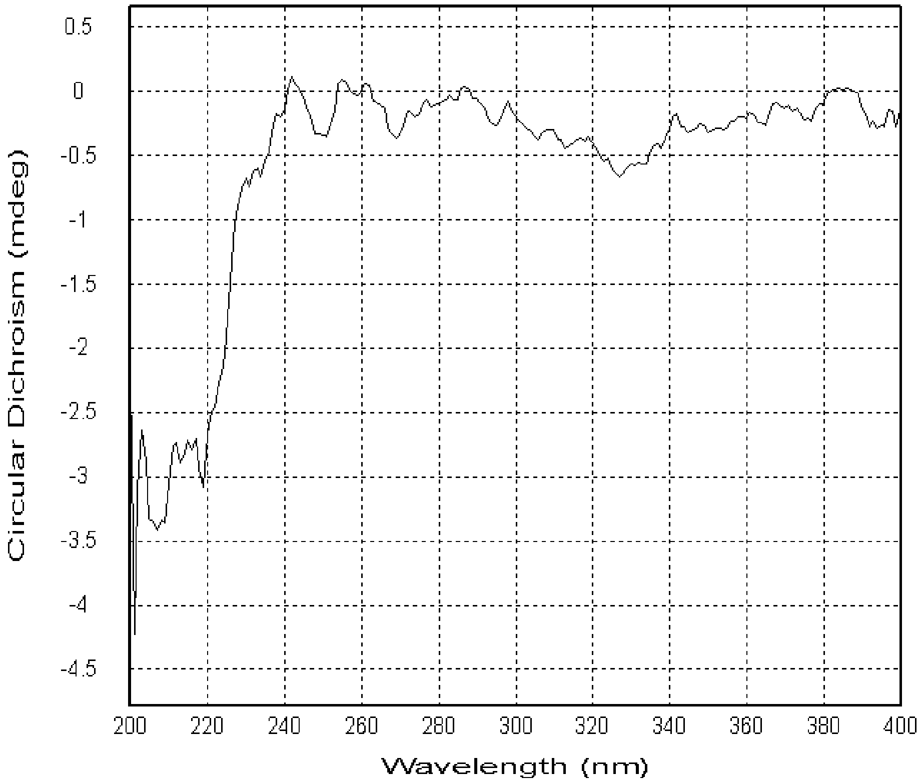

Figure S50. CD spectrum of **5**

Table S1. X-ray crystallographic data and structure refinement for **5**

Identification code	**5**
Empirical formula	$C_{15}H_{26}O_2$
Formula weight	238.36
Temperature/K	100.00(10)
Crystal system	trigonal
Space group	$P3_2$
a/Å	13.6894(2)
b/Å	13.6894(2)
c/Å	6.60290(10)
α/°	90
β/°	90
γ/°	120
Volume/Å^3	1071.60(4)
Z	3
ρ_{calc}g/cm^3	1.108
μ/mm^1	0.552
F(000)	396.0
Crystal size/mm^3	0.2 × 0.1 × 0.1
Radiation	CuKα (λ = 1.54184)
2Θ range for data collection/°	7.456 to 148.49
Index ranges	$-16 \leqslant h \leqslant 15, -17 \leqslant k \leqslant 15, -8 \leqslant l \leqslant 7$
Reflections collected	7072
Independent reflections	2779 [R_{int} = 0.0298, R_{sigma} = 0.0367]
Data/restraints/parameters	2779/1/159
Goodness-of-fit on F^2	1.156
Final R indexes [I>=2σ (I)]	R_1 = 0.0422, ωR_2 = 0.1073
Final R indexes [all data]	R_1 = 0.0444, ωR_2 = 0.1085
Largest diff. peak/hole / e Å^{-3}	0.18/-0.17
Flack parameter	-0.05(12)
