# Supplementary figures and images for: Five new secondary metabolites from an endophytic fungus Phomopsis sp. SZSJ-7B
Source: Front Plant Sci. 2022 Nov 14;13:1049015. doi: 10.3389/fpls.2022.1049015 (PMC9702824; doi:10.3389/fpls.2022.1049015)

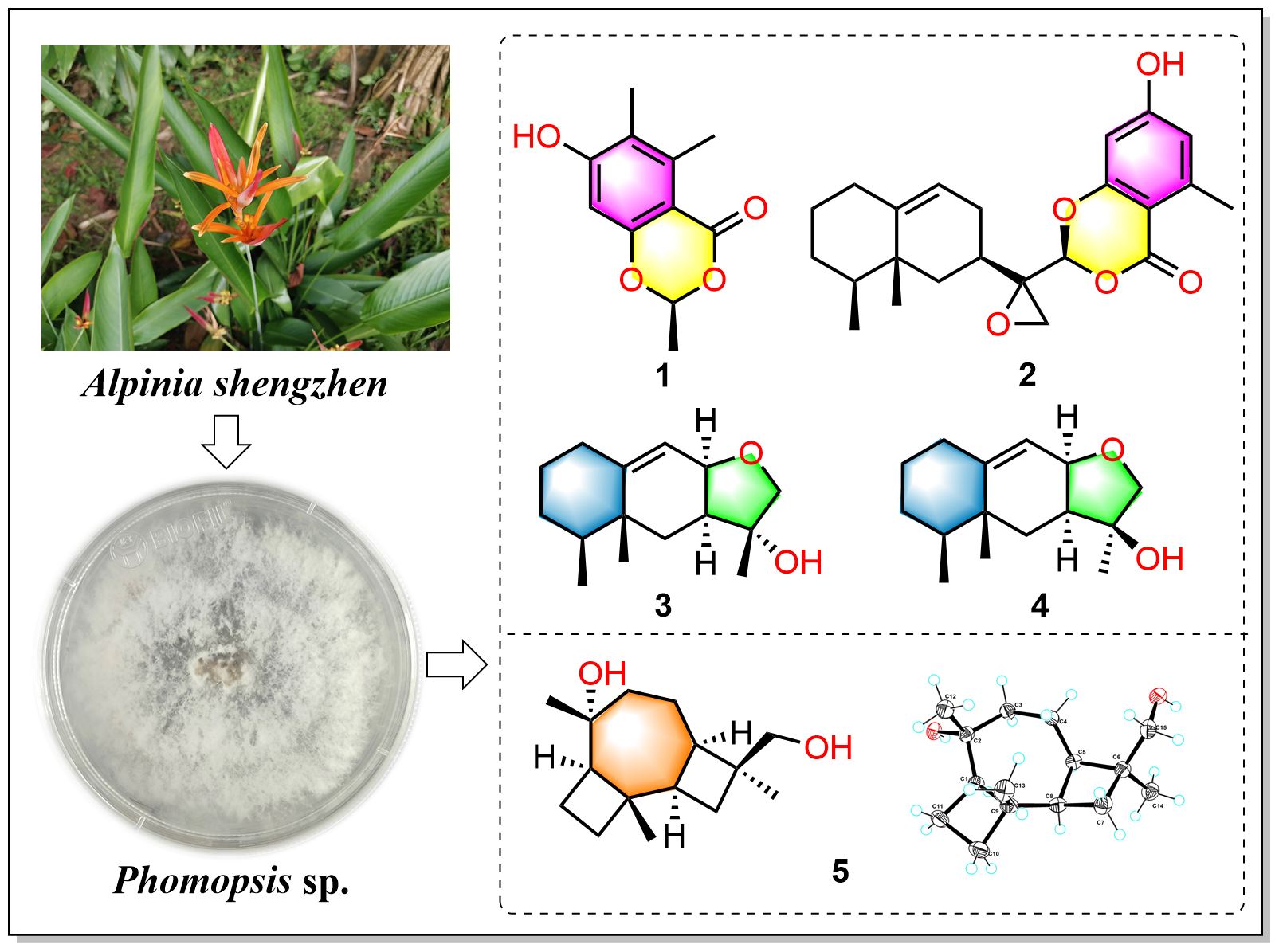

Supplement: Supplementary file 2 [file Image_1.tif]
